# Interventions targeting identity in adults with psychosis, severe mental illness, brain injury, or intellectual disability: a transdiagnostic scoping review

**DOI:** 10.3389/fpsyt.2026.1674898

**Published:** 2026-02-05

**Authors:** Uupke Elizabeth Kronemeijer, Gerdina Hendrika Maria Pijnenborg, Johanna Karina Muthert, Ellie Richtje Hennie van Setten, Lisette van der Meer

**Affiliations:** 1 Department of Clinical and Developmental Neuropsychology, University of Groningen, Groningen, Netherlands; 2 Department of Rehabilitation, Lentis Center for Mental Health Care, Zuidlaren, Netherlands; 3 Department of Long-term Care, GGZ Drenthe, Assen, Netherlands; 4 Department Comparative Study of Religion, University of Groningen, Groningen, Netherlands; 5 Independent researcher, Amsterdam, Netherlands

**Keywords:** acquired brain injury, identity, intellectual disability, intervention, personal recovery, self-concept, severe mental illness, psychosis

## Abstract

**Objective:**

Despite the extensive literature linking identity experiences with Severe Mental Illness (SMI), little is known about interventions specifically addressing identity in this population. This scoping review aims to explore the available literature on identity interventions whilst adopting a transdiagnostic approach. We provide an overview of intervention studies targeting identity or self-concept in individuals with psychotic spectrum disorders or severe mental illness (SMI), acquired brain injury (ABI), or intellectual disability (ID).

**Methods:**

A search was conducted across six databases for articles up to November 2025. Eligible studies were peer-reviewed and evaluated interventions for individuals with SMI, ABI, or ID. These interventions either focused upon identity or assessed outcomes specifically related to identity. Case studies, theoretical papers, and reviews were excluded. Both qualitative and quantitative data were summarized, emphasizing study approaches, intervention approaches, theoretical frameworks, outcomes and recommendations for future studies.

**Results:**

A total of 35 studies were identified: 16 on SMI/psychosis, 17 on ABI, and 2 on ID. There was substantial heterogeneity across interventions. Interventions aimed to help individuals become more positive, develop complex identities, adjust to illness, reconstruct integrated self-narratives, and experience an empowered (social) identity. Participants learned through experience, social connection, self-reflection, and narrative- and cognitive restructuring.

**Discussion:**

This review identifies several promising intervention elements for future research, as discussed in the paper. However, considering the limited number of studies, their heterogeneity, and the exploratory nature of this research, further investigation is required to build upon and expand existing approaches.

## 1 Introduction

Numerous studies have documented identity challenges in individuals with Severe Mental Illnesses (SMI) (1–4). Experiences of feeling disconnected from oneself during illness, shattered future plans, hospitalization, encountering (self)-stigma, relational changes, illness-centered self-perceptions (e.g. identity influenced by self-stigma or primarily constructed in terms of negative illness-related self-aspects), and disruptions in narrative identity (e.g. lack of temporal coherence, negative emotional valence) affect identity in the context of living with SMI (1–4). Despite the extensive literature linking identity experiences with SMI, little is known about interventions specifically addressing identity in this population, leading us to explore the available literature on identity interventions whilst adopting a transdiagnostic approach.

### 1.1 Identity in the current review

Identity and self-concept refer to who an individual is and how individuals perceive themselves both personally and within various social contexts (cf (5, 6). Although these terms are often used interchangeably, the term “self-concept” can be confusing for two reasons: it may refer to an overall self-evaluation (e.g., global self-concept) rather than a broader, multi-component view of the self, and it also tends to emphasize the cognitive organization of self-representations (7). In this review, we primarily use the term “identity”, which typically refers to multifaceted self-aspects, including unique characteristics and commitments (personal identities), as well as social roles and group memberships (social identities) (5, 7, 8). Various salient self-aspects can pop up in relation to the question “Who am I?” including names, roles, commitments, characteristics, self-perceptions, gender, worldviews, life goals, interests, cultural background, social groups, and relationships. These elements, implicitly or explicitly, shape how individuals define themselves, contributing to a multifaceted identity that may be reflected in one´s narratives, self-definitions and mental self-concept. When referring to identity in this review, we do not aim to capture a real “inner ego”, phenomenological “I”, or core experience of a “basic self” (cf (9, 10). Rather, we are interested in how interventions can influence the self-aspects people evaluate as important to their identity and how they understand and experience identity amidst enduring illness.

In narrative research, identity is viewed as a dynamic process, evolving through the stories individuals construct and re-interpret in various social contexts (11, 12). People may integrate personal stories into a coherent life narrative, providing direction and meaning (11). Although a sense of stability can be important to find meaning, identity is never static. People continuously experience life events, lose and gain social roles and commitments over time, and continuously re-interpret their experiences, life stories and the effect upon their identities -through constant dialogue with themselves and others- (12–14). Major life events (such as psychosis or stroke), can disrupt this process, making it difficult to incorporate the experience into one’s identity, potentially leading to a sense of ‘identity-theft’ (15). Such events may also reshape social connections, leading on the one hand to a loss of valued social roles and, on the other, to the acquisition of a new (and often stigmatized) identity: that of a patient. This social dimension of identity and identity change is central to the social identity approach to health (SIAH) (16). According to this framework, identifying with multiple social groups that are positive and mutually compatible supports health and wellbeing, whereas identification with compromised or stigmatized groups can negatively influence wellbeing (16). The Social Identity Model of Identity Change (SIMIC) further specifies how identity continuity, the acquisition of new positive group memberships, and the compatibility between old and new identities shape adjustment following major life events (16, 17).

### 1.2 Severe mental illness and identity change

Understanding identity change in individuals with SMI is crucial, as these individuals face enduring psychiatric conditions that significantly impact their healthcare needs, daily functioning, and identity (1, 18). Most individuals categorized under SMI have a diagnosis of schizophrenia, psychotic spectrum disorders, or enduring bipolar- or psychotic depression (18). Though the lifetime prevalence of these disorders is relatively low (19), their disability burden is high, often leading to long hospitalizations (20). This burden is not solely due to psychiatric symptoms but is exacerbated by challenges like social isolation, losses, unemployment, cognitive impairments, loneliness, (self-)stigma, existential distress, demoralization, disruptions in basic self-experience and narrative identity (1, 2, 10, 21–29).

Coping with major life changes, suffering, illness and stigma can make it difficult to maintain a coherent, meaningful, and positive identity. Individuals may lose valued social identities and instead adopt compromised ones that conflict with positive self-views or other social identities (16). Stressful events that challenge personal worldviews can create distressing discrepancies, triggering the need for meaning-making (30). Kaite et al. (2015) describe how significant life changes after illness can lead to feelings of disruption, confusion, and loss of pre-illness identity (3). In some cases, illness and patient roles dominate one’s identity (“illness engulfment” (31)), while others experience post-psychosis growth, developing a stronger sense of self or strengthening social connections (32). Recovery from illness involves re-establishing a relationship with the self, the illness, and the world, focusing on rebuilding a new sense of self and purpose (33, 34). Individuals with SMI emphasize the importance of developing or maintaining a non-stigmatized, multidimensional identity within this process of personal recovery (35) with core elements such as Connectedness, Hope, Identity, Meaning, and Empowerment (CHIME; 35). Supporting personal recovery does not mean getting rid of illness identity altogether, as it can remain an integral part of many people’s self-concept (36). Instead, it requires integrating illness-related and other experiences into a broader identity, allowing other self-aspects—such as social roles, interests, and goals—to become prominent again.

### 1.3 Focus of the scoping review

Despite increasing attention to identity and recovery in people with SMIs, a recent review of recovery-oriented studies found no intervention studies that (quantitatively) measured identity outcomes (37). While the CHIME framework highlights identity as a key element in personal recovery (38), evidence for specific identity-based interventions in SMI populations is limited. Moreover, there are few identity-related interventions for people with SMI that consider the cognitive and communication impairments that many people with SMI cope with (22, 39–41). Thus, it is particularly relevant to explore alternative interventions that are better equipped for people with cognitive problems. Given the increasing evidence that identity disturbances occur across diagnoses we therefore chose to adopt a transdiagnostic approach (42). Transcending diagnostic boundaries can help identify mechanisms that support identity recovery across different groups where identity challenges are prominent. Hence, in addition to studies in the SMI population, this review included literature on identity interventions in groups in need of a more non-verbal approach considering cognitive limitations (people with Intellectual Impairment; ID) and groups coping with sudden impairments that potentially alter their sense of identity (people with Acquired Brain Injury; ABI) (42, 43). Though individuals with ID or ABI face different challenges from those with SMI, there are common struggles across these groups. A shared issue is the impact of social and functional impairments on autonomy and societal participation (43). In addition to having impairments, these individuals must also relate to having a diagnosis – and this diagnostic label is frequently experienced as highly stigmatizing (1, 44, 45). Given the lack of clarity in the broader literature on identity interventions, a scoping review is the most suitable approach to systematically map the available research (46).

This review has two main objectives. First, it aims to provide an overview of interventions targeting identity in individuals with SMI, ABI and ID. The review summarizes each intervention’s goals, content, outcomes, user experiences, theoretical assumptions (e.g., identity interpretation), and mechanisms (recommendations from the study authors or participants regarding the intervention’s content or delivery, and hypotheses from the study authors or participants about barriers, facilitators, and mechanisms that impact the implementation, uptake, or effectiveness of the intervention). Second, it explores how these findings can inform treatment for people with SMI, supporting the development of new interventions. To our knowledge, no other reviews have been published with this specific transdiagnostic focus on identity interventions.

## 2 Methods

The scoping review followed the PRISMA-ScR guidelines (47). A protocol was developed before the study in line with JBI methodology for scoping reviews (48) and registered on OSF after finalizing the search strategy (49). Any deviations from the protocol are detailed in **Supplementary File S1**. Screening and data extraction were conducted by the first author (UEK) and research assistants (master’s students). Deviations were checked, and if substantial, a third screening or data extraction was done by a third reviewer (PhD student or last author). The process was regularly discussed within the research team, which consisted of five Dutch researchers who had working experience in care institutions for individuals with SMI and represented diverse perspectives (psychology: UEK, GHMP, ERHvS, LvdM; spiritual care (50): JKM; experience expertise: ERHvS). The data-extraction form prompted the reviewers to make reflexive notes, for example about CHIME themes, study interpretations and limitations, and contextual factors.

### 2.1 Search strategy

The databases PsycInfo, Medline, Web of Science, CINAHL, Academic Search Premier, and the Psychology and Behavioral Sciences Collection, were searched by title and abstract on November 11^th^, 2021, and updated November 10t, 20233, without any limitations on the start publication year. Dissertation abstracts from EBSCO open dissertations were retrieved for background literature up to 2021 but excluded from the updated search as the inclusion criteria only permitted peer-reviewed articles. The comprehensive search strategy is detailed in the online protocol (49). The search combined three English search strings: a) relevant concepts (e.g., identity, self-concept), b) interventions or evaluation methods, and c) the three target groups. No additional publications were found through expert consultation, but cross-referencing provided further articles.

### 2.2 Inclusion and exclusion criteria

Below we summarize the key inclusion and exclusion criteria. For a more detailed explanation we refer to the online protocol (49), whereas a summary and a list of refinements made after the protocol publication are presented in **Supplementary File S1**. Examples of excluded studies are presented in **Supplementary File S2**.

Participants: Studies were included if at least 50% of the participants were adults (≥18), as identity in children involves different concepts and interventions (e.g., school-based). At least 50% of the participants had to be characterized primarily as having a SMI, psychotic spectrum disorder (e.g. schizophrenia, bipolar disorder, psychotic depression), ABI, or ID.

Interventions: The review considered various interventions, including therapeutic interventions, but also activities in a non-therapeutic setting (e.g., a workshop in a community center), self-directed activities (e.g., a game or self-help), or programs and activities guided by volunteers, peers or non-healthcare professionals. We excluded activities such as volunteering, occupation, housing, self-advocacy, hobbies, and diagnostic tests, as they were not consistently viewed as interventions by all screeners, and did not specifically aim to target identity (re)building. General care approaches like rehabilitation or non-specific broad therapies without a description of specific intervention elements were also excluded.

Topics: Interventions needed a clear, pre-specified aim to either target identity or to evaluate identity (or self-concept or a relevant synonym) (49). Articles that focused exclusively on one aspect of identity (e.g., gender identity) were excluded. Studies were also excluded if they only evaluated micro-processes of identity (e.g., focused solely on an analysis of moment-to-moment identity affirmations within a single interaction), or only measured structural aspects of self-concept (e.g. degree of certainty about the self-concept, organization of the self-concept) – except if the intervention explicitly targeted identity and the study could be included based on that criterium. Studies describing interventions that only focused on an identity-related topic or focused on activities related to identity exploration that were mainly used as a means towards another end (e.g., goal setting, stigma) were also not included, except if there was a pre-specified aim to evaluate identity.

Sources and type of studies: Only peer-reviewed original research formally evaluating intervention outcomes or usability was included. This encompassed clinical trials, pre-post studies, pilot studies, multiple-case studies, and qualitative evaluations. We excluded brief reports without methods, opinion papers, editorials, conference abstracts, book chapters, theoretical articles, reviews, and dissertations. N = 1 case studies were also excluded, due to their large number, heterogeneity, and limited generalizability. Although there were no language restrictions, search terms were in English.

### 2.3 Study selection

Studies were deduplicated in Excel and Rayyan (51). Decision rules were piloted through independent blind screening until interrater agreement reached 75%. The first author (EK) and a research assistant then conducted a blind screening in Rayyan, with most disagreements resolved by consulting the last author (LM) (49). Complex cases were discussed in consensus meetings, leading to few refinements in decision rules (described in **Supplementary File S1**). In **Supplementary File S2**, a list of excluded articles is provided, highlighting studies that were excluded despite their relevance to the field and clarifying the reasons for exclusion in cases that were most difficult to decide or involved reviewer disagreements. In accordance with scoping review guidelines (52), studies were included regardless of methodological quality.

### 2.4 Data extraction

The data extraction sheet was partially based on the Template for Intervention Description and Replication (TIDieR) checklist (53), covering intervention design, theoretical background (e.g., rationale, identity definitions), materials, procedures, facilitator expertise, and delivery. Additional categories included participant characteristics (e.g., gender, age, diagnosis, IQ), intervention context (e.g., country), methods (e.g., study design, outcome measures), and results. Facilitators, barriers, and future recommendations were also noted (based on results and discussion sections), alongside reflections on applicability for people with cognitive deficits. After piloting the data extraction process, two independent reviewers conducted the data extraction. Original and final extraction forms are available online (49). In order to evaluate included study quality, we used the Mixed Methods Appraisal Tool (54).

### 2.5 Data analysis

Both qualitative and quantitative data were extracted. Qualitative findings were summarized or quoted in the data extraction form. No formal meta-synthesis was conducted, but findings of qualitative studies were contextualized in the summary tables. Any part of the results section that involved integrating qualitative data (e.g., intervention topics, intervention barriers, and identity approaches) was developed through a systematic, iterative review of both the original studies and the data-extraction summaries provided by the two reviewers. The analysis focused on literal descriptions, rather than interpreting latent meanings.

## 3 Results

### 3.1 Search and selection of studies

**Figure 1:**
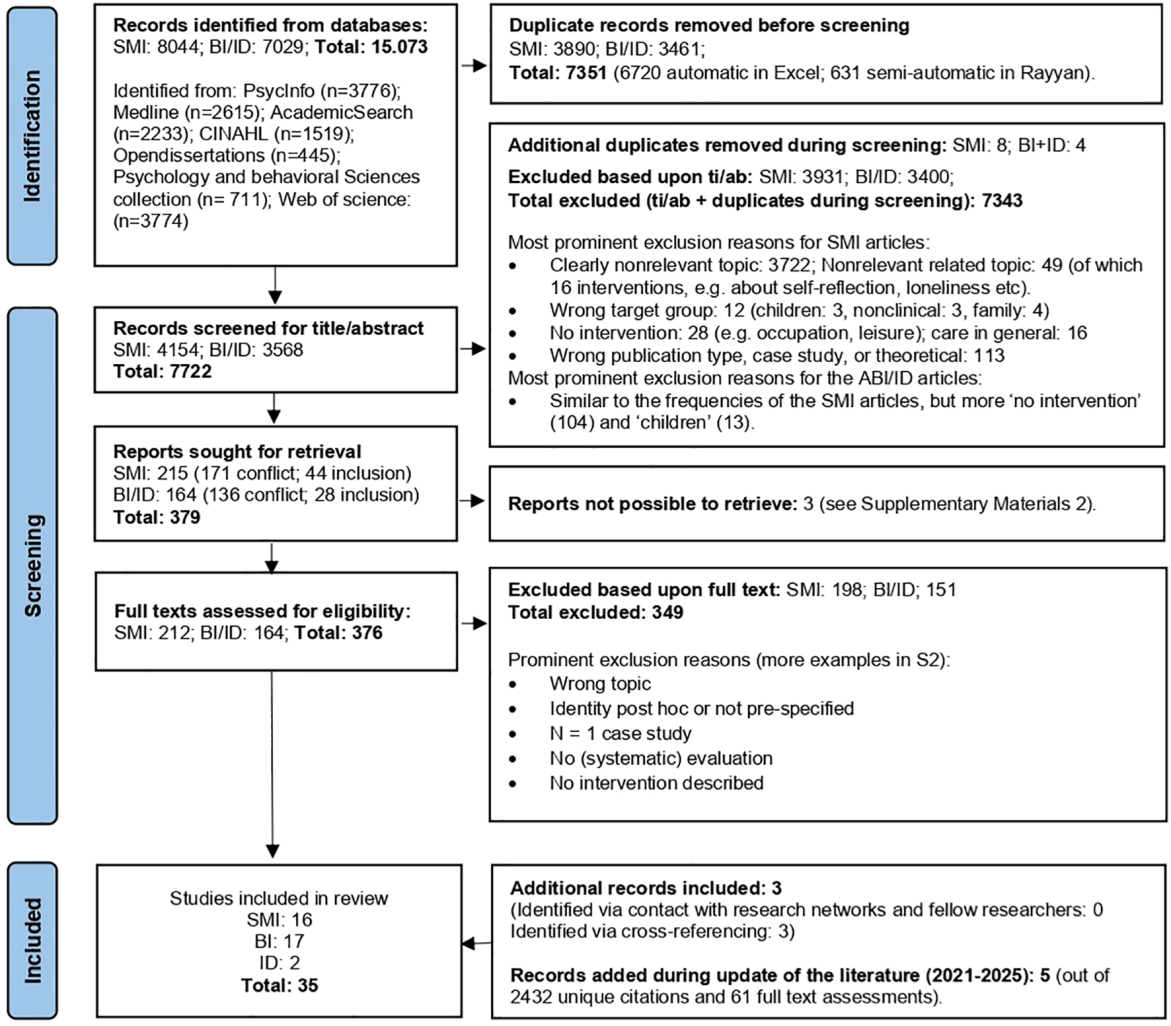
Prisma flow chart. Adapted from Page MJ, et al. BMJ 2021;372:n71. doi: 10.1136/bmj.n71.

A search in November 2021 resulted in 15.073 hits, with 7722 unique articles remaining after deduplication. Updated searches in November 2023 and November 2025 added 1,062 and 1,370 new articles, respectively. Most abstracts (89%) were marked as irrelevant, as they did not focus on interventions or identity. Many only briefly mentioned identity (e.g., to introduce the target group) or used the term in unrelated contexts (e.g., male identity, national identity). Studies outside the scope of this review often focused on related topics such as self-esteem, story-telling, stigma, metacognition, narrative enhancement, or basic self. Examples of excluded studies are presented in **Supplementary File S2**. Three articles were excluded due to unavailable full texts, two of which were non-English (see **Supplementary File S2**). A total of 437 full texts were assessed for eligibility. In total, 35 articles were eligible for review (SMI: *k* = 16, BI: *k* = 17, ID: *k* = 2), of which three were included through cross-referencing. The search process is shown in **Figure 1**, following the PRISMA guidelines (47).

### 3.2 General characteristics of the studies

#### 3.2.1 Study characteristics and design

Detailed characteristics per study are provided in **Supplementary File S5**. Here, we will provide a summary of these data. Of the 35 included articles, 22 were published after 2010, and eight between 1973-1995. All studies were conducted in high-income Western countries, with 16 from the United States. The review includes 22 studies with primarily quantitative outcomes, nine qualitative studies, three mixed-methods studies, and one case series. Eleven studies were explicitly labeled as pilots or feasibility trials. Two-thirds of the studies described interventions that were newly developed and unique, and the remaining interventions were also not yet well established, although some were adaptations or novel applications of previously tested techniques (e.g., mindfulness-based cognitive therapy, physical interventions, family-focused interventions, Groups for Health). Studies were included on a per-paper basis, meaning that papers describing the same group or intervention across multiple publications were not all included (see **Supplementary File S2**). Two interventions appeared in multiple studies included in this review (therapeutic songwriting and a group engulfment intervention (55–59).

The differences in study designs make it difficult to compare them, as even studies with similar outcome measures or qualitative approaches, often varied widely regarding target groups, intervention approaches, time of assessment, and data reporting. Moreover, the quality and study methodology of the studies renders it impossible to draw firm conclusions from the individual studies. Generally, sample sizes of the treatment groups in the studies were small (all studies: M 17.46, SD 11.3, range 3-45; quantitative studies: M = 18.5, SD = 10.4, range = 4-41), and just over half of the quantitative studies included a clinical comparison group (*k* = 14), with seven using an active comparison intervention beyond treatment as usual. Although seven quantitative studies reported random assignment to a control intervention or to treatment as usual, the randomization process was not described in three of them, and one RCT study used a control condition involving an alternative format of the target intervention. Among the quantitative studies, only four studies reported identity outcomes both post-intervention and at follow-up. Some studies were conducted 20–50 years ago, using different terminology, ethical standards, and scientific methodologies, and must be understood within the historical context of their time. Overall, the MMAT appraisal indicated that many quantitative studies showed important methodological limitations, including insufficient control of confounding variables, incomplete reporting of data, small sample sizes, or concerns about the validity of identity measures. The qualitative studies were generally of sufficient methodological quality, although one study showed weak methodological credibility. More detailed information on quality assessment can be found in **Supplementary File 6**.

#### 3.2.2 Participant characteristics

An overview of participant characteristics per study is given in **Supplementary Table S3** of **Supplementary Material 5**. Approximately 40% of participants were women. In ten studies, women were underrepresented (women/men ratio was < 1/4 in: SMI: *k* = 4, ABI: *k* = 5, ID: *k* = 1), while two studies included only women (SMI: *k* = 1, ABI: *k* = 1). Some studies focused on participants in their 20s and 30s (SMI: *k* = 3, ID: *k* = 1), and most included at least some young adults aged 26 or younger (SMI: *k* = 7, ABI: *k* = 8, ID: *k* = 1). A few studies (SMI: *k* = 2, ABI: *k* = 5, ID: *k* = 1) included senior adults (67+ years), but none specifically targeted them. Intelligence scores were rarely mentioned (SMI: *k* = 1, ABI: *k* = 2, ID: *k* = 1), but most studies included individuals with varying cognitive functioning. However, two SMI studies excluded those with intellectual disabilities, seven ABI studies excluded participants with severe disabilities or severe aphasia, and ID studies primarily involved individuals with mild or borderline learning disabilities. Studies varied in illness severity, independence, and living situation of participants. Some focused on specific groups, such as those with significant impairments, challenging behavior, first-episode psychosis, LGBTQIA+ individuals, veterans, or people with aphasia. SMI studies generally involved individuals with SMI, first-episode psychosis, or schizophrenia, though none specifically targeted bipolar disorder or psychotic depression. ABI studies commonly included individuals with traumatic brain injuries or cerebrovascular accidents.

### 3.3 Identity in the studies

We categorized studies based on the focus on identity in interventions. Some interventions explicitly focused on identity development through themes like self-concept, self-esteem, and life narratives (“direct identity interventions”^1^: SMI: *k* = 10, ABI: *k* = 9, ID: *k* = 1), whereas others targeted identity indirectly through activities like sports or mindfulness or did not describe identity as a major intervention aim (“indirect identity interventions”: SMI: *k* = 6, ABI: *k* = 8, ID: *k* = 1).

**Figure 2:**
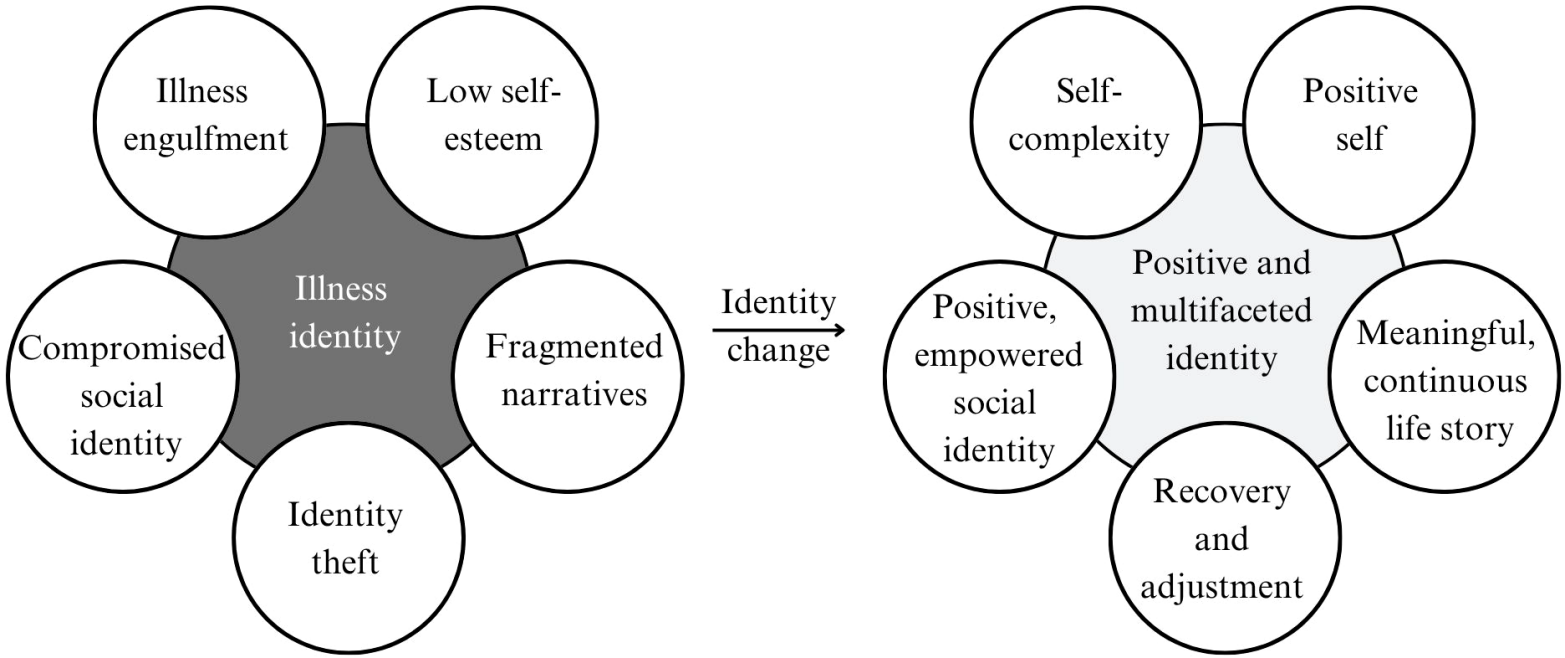
Identity conceptualizations and aims commonly employed in the studies.

The identity conceptualizations and aims varied across studies. To understand identity conceptualizations, we analyzed hoped-for identity changes, underlying the theoretical frame of the intervention, outcome measures or intervention components (see **Supplementary File 5** for details). **Figure 2** summarizes the five most common hoped-for identity changes. Firstly, many interventions conceptualized identity change as a movement from illness engulfment - a self-concept defined by illness- towards self-complexity. Examples of specific terminology used to describe this are, illness-identity, illness centrality, role-constriction, identity complexity, and multidimensional- or plural self. Secondly, many studies aimed to change low self-esteem and negative (often stigmatized) self-views to move towards self-evaluations that are more positive. This movement is related to concepts such as self-worth, self-stigma/internalized stigma, decentralization of negative self, and importance differentiation of positive and negative self-aspects. A third movement was present in the narrative studies, which often aimed to change fragmented stories and help participants reconstruct integrated, continuous and meaningful life stories. These studies observed a biographical disruption, problem focused life narrative or difficulty to integrate illness in views about current and future self, and aimed for biographical repair, development of alternative stories, residual self, subjugated stories, liberating stories, continuous life stories, self-continuity, or cross-temporal identity. Other studies describe identity change as a movement from loss and general disruption after illness (e.g. the feeling of ‘identity theft’) to feeling able to adjust and recover. These studies broadly described identity struggles as feelings of unbalance, identity threat, identity disruption, or a shattered self, and aimed for the development of a balanced, congruent and integrated self-view - for example through growth, adjustment to changed life circumstances and re-evaluation of self, illness and values. Lastly, some studies employed a more social focus, aiming to change compromised social identity and helping participants to feel empowered, included and positive about their social (illness) identity. These studies focused on many different aspects, such as exclusion, inequality, stigma, social roles, group identification, stories about gender or sexual identity, self-advocacy, sharing illness experiences and empowering stories with others, decision-making, independency, and equality between individuals with and without illness.

### 3.4 Identity evaluations

The most frequently used quantitative identity outcome scales measured how a person feels about different self-concept domains (Tennessee Self Concept Scale: TSCS, *k* = 10 (60, 61); illness engulfment (Modified Engulfment Scale: MES, *k* = 5 (31);), or self-attributes in the present, and/or past or future (Head Injury Semantic Differential Scale: HISDS, *k* = 5 (62);). Three studies evaluated identity quantitatively through a proxy. Qualitative evaluations were collected post-intervention (*k* = 6), pre- and post-intervention (*k* = 2) or throughout the study (*k* = 4), through interviews or written feedback (with the type of question varying, e.g., open-ended, close-ended, and direct or indirect questions about identity), as well as through observations. Examples and details about the evaluation methods are presented in **Supplementary File 5**.

### 3.5 Intervention categorization and content

#### 3.5.1 General intervention characteristics

Details on the intervention aims, delivery mode, intervention duration, procedures, and suitability of the intervention for individuals with cognitive difficulties are presented in **Supplementary File 5**. SMI interventions ranged from 3–36 hours, ABI interventions from 6–28 hours and ID interventions from 6–9 hours, with the majority of the interventions having a duration between 10–20 hours. Duration of SMI interventions was highly variable, as some structured direct identity interventions were developed to be short, and some intense indirect therapeutic programs lasted over a year or involved multiple sessions per week for several months. Nineteen studies were mainly provided in a group, three both in a group and individual, twelve were mainly individual (of which three offered one group meeting) and one was a tool which could be used with one or more partners of choice. In two interventions, family members were involved, one intervention was possible with a partner of choice, in one intervention family and friends were invited to one session, and in five interventions family members or friends were invited to a presentation at the end of the intervention. One intervention was co-led by a lived-experience researcher, and several interventions were largely client-driven and peer-based, though this was mostly in the ABI interventions. Facilitators came from diverse backgrounds such as psychology (*k* = 10), speech-language therapy (*k* = 6), occupational or recreational therapy (*k* = 3), arts/music therapy (*k* = 4) and social work (*k* = 2); however, the background of therapists was also often not explicated. Common therapeutic approaches included narrative therapy (*k* = 11) and cognitive-behavioral or acceptance-based therapy approaches (*k* = 7).

#### 3.5.2 Recurring topics addressed during the interventions

In **Supplementary File 5** we provide a more detailed overview of the topics discussed in the interventions. Common discussion topics that recurred multiple times both within and across target groups included different self-aspects or life domains (e.g. social self, work/academic/leisure activity), life stories, self-esteem, healthy self-concept/strengths or constructive thoughts, values/moral self, feelings, future, perception of physical or bodily self, delegitimization/stigma, adjustment/recovery, coping or dealing with specific illness-related difficulties, illness (interpretation and influence), social/family relationships and interpersonal skills. Additionally, topics that explicitly recurred in multiple SMI studies but in less than two ABI/ID studies included positive beliefs/dreams, goal setting, mindfulness/meditation, stress, and assertiveness; whereas topics that recurred in multiple ABI/ID studies but in less than two SMI studies included ‘future self-stories and continuity between future and present/past’, communication skills and sexuality/gender.

#### 3.5.3 Differences between target groups

Most studies involving individuals with SMI used quantitative outcome measures and were primarily focused on talk-based or reflective interventions, though many also incorporated action-oriented elements like mindfulness or creative exercises. Although many SMI interventions were group-based, most studies did not emphasize, peer support and social connection in the intervention description or analysis. Session activities were typically guided by the therapist and generally took place in a therapeutic setting. In contrast, nearly half of the studies involving ABI and ID relied predominantly on qualitative identity evaluations. Compared to the SMI studies, the ABI/ID studies included more interventions that were loosely structured, emphasized peer support, highlighted participant involvement in selecting group topics, and incorporated a focus on story sharing/advocacy elements aimed at individuals without ID/ABI. A third of the SMI articles were (partly) based on CBT principles (SMI: *k* = 5, ABI: *k* = 1, ID: *k* = 0), and all of the CBT-informed studies were analyzed quantitatively. More than a third of the ABI/ID articles mentioned narrative principles (SMI: *k* = 3, ABI: *k* = 7, ID: *k* = 1), and half of the studies with a narrative approach were evaluated qualitatively.

### 3.6 Identity effects of interventions

Distinguishing between approaches was difficult due to the multifaceted nature of the interventions. Most interventions had unique key features not found in other interventions, whether within the same or different target groups. In the narrative summary below, we categorize interventions into three groups: project-based interventions with creative output, specific activity-based interventions, and therapeutic/peer support interventions. We focus on identity outcomes of direct interventions and summarize the indirect interventions shortly. For a complete study overview, we refer the reader to the **Supplementary Materials**. Quantitative findings are described in **Supplementary File 3** and qualitative findings in **Supplementary File 4**.

#### 3.6.1 Identity-focused project-based interventions with creative output (*k* = 5)

Project-based interventions focused on creating a specific creative output, such as a song, artwork, or lived experience presentation.

SMI (*k* = 1): Recovery Narrative Photovoice. From the SMI studies, only one direct study was classified as a project-based creative intervention. In this intervention, participants with SMI created photographs that were showcased in a public exhibition, where they could share their recovery stories (63). The 10-week program focused on recovery, empowerment, and community integration, through elements of narrative therapy and photovoice. Although the recovery-narrative photovoice intervention was designed to target identity, the study did not include an evaluation of identity-related outcomes. Nevertheless, the intervention appeared to be both feasible and acceptable to participants.

ABI (*k* = 4): Therapeutic Songwriting and the ‘My Story Project’. Three ABI studies examined the same 12-session one-on-one therapeutic songwriting intervention inspired by narrative therapy, during which participants created songs about their past, present and future selves (55, 58, 59). One study used the HISDS to measure self-concept in individuals with ABI or spinal cord injury, finding a significant increase in positive self-concept, particularly in early sessions (55). Another songwriting study used a descriptive case-series with HISDS and TSCS scores to track identity changes in five men with ABI (58). This study reported mixed HISDS outcomes and stable or improved TSCS scores after the songwriting intervention, with more positive identity changes in those with milder symptoms. In the another songwriting study, the protocol was adapted for individuals with aphasia and a ‘release party’ was added as a thirteenth session - during which the songs were presented to invited guests (59). Like the other two songwriting studies, this intervention was offered to one participant at a time, but in this study not only a music therapist, but also three other practitioners were present, including a speech-language therapist. A qualitative evaluation with three participants using interpretative phenomenological analysis demonstrated the potential of self-focused therapeutic songwriting to support identity reflection, positive experiences of a relationship-based therapeutic approach, and the value of meaningful activities such as songwriting. Another direct ABI intervention study - conducted by the same first author as the aphasia songwriting intervention - also centered on storytelling but used a different medium, PowerPoint, to develop a story about identity before and after stroke (64). Participants met individually with a speech-language therapist to develop their narrative and presented this at the end of the intervention to the other participants and family. This intervention was also evaluated with interpretative phenomenological analysis of interviews with three participants, which showed that the intervention enabled participants to get positive reactions to their story, listen to others’ experiences, explore negative experiences and future perspectives, and gain communication confidence (64).

ID (*k* = 0). No direct ID project-based interventions with creative output were found.

#### 3.6.2 Indirect project-based interventions with creative output (*k* = 2)

SMI (*k* = 0): No indirect SMI projects with creative output were found.

ABI (*k* = 1): Developing a Self-advocacy Presentation. One indirect ABI project-based intervention was identified, which involved creating a presentation about lived experiences. Post-intervention interviews reported positive identity outcomes, even though identity was a secondary aim and no explicit focus of the intervention (65).

ID (*k* = 1): Mural Painting. One indirect ID intervention involved participants painting a mural for their school. While the intervention did not have a specific aim on identity, this study measured identity and self-concept domains with the TSCS (66). Most results were non-significant, with the exception of the TSCS social subscale, which significantly increased in the intervention group compared to individuals who did not receive the intervention.

#### 3.6.3 Identity-focused interventions focused on one specific activity or material (*k* = 5)

Specific interventions revolved largely around one specific exercise, material or tool, for example self-photographs and videos (67, 68), an identity-tool (41), a book about identity (69) or a strengths-exercise (70). Only interventions that did not have one specific creative product as output are described here.

SMI (*k* = 3): Self-experiential Images and the ‘This Is Me’ Tool. In two interventions (67, 68), both conducted in the 1970s, participants with SMI were shown photographs or videos of themselves, to target self-awareness and bodily awareness. These studies aimed to target self-concept, but the intervention mainly focused on observing aspects of oneself (e.g. physical or emotional). Results of the self-experiential interventions were mixed, with some participants experiencing discomfort or negative self-comparisons. In one study participants were photographed in each session and then shortly discussed the photograph, for example talking about likes and dislikes (67). Self-concept was mainly evaluated with a ‘draw-a-person’ test, where the authors judged the drawings made after the intervention to reflect significantly enhanced awareness of bodily boundaries and more positive self-representations (67). The other intervention compared three conditions; in the two self-experiential conditions participants watched videos of themselves in a group (focusing either on bodily awareness and self-other distinctions or on emotion recognition), and in the control condition they did social group activities (68). This study reports a significant interaction effect for self-concept, but unexpectedly this was due to a decrease in self-concept in the self-experiential groups and an increase in the control condition. According to the authors, this may be due to the confrontational nature of the intervention, increased awareness of negative self-aspects, or the therapist’s focus on correcting mistakes rather than fostering a therapeutic relationship.

One other SMI intervention used a specific tool/activity to focus on identity. In consultation with clients and other experts an activity tool, named ‘This is Me’, was developed to promote participation in identity-related activities with another individual or a group (41). Activities ranged from self-reflective tasks (e.g., drawing about identity) to interpersonal exercises (e.g., teaching a skill) and community engagement (e.g., visiting new places). The tool was mainly used by SMI service users alongside a mental health professional and was designed to foster equality and interaction. While generally well-received, some barriers were identified (see **Supplementary Files 4** and **5**). A qualitative evaluation highlighted its potential to enhance engagement in activities and shifts in roles and relationships. Although some participants also reported that the tool encouraged self-reflection, not all participants could recognize identity changes or reflect upon this.

ABI (*k* = 2): Bibliotherapy and a Signature Strengths Exercise. Hoover et al. (2023) conducted a qualitative evaluation of an online aphasia bibliotherapy group (69), using the book Identity Theft: Rediscovering Ourselves After Stroke (15). Participants with aphasia read the book and engaged in online group discussions, which promoted self-reflection by comparing their own experiences with those in the book. Peer interactions during the intervention fostered support, normalization, and a sense of friendship. Another ABI study evaluated a positive psychology program for individuals with ABI exhibiting challenging behavior (70). The authors also specifically aimed to evaluate one component of this intervention, namely the signature strengths exercises, which involves reflecting on personal strengths. This exercise appeared enjoyable and feasible with adequate support, but the statistical analysis (with four participants) did not yield significant results (70).

ID (*k* = 0). No studies describing direct ID projects with creative output were found.

#### 3.6.4 Indirect interventions focused on one specific activity or material (*k* = 6)

SMI (*k* = 3): Group Decision Making, Mindfulness-Based Cognitive Therapy, and Cognition Training. A study reporting on a decision-making group intervention found no significant difference in self-concept changes, as measured with the TSCS, between the intervention and control groups (71). A study about mindfulness-based cognitive therapy for individuals with psychosis evaluated changes in elements related to self, others and the psychosis experience, using constructs participants elicited themselves (e.g. ‘weak’ vs ‘strong’) (72). The study reported a significantly decreased difference between ideal self and self-as-recovered after the intervention, but no other significant increases in ratings of personal constructs elicited for self-related elements. Additionally, the authors noted increased correlations between the constructs that participants elicited after the intervention. Lastly, an indirect SMI study focusing on cognitive training found no significant effects on self-concept as measured with the TSCS (73).

ABI (*k* = 3): Sports and Communication Skill Training. Some positive identity changes were also reported in individuals with ABI after indirect interventions, such as sports interventions and a communication skill intervention (74–76). All of these three studies reported significant increases from pre-intervention to post-intervention in two or more sub-scales of the TSCS. However, for one of the physical exercise programs, self-concept was also measured with the Social Vocabulary Index, which showed an increase in self-concept, but a decrease in ‘view or value of the personalities of others’ and ‘increased uncertainty in self-perception’ (74).

ID (*k* = 0). No indirect ID projects focused on one specific activity or material.

#### 3.6.5 Other identity-focused therapeutic or peer support interventions (*k* = 11)

This category includes individual or group talking therapy, often combined with activities designed to promote self-reflection or cognitive or narrative restructuring. Though some interventions from other sections could also fit this label (e.g (15, 72)), we only discuss studies not covered in other categories here.

SMI (*k* = 6): Therapy Focused on Self-concept, Future self, Social Identity, or Engulfment. In addition to the four identity-focused therapies with a focus on a specific creative output or a specific activity or material, we found six other direct identity-focused SMI therapies. All of these interventions were relatively short (4–12 sessions) structured interventions which were led by a therapist, during which participants discussed identity topics and engaged in related exercises. One self-concept intervention took place in a group and was described as psychoeducational, experiential, structured, and non-confrontational, focusing on identity, strengths and self-esteem (77). The study did not formally evaluate identity, and there were no significant changes in self-esteem or confidence in either the intervention- or the control group. The authors note that most participants mentioned improvements in self-esteem, social confidence, and self-perception, but it remains unclear how this information was collected. Another study highlighted future self-concept and aimed to improve feelings of identity continuity and to reduce suicidality in veterans with SMI, through Continuous Identity Cognitive Therapy, which combined elements from cognitive behavioral therapy, acceptance and commitment therapy and narrative therapy (78). The intervention was feasible for individuals with different diagnoses, and overall participants showed increased future self-continuity and positive feelings toward their future selves, though no significant changes were observed in the vividness or similarity between present and future selves. Three other interventions centered on illness-engulfment, addressing how psychosis impacts identity, self-stigma, and self-esteem (56, 57, 79). One of these interventions was a four-session individual therapy for participants with enduring schizophrenia and high engulfment (SELF: Self-concept and Engagement in LiFe), during which individuals discussed the impact of psychosis, engulfment, and identity beyond illness – inspired by cognitive behavioral therapy principles and elements from narrative therapy and positive psychology (79). This intervention led to a significant reduction in illness engulfment compared to a waitlist control group. The other two engulfment studies were a pilot-study and RCT of an intervention which targeted engulfment, stigma and self-concept through 12 structured group sessions for young people with first-episode psychosis (56, 57). Both studies found significant decreases in engulfment, but TSCS self-concept scores did not significantly change in the RCT study (56, 57). Lastly, one randomized study compared group-based and individual formats of a five-session social-identity informed loneliness intervention (Groups for Health) adapted for individuals with psychosis (80). Participants received psychoeducation about social identity and the role of social group belonging for wellbeing, created social identity maps, and were supported in setting and achieving social goals. The study described some small positive shifts in the characteristics of participants’ social identity maps from the second to the final session; however, these changes were not statistically tested. More specific identity-related measurements focused on identification with “people who have unusual beliefs and experiences”: showing an increase over time in both in-group identification and identity integration. There were no significant group differences between the individual or group format (80).

ABI (*k* = 3): Therapy Focused on Self-concept, A Biographic–Narrative Intervention and a Women’s Self-help Group. One identity-focused intervention for people with ABI was comparable to the structured self-concept group interventions for SMI. Delivered in a semi-structured group, this intervention focused on identity (changes) after illness, aiming to develop a multifaceted and positive identity (81). The HISDS outcomes after the intervention showed significant improvements in self-concept, particularly in attractiveness, hopefulness, self-confidence, cooperativeness, and boredom. Two other direct ABI interventions emphasizing peer support and participant input were evaluated qualitatively. A biographic–narrative intervention for individuals with aphasia, combined individual sessions in which participants learned to talk about their life story, with more flexible group meetings about health and leisure (82). Qualitative accounts of the participants highlighted feelings of competence, the impact of the social interaction with peers, sense of control, taking up new activities, and a less deficit-focused self. Another peer intervention was a women’s self-help group with an emphasis on identity, empowerment, and femininity, influenced by perspectives from peer support, social constructionism, and feminism (83). Participants engaged in leisure activities, reflective exercises, and discussions on wellbeing, ABI, and feminine experiences. Field notes highlighted the processes that helped to construct a competent identity, experiences that helped with identity threat (e.g., normalization), empowerment and the importance of encouraging positive and feminine social identities.

ID (*k* = 1): Narrative Workshops for a LGBT Support Group. We identified one direct identity-focused intervention for individuals with ID (84). Participants, members of a LGBT support group, attended four narrative identity workshops where they created positive and alternative stories about the group identity and themselves using creative exercises and visual aids (e.g., photos, drawings, certificates). The authors noted participants felt safe and that the stories after the intervention were different from the initial stories, for example because they included new and positive self-aspects. However, the study lacked a clear explanation of the methodological steps used to reach this conclusion.

#### 3.6.6 Other indirect therapeutic or peer support interventions (*k* = 7)

SMI (*k* = 3): Long-term Therapy and Multicomponent Therapeutic Programs. One single-arm study reported reduced engulfment scores following long-term (1.5 year) individual CBT, which included self-appraisal training (making a daily list of positive events and a list of associated positive personal characteristics) and other elements (85). No significant self-concept changes (measured with the TSCS) were reported following a multi-component holistic hospital program (86). After a program for individuals transitioning to community care, identity emerged as a theme in post-intervention interviews, but no significant quantitative changes in engulfment were found (87).

ABI (*k* = 4): Peer Support, Multifamily Therapy and a valued living program. Two qualitative studies compared stories about identity before and after the intervention, showing a change from identity loss to a feeling of an adjusted, positive and socially connected identity after peer support interventions focused on adjustment after brain injury (88, 89). Another study assessed changes across multiple self-concept domains after multifamily therapy using the TSCS, but found no significant changes in participants with brain injury compared to a matched non-clinical control group (90). Lastly, one study quantitatively evaluated identity effects of a group-based valued-living intervention (ValiANT) with the HISDS (91). Although no significant interaction effect was found, the authors reported a significant overall time effect and a significantly greater proportion of participants in the treatment group, compared to the control group, who showed reliable identity change.

ID (*k* = 0). No indirect ID interventions fitted in this category.

### 3.7 Barriers, facilitators and recommendations

**Supplementary File S5** describes the recommendations for future studies made by the researchers of each study in the discussion-sections, and facilitators and barriers found in the results or discussion sections. By screening the results- and discussion sections of the included articles we identified all fragments in results or discussion sections in which the researchers or participants linked facilitating or hindering factors and intervention effects. Here we describe the most frequently mentioned facilitators and barriers across all studies.

Many of the barriers were related to participant characteristics (*k* = 13), such as participants’ intervention-readiness, willingness to share stories with others, disruptive or dominant group members, cognitive disabilities hindering intervention understanding and uptake, and factors related to illness severity. Negative emotions were also commonly mentioned: some interventions triggered anxiety, and several participants reported reliving negative emotions when discussing past experiences (*k* = 8). However, the authors differed in how they described these emotions, as some aimed to reduce negative emotional responses, whereas others framed them as opportunities for a discussion or emotional processing.

Factors that facilitated change and reflection were most often related to peer interactions in the group interventions (*k* = 13). This finding was also backed up by observations and participant feedback from numerous qualitative studies, which often reported how peer interactions fostered a sense of connection, alleviated feelings of isolation, normalized experiences, facilitated self-reflection through comparison of experiences, helped with establishing a positive atmosphere, and enabled participants to take up meaningful and competent roles as they could help the other participants (beneficence). Though the group format was often mentioned as a facilitator of beneficial social processes, many studies also recognized the value of individual interventions, and some explicitly reflected on the value of an individualized and more tailored format or of adding individual sessions to group interventions (*k* = 7). The second most frequently mentioned facilitator was the presence of positive emotion, for example when participants discussed their strengths or engaged in humorous exchanges (*k* = 6). Many studies also mentioned the role of the professional, such as the specific support offered or the therapeutic relationship (*k* = 5). Authors in general often attributed possible positive identity changes to general characteristics of the intervention (e.g. the time and attention devoted to personal narratives and positive self-aspects), or to experiences of doing an activity that is beneficial for one’s general health and wellbeing (e.g., doing new activities, making decisions, goal setting, or physical activity).

There was insufficient qualitative SMI data to compare recurring facilitators and barriers with those in other groups. However, when looking at all fragments related to facilitators, barriers and recommendations in the SMI studies, we observed some common structural aspects. These aspects were briefly mentioned in multiple SMI studies, but there was no consensus about the direction future research should take regarding these aspects: researchers most often reflected on the impact of participant characteristics (e.g., cognitive challenges), the format and delivery of the interventions (e.g., individual vs group setting, activity based, inclusion of peer support workers, type of support and reinforcement given by the therapist), the duration and amount of repetition needed to sustain emotional, cognitive and behavioral changes, and on the impact and emotional distress associated with certain activities (e.g., anxiety, increased self-awareness of disability, discussion of emotional topics).

## 4 Discussion

This review aimed to provide an overview of the aims, content, intervention outcomes, user experiences, and theoretical assumptions associated with interventions targeting identity that are designed for individuals with SMI, ABI or ID, and to explore how these findings can inform treatment for people with SMI. In total, 35 studies were identified, of which ten were SMI studies with a direct focus on identity and six were SMI studies indirectly targeting identity. Studies revealed a substantial heterogeneity regarding the intervention approaches, intervention context, participant characteristics, outcome measures, study methodology and identity focus. The therapeutic approaches that were most clearly represented included cognitive behavioral therapy and narrative therapy, though these were mentioned in less than half of the studies.

### 4.1 Intervention approaches

Approximately half of the included studies consistently and explicitly addressed identity throughout the intervention. These direct interventions focused on (social) identity, engulfment or narratives, often through therapy or peer support. A few direct studies focused on project-based interventions with creative output (e.g., advocacy presentation, photovoice, songwriting) or used a specific tool (e.g., activity tool), exercise (e.g., strengths exercise, self-photography), or material (e.g., bibliotherapy). Overall, approaches varied in participant involvement and structure (peer-led vs therapist led; structured vs informal), and amount of activity components and discussion components. Some studies mainly targeted intra-individual processes (e.g. a focus on the individual self-aspects, within in individual or group interventions), whereas others emphasized inter-individual meaning making (e.g. a focus on shared meanings or group identity). A few studies focused on how individuals saw themselves and were seen in a community context (e.g. making sense of experiences through inclusion in community settings, self-advocacy within a broader community, or group memberships). Topics of sessions varied widely, but recurring identity topics were related to positive identity (stigma, self-esteem, strengths), narrative identity, future and past identity, multifaceted self-aspects, social identity and interpersonal skills, values, feelings, embodied self, and coping with or adjusting to illness.

Studies frequently employed multiple overlapping identity-related aims. The most common conceptualizations of identity involved shifts from negative and stigmatized identity to positive identity, from illness-identity to self-complexity, from fragmented and problem-focused narrative identity to integrated continuous narratives, from identity theft to adjustment, and from compromised or stigmatized social identity to an empowered, included, and positive identity as a person with a disability. The analysis of identity conceptualizations was conducted by examining all groups combined rather than individually. While all themes were represented to some extent within each target group, some themes became more pronounced when considering the data collectively.

### 4.2 Key outcomes of interventions

While the majority of qualitative studies focused on individuals with ABI, few SMI studies used structured qualitative evaluations. Across different target groups, a key finding deriving from these qualitative studies was the shared social identities that emerged in various interventions, emphasizing the role of social connection and peer support in shaping identity. Interaction and social connection, especially in peer group interventions, fostered helping behaviors, sharing experiences, normalization, humor, upward/downward comparison, recognizing shared struggles, forming a collective identity, feeling connected, and developing new relationships. Qualitative studies also showed that identity change is a broad concept, varying across studies and participants. Qualitative studies often highlighted identity changes manifested through shifts in self-reflection, narratives, activities, self-esteem, and shifts in perspectives on illness, the future, and the self in relation to others.

Statistically significant changes in quantitative identity evaluations were reported in multiple studies in each target group and across individuals with different levels of functioning. However, the extent to which the reported identity changes in the included studies in this review can be attributed to specific interventions remains unclear, due to the methodological limitations of the studies. More research is needed to discern patterns regarding potential differences in outcomes of studies with different intervention ingredients, participant/facilitator characteristics, durations, and approaches.

### 4.3 Lessons learned from the different target groups

SMI interventions included elements such as participating in new activities, sharing experiences with supportive others, experiencing observable positive changes of one’s actions (e.g., after goal setting, decision making in the ward, physical exercise), and reflecting on identity through homework exercises, creative exercises, and discussion. One prominent topic in the SMI studies was the shift from illness-identity to a more positive, multifaceted self-concept. This often involved reducing illness-engulfment, where participants learned to see themselves as more than their diagnosis (57). SMI studies relied largely on quantitative evaluations, with approximately half of these showing at least some preliminary support for potential changes in identity – but as many studies were not controlled the role of the interventions remains unclear. From the controlled interventions, two SMI studies reported significantly better identity outcomes for the intervention group compared with the control group (57, 79) while one study found worse outcomes for a self-experiential intervention compared with a social-interaction control group (68). Many studies reported positive participant responses, for example after rather structured identity focused therapies, photovoice, using an activity tool and an intervention tailored to individuals transferring from first episode psychosis care to the community (41, 63, 77–79, 87) – but only two studies used qualitative methodologies. Notably, much is yet unknown regarding which identity aspects do or do not change and why, whether changes generalize to other contexts, and whether achieved changes can be attributed to the interventions. Due to the low number of (comparable) studies, it is impossible to integrate the findings. There are many gaps in the SMI literature and identity interventions for individuals with SMI have not been sufficiently studied to support policy recommendations. Moreover, many specific target groups (e.g., minority groups, individuals with bipolar disorder, or elderly people), many therapeutic orientations/backgrounds (e.g., therapists with a background in music therapy, spiritual care, psychodynamic therapy), and identity theories (e.g. social identity theory (16) remain largely unexplored in the SMI studies we identified.

The inclusion of ABI and ID studies (despite only identifying two ID studies that met the inclusion criteria and both with weak evidence) in this review highlighted several approaches that were not commonly found in SMI interventions, such as bibliotherapy, painting a mural, song-writing, leisure activities, combining individual and group sessions in one intervention, multifamily therapy, an intervention focused on valued living, practicing storytelling and presenting, and groups for individuals with a specific gender or sexual orientation. Furthermore, some of the ABI and ID studies show examples of strategies specifically tailored to individuals with cognitive disabilities, such as the use of visuals, summaries, creative activities, or using individual sessions to prepare for group discussions. Lastly, the ABI/ID studies more often employed qualitative evaluations than the SMI studies, enabling a deeper understanding of intra-individual processes (e.g., shifts in thinking and feeling about self) and inter-individual processes (e.g., social comparison and identification).

### 4.4 Facilitating identity change: directions for further exploration

Although identity intervention research is still in its early stages, with limited high-quality evidence, this review suggests potential avenues for designing interventions that require further testing. Here we discuss some promising key ingredients based on common approaches across different target groups.

#### 4.4.1 Making interventions accessible for individuals with cognitive challenges

One aim of this review was to explore intervention strategies for individuals with cognitive challenges, as identity can be a difficult and abstract concept, especially for those with communication barriers (41, 78). While the review shows examples of interventions developed for individuals with varying degrees of illness severity – most of which seemed feasible -, few SMI studies explicitly addressed the feasibility of these studies for individuals experiencing cognitive or communication difficulties. Approaches described in this review that are well-suited for these individuals include non-verbal and activity-based learning (e.g., sports, music), creative and visual learning (e.g., drawings, worksheets, passive visual aids), and group interventions with other people with similar impairments (including extra support persons if needed). These align with guidelines for individuals with ID, emphasizing simple communication (e.g., visuals), repetition, structure and space for individual guidance, and experience-near exercises (e.g. learning through doing exercises, roleplay, games or experiences – instead of mainly talking and giving information) (92). Other principles from ID guidelines were highlighted less frequently in the identity-literature, such as the use of intake assessments to decide if the intervention fits with the diagnostic profile, ability or motivation of the participant, explicit attention to establishing a safe and positive environment, and involvement of someone who is familiar to the participant (e.g., family or support person). Studies that align best with the ID guidelines were the identity activity tool for people with SMI (41), a women’s self-help group for ABI (83), and narrative workshops for individuals with ID (84).

#### 4.4.2 Learning through experience and meaningful engagement

Many studies described how positive experience-near activities and active engagement during an intervention may have an impact on identity. Arts-based activities, such as mural painting, photovoice and songwriting, may enable participants to access and share feelings and memories, and were received well by the participants (55, 58, 59, 63, 66). Music, drawing, and photography, made interventions more accessible, helped participants express identity, served as memory aids, or were enjoyable social activities that fostered connection and enabled participants to rediscover hobbies (41, 83, 84, 86). These methods also fit with the increased policy recommendations and research evidence for wellbeing effects of (community) arts in the broad health care field and in care for individuals with (severe) mental illnesses (93–97). Other types of activities and experiences were also hypothesized to impact identity, particularly when they evoked a sense of accomplishment or lead participants to see themselves in a new light. Examples mentioned in the studies included meditation (72, 78, 85, 86), self-advocacy or presenting something personal to others (59, 63–66, 84), watching videos or photographs of oneself (67, 68, 76), physical exercise (74, 75, 86), doing (leisure) activities (41, 83), and goal setting or making concrete efforts to implement (social) changes (70, 71, 80, 87, 91). These different activities may have the potential to help individuals gain new (social) identities or discover new healthy or meaningful activities – but more studies are needed to understand which activities are beneficial for which individuals and under which circumstances these have the potential to alter (social) identity. Furthermore, many studies highlighted the importance of experiencing the self in a positive context—for example, through receiving positive feedback, taking on roles that foster agency and autonomy, engaging in mental health self-advocacy, becoming more active, experiencing the (health) benefits of trying new things, contributing to a cause, or supporting fellow participants. Such experiences may enable participants to rediscover autonomy, competence, social connection and beneficence (feeling that one can contribute to others´ wellbeing) - factors linked to meaning in life (98).

#### 4.4.3 Learning through psycho-education, reflection, and sharing stories

Many talking therapies used psycho-education (63, 77, 79, 80, 86, 88, 90, 91) to inform participants about identity or illness. Talking therapies also used discussion and reflection to change negative or unhelpful thoughts. For example, multiple studies used exercises, informed by Cognitive Behavioral Therapy or Acceptance and Commitment Therapy, to focus on individual characteristics or values (70, 78, 79, 91). Seven interventions used cognitive-behavioral therapy principles or principles from Acceptance and Commitment Therapy. However, their evaluations were mainly quantitative, limiting our understanding of the working mechanisms and the role of cognitive restructuring or acceptance. However, the most frequently cited theoretical framework across the studies was narrative therapy, which was often examined qualitatively, providing clearer connections to mechanisms of identity change. Narrative interventions aimed to help participants create continuous, alternative stories about identity and illness, supporting participants in structuring their life stories, externalizing conversations, or developing “thick” narratives focused on positive aspects of identity (58, 59, 64, 78, 82, 84). In some studies, participants gave presented their life story to others (59, 63–65), which may foster feelings of accomplishment, whilst also having the potential to reduce public stigma. Sharing stories with others, referred to as ‘outsider witnessing’ in narrative therapy, may enable participants to externalize conversations and develop thick narratives, thus aiding (relational) identity development (84, 99). This focus on narratives may be particularly relevant for individuals with schizophrenia, whose life stories may lack coherence, may be detached from their identity, and often revolve around suffering (2). The current review did not include N = 1 anthropological or observational approaches, but these may provide additional insights about ways in which practitioners and their patients co-construct life stories (100). Despite limited evidence regarding identity outcomes after narrative interventions for individuals with SMI (only one study (78):), promising narrative approaches for individuals with SMI are available and merit further exploration: for example, through photovoice (63), life story work (101), narrative enhancement (102), and spiritual care (100).

#### 4.4.4 Enabling social connection and social identification

One of the main findings of this review is that many themes and recommendations from the studies were related to social connection. Researchers and therapists with different theoretical backgrounds hypothesized that relational- and group processes were an important facilitator of identity change, and this was also reflected in participant feedback. In all of the qualitative studies some relational (sub)themes were present, often related to peer support but also to the therapeutic alliance. Participants valued sharing knowledge, helping each other, and recognizing common (illness-related) experiences (64, 82, 83, 87, 88). Meetings with peers also helped to change identity views, for example because they offered an opportunity to reflect (with humor) about difficulties, made participants see themselves in terms of positive shared identities (e.g., as friends, shared hobby, shared gender), feel understood, and feel more hopeful or agentic after comparing themselves with others (64, 69, 82, 83, 88, 89). Notably, the evidence for common qualitative relational themes primarily comes from ABI studies, as only two SMI studies included extensive qualitative analyses (41, 87). Nevertheless, the relational findings are consistent with another recent review, which identified connectedness as a key aspect of community arts - facilitating identity recovery in individuals with SMI through feeling accepted and validated by others (94).

Based on the SIAH (16), our findings suggest that many interventions implicitly target social identity processes that are relevant for well-being. According to the group circumstance hypothesis, reframing a stigmatized illness identity into a more empowering one may help reduce the negative impact of compromised identities. Likewise, several interventions included elements—such as engaging in meaningful activities, reflecting on multiple identities, strengthening social connections, and integrating illness into one’s broader self-concept—that may support the development of positive and compatible social identities, another key SIAH principle. This aligns with the Cognitive-Developmental Model of Identity Integration (103), which proposes that individuals adjust to new identities through cognitive processing, emotional acceptance, and ultimately the integration of old and new identities into a coherent whole. Only one study explicitly applied SIAH principles by mapping social networks and supporting participants to maintain or gain group memberships, showing promising effects on identity integration and in-group identification (80). However, formal evaluation of the underlying identity mechanisms remains limited and warrants further investigation.

Although in the current review most evidence regarding the relational identity processes comes from qualitative (ABI) studies of group interventions, there is no consensus that group interventions are necessary. Social identity development may also be supported through one-on-one interventions during which social self-concept is a topic (55, 58, 80), or in one-on-one interventions with a focus on reciprocity and equality (41). Studies combining individual and group sessions report different advantages: peer contact can help people to connect with others and re-evaluate illness, whereas individual support feels safe, enables in depth discussion of (social) identity and can be more practical (e.g., helping to get active in a community setting) (82, 87). Furthermore, one social identity intervention compared group and individual delivery formats and found no significant differences between them, although in both conditions some significant improvements were found (80). Thus, group and individual interventions both may influence individual- and social identity – potentially through different mechanisms.

### 4.5 Limitations and considerations for future studies

#### 4.5.1 Measuring identity change

One of the primary challenges identified in this review was the difficulty in measuring identity change. This measurement must capture the complexity of the construct, facilitate comparisons across studies, have a clear link with expected intervention mechanisms and identity theories employed in the intervention, and account for individual variations in what constitutes meaningful identity change for each participant.

Most quantitative studies used self-report scales, but for many of those scales, there is a lack of knowledge regarding the psychometric properties, which may be a problem especially as some individuals may not understand all questions (66, 80, 104). In studies that used multiple different (qualitative or quantitative) identity outcomes, these outcomes did not show the same tendencies (57, 58, 74, 87). Although this may be due to a lack of appropriate power or to ceiling effects, it is also possible that interventions influence one facet of identity and not another. For example, some studies showed significant results regarding how strongly the self was defined by illness, but not regarding self-esteem, (self-) stigma, and self-concept (57, 79), even though these concepts are generally linked in engulfment theory (56, 57). One interpretation is that, although individuals may have learned that they are more than their illness, they have not fully incorporated this idea to the point where they truly feel a positive identity, or that they still experience stigma and discriminatory behavior imposed upon them by others. Studies with appropriate power, including a control group and using multiple identity outcome measures are needed to replicate and understand these findings. Qualitative interviews or observations can be valuable to understand why scales measuring related concepts may show different tendencies. However, it is also possible that some of these findings reflect the sensitivity of the measures. For example, to our knowledge, the reliability of the TSCS has not been tested in the target groups included in the current review.

Qualitative evaluations allow for a contextualized analysis, which can help to understand identity change in the context of the intervention, and in the context of specific goals or struggles of individual participants – whilst focusing on the identity domains that are most meaningful for the participants. However, qualitative results are not aimed to be generalizable, and differences in interpretations of identity in qualitative studies can make it difficult to draw conclusions about identity effects of interventions. For example, the two qualitative SMI studies included in this review showed different interpretations of identity (learning new things/recalling memories vs valued self-perception) (41, 87). Both studies also mentioned relational dynamics, but did not explicitly connect this to the theme ´identity´ - in contrast to many ABI studies that explicitly linked these themes.

One potential draw-back of qualitative interviews is noted by van der Meer et al. (2021), who observed that participants with SMI struggled to reflect on identity in the post-intervention interview (41). This is consistent with other studies showing that individuals with SMI find abstract identity-related topics challenging (28, 78, 80). Alternative qualitative approaches can be found in the ABI/ID studies included in this review, for example studies using field notes to observe identity changes, or studies comparing participants’ stories about their life or identity before and after an intervention (83, 84, 88, 89). In the wider literature other alternatives have also been described to assess (narrative) identity change, for example the use of informant stories or observation of non-verbal behavior during story telling (105). Another approach which does not draw heavily on verbal assessment is the analysis of creative artworks produced during interventions (see (106, 107):), but until now these methods have merely been used to describe art works produced during interventions, and not to evaluate (intervention-related) identity changes. Mixed methods can be valuable for comparing findings, as qualitative data can provide deeper insights into the reasons behind quantitative changes (or the lack thereof), while quantitative data can help contextualize qualitative results—particularly when these are based on third-person reports or when participants have difficulty reflecting on their experiences. A second concern is the quality and methodological rigor of the qualitative analysis. Some researchers used leading questions, failed to describe the data-analysis process transparently, or placed less emphasis on critical participant observations compared to positive ones. To avoid bias towards selecting participants’ or researcher’s viewpoints, studies may benefit from additional feedback rounds, to see if there is agreement in the whole participant group regarding certain outcomes (akin to iterative approaches such as Delphi studies and triangulation methods), from the use of researchers with different perspectives, or from identity tools that allow for qualitative input and quantification (e.g (108, 109). Social identity mapping (108) was used in one study (80), which noted that future quantitative applications would benefit from pre-defining expected changes in social identity variables, as small effects and numerous variables prevented analysis in this study.

#### 4.5.2 Contextual factors: values and beliefs shaped by the intervention or context

Self-understanding and personal values do not exist in a vacuum; they are context-dependent. Because interventions create contexts that can influence a person’s sense of self, it is essential for researchers and therapists to remain reflexive in their work – for example regarding the underlying values of the intervention or the practitioner. Culturally important values, along with assumptions about what constitutes a “healthy identity” or “positive recovery,” may be implicitly embedded by the therapist or conveyed through the intervention itself. While reviewing the studies, we observed differences in intervention approaches, particularly regarding recovery-oriented versus deficit-focused paradigms; but these differences were difficult to pinpoint as they were often not clearly articulated. In the current review it was therefore not possible to compare outcomes of interventions with different underlying values or paradigms. Although some studies mentioned the values underpinning their approaches—such as Gelech et al. (83), which utilized a feminist model - most studies did not reflect on the personal, therapeutic, or cultural values integrated into the interventions. In the context of identity, it is crucial to examine these assumptions and values embedded within interventions, as these can significantly influence how identity evolves. For instance, a focus on deficits may lead to negative changes in identity (68). Values incorporated by the therapist or the intervention may play an important role in identity change; however, these values can be undermined by the attitudes of others within the social context in which participants are situated. For example, Lukoff et al. (86) note that the holistic principles from the intervention clashed with values and approaches in the institution – undermining the intervention. According to social identity approaches (e.g. the SIAH and SIMIC), social identification with others can be beneficial for wellbeing, but only if the new identity is compatible with other social identities (16, 17). Further (interdisciplinary) research is necessary to determine efficacy and reception of interventions for individuals who perceive important differences between their own identity and values and the identity (or values, experiences, characteristics) of other participants (see also (69, 84)). In this context it is notable that none of the SMI studies explicitly focused on individuals from a double minority group, who may feel excluded from both the illness community and other minority communities (110).

#### 4.5.3 Strengths and limitations of the current review

A major strength of the current review is the comprehensive approach, encompassing a transdiagnostic focus, rigorous search, and thorough analysis of both quantitative and qualitative information regarding the contextual factors, mechanisms and outcomes. Studies included a wealth of information – for example regarding the intervention approaches, common observations between researchers, and the participant feedback – which together result in numerous recommendations for further research. Nevertheless, studies were highly heterogeneous and the quality appraisal showed that the majority of the quantitative studies had major study limitations. Qualitative studies were useful to develop hypotheses about working mechanisms - but it should be noted that these studies were highly variable (regarding the aims, operationalization of identity, and contexts) and that few SMI and ID studies used formal qualitative evaluations. Given the large differences in approaches and outcomes and numerous quantitative studies lacking significant results, it is recommended that future studies also evaluate barriers, adverse effects and suggestions for program improvement. These studies should include an explanation of how the researchers tried to avoid confirmation bias and socially desirable responding, as this was not extensively discussed in most of the existing literature.

The formulation and application of inclusion criteria can greatly influence the conclusions of systematic reviews. To minimize bias in our review, we implemented several measures: dual screening by at least two reviewers, regular team discussions, pre-registration of an *a priori* protocol (49), and a detailed guide with definitions and examples for inclusion and exclusion. Despite these efforts, challenges emerged during screening due to the complexity of identity as a multifaceted concept, which lacks consensus regarding the definition and operationalization. This was evident in the included studies, where terms like identity, self-esteem, and sense of self were often overlapping or conflated. It was also evident in qualitative studies, which grouped a wide array of terms under the umbrella category ‘identity’, or ‘sense of self’. Overall, many studies mentioned identity or identity-related processes, but it was sometimes difficult to determine whether the study explicitly targeted identity or if this was a pre-specified aim, even when extensive decision rules were applied. Our own conceptualizations of identity may also have influenced this process. For example, the current inclusion criteria may have favored studies with relatively explicit and clear definitions of identity, while studies with more vague or implicit operationalizations—such as psychodynamic or art-based interventions—may have been excluded. By taking a broad approach with our search terms, we aimed to minimize the influence of our personal biases, but it has also forced us to make decisions that are partly subjective. For transparency, we composed a list with examples of relevant excluded studies and studies that were difficult to decide (**Supplementary File 2**).

We want to highlight that the results from this review are also limited by the specific sociocultural context in which the current review and the included intervention studies took place. Many therapeutic backgrounds were not represented in the current review (e.g., spiritual care interventions or psychodynamic approaches). The current review solely includes studies conducted in Western-oriented countries and predominantly reflect findings from young adults and midlife adults (ages 18 to retirement). Additionally, certain diagnoses, such as bipolar disorder, were underrepresented, and no interventions specifically targeted individuals with SMI who faced dual stigma, such as those from the LHBTQIA+ community or cultural minorities.

#### 4.5.4 Recommendations for future studies

This review highlighted different interconnected types of identity change: feeling or thinking (more) positively about the self, moving away from stigmatized views and negative illness identities, exploring multifaceted identity (through new positive roles or reflection on self-aspects), understanding the self in the context of an integrated and continuous life story, adjusting to life with illness, and experiencing empowerment, inclusion and social connection. Future studies could investigate whether the identity focus of an intervention matters, or whether a particular identity focus is especially suited in a specific context or for certain participants. Multiple mechanisms may facilitate these identity changes, such as positive experiences, social connectedness, rediscovery of meaningful activities, narrative or cognitive re-construction, self-reflection, and more. In the wider literature there seems to be most support for mechanisms related to meaning making, agency (e.g., autonomy, feeling of achievement, competence, feeling of control over illness) and social connection or communion (e.g., beneficence, feeling connected, included and supported and social identity) (c.f (11, 16, 94, 98)). These mechanisms are central to theories relevant for identity research, such as theories about meaning in life (98), social identity (16), and narrative identity (11). The role of agency and social connection is also highlighted in a realist review about community arts and SMI, which identified two mechanism pathways that influenced identity change: ‘feeling in control of illness through coping’ and ‘achieving acceptance through connectedness’ (94). We expect that these influence the positivity of self-feelings and support meaning making and activation. A wide range of intervention elements have been hypothesized to enable these mechanisms, including direct identity-focused elements (e.g., explicitly discussing identity-topics), and less specific approaches such as creative or activity-based projects, group- and individual support, and strategies based on narrative- or CBT principles. Future research is needed to study these interventions in the context of specific target groups (such as individuals who experience communication or cognitive difficulties), to systematically analyze facilitators and mechanisms of change, and to understand the role of relational and social processes related to these mechanisms.

In the current review, interventions could only be included if they either clearly aimed to target identity or aimed to measure identity. This resulted in including some studies which did not explicitly or clearly targeted identity even though identity was measured, whereas other studies which are strongly focused on identity related processes were excluded because identity was not evaluated. This means that studies that do not explicitly focus on identity were not included. Examples are interventions such as Narrative Enhancement and Cognitive Therapy (102) or Metacognitive Insight and Reflection Therapy (111), which have been used repeatedly in individuals with SMI. However, interventions with a totally different focus or approach might also be relevant, as the current review showed that interventions not explicitly focusing on identity may also bring about relevant changes in identity nevertheless. **Supplementary File S2** provides examples of the breadth of approaches that may be relevant and could inspire future theoretical and empirical developments.

The current review also explored barriers and facilitators of intervention efficacy and uptake, as well as recommendations for future studies provided by the study authors and study participants. In line with the diversity of the included studies, the analysis of barriers, facilitators and recommendations highlighted a wealth of diverse hypotheses. Common reflections which recurred across studies were related to the participant characteristics, emotional impact of the interventions, group versus individual formats, role of peers and professionals, intervention characteristics and intervention activities – but more studies are needed to understand the role of these aspects better. Peer interactions and relational processes were most frequently identified as key facilitators of (identity) change, which is in line with the SIAH (16). However, the current review did not find direct evidence that peer-based interventions are superior to individual formats, with some studies using mixed formats reporting benefits for both approaches. Furthermore, many studies suggested that longer intervention duration might be beneficial, but this was not formally tested. The current review also found no consistent patterns linking intervention length to differences in identity-related outcomes. Intervention developers can use **Supplementary File S5** to explore relevant hypotheses and mechanisms in relation to the participant group and type of intervention they want to develop.

Due to the heterogeneous nature of intervention strategies and outcome measures, as well as the low number of (randomized) controlled trials, a follow-up meta-analysis is not appropriate. Other review studies are needed to summarize the current evidence regarding (*post-hoc*, qualitative) findings of identity change after specific interventions -such as peer-support, arts-based interventions, interventions with a focus on community meaning-making, narrative interventions, self-advocacy, activity-oriented interventions and interventions focusing on stigma, self-esteem, personal recovery or social connection. A specific identity-oriented framework, for example rooted in recovery, narrative, or social identity theories, may be necessary to guide or limit these reviews. This is particularly important because a wide range of experiences can be categorized as identity-related, and employing more precise identity-change hypotheses can help to focus the review and to identify more specific gaps in the literature. As identity intervention research is still in an early phase, it is relevant to focus not only on intervention outcomes, but also on intervention mechanisms, for example through a realist review (c.f (94)).

### 4.6 Conclusion

The 35 studies included in this review showed considerable variation regarding intervention approaches, study context and outcomes. Many studies aimed to support individuals in fostering a more positive self-view, developing complex identities, adjusting to illness, reconstructing integrated self-narratives, and cultivating an empowered (social) identity. Participants engaged in learning through experience, social connections, self-reflection, and narrative- or cognitive restructuring. The current review highlights many hypotheses which may inform future studies. We highlighted promising elements of interventions, but more research is needed; especially regarding the mechanisms of identity change and underlying values of interventions. Overall, scientific studies about identity interventions in individuals with SMI, ABI and ID are scarce, especially studies with a clear identity focus which also evaluate identity outcomes; and most studies have methodological issues limiting generalizability. Future (interdisciplinary) research can focus on the development and evaluation of (established and new) identity-sensitive interventions, and should incorporate strategies to test mechanisms through which interventions can influence identity.

## Footnotes

1 Note that some of these direct interventions focused on one identity-related aspect, such as self-esteem or strengths, instead of identity as a whole. These studies were included because they evaluated identity outcomes.

## References

[1] Conneely M McNamee P Gupta V Richardson J Priebe S Jones JM . Understanding identity changes in psychosis: A systematic review and narrative synthesis. Schizophr Bull. (2020) 47:309–22. doi: 10.1093/schbul/sbaa124, PMID: 32989443 PMC7965068

[2] Cowan HR Mittal VA McAdams DP . Narrative identity in the psychosis spectrum: A systematic review and developmental model. Clin Psychol Rev. (2021) 88:102067. doi: 10.1016/j.cpr.2021.102067, PMID: 34274799 PMC8376804

[3] Kaite CP Karanikola M Merkouris A Papathanassoglou EDE . An ongoing struggle with the self and illness”: A meta-synthesis of the studies of the lived experience of severe mental illness. Arch Psychiatr Nurs. (2015) 29:458–73. doi: 10.1016/j.apnu.2015.06.012, PMID: 26577563

[4] Yanos PT DeLuca JS Roe D Lysaker PH . The impact of illness identity on recovery from severe mental illness: A review of the evidence. Psychiatry Res. (2020) 288. doi: 10.1016/j.psychres.2020.112950, PMID: 32361335

[5] Brinthaupt TM . Identity. In: Darity WA Jr , editor. International encyclopedia of the social sciences, 2nd ed. Macmillan Reference USA, Detroit, MI (2008). p. 551–5.

[6] Hoy MB . Self-concept. In: Darity WA Jr , editor. International Encyclopedia of the Social Sciences. 7. 2nd ed. Detroit, MI: Macmillan Reference USA (2008). 398–400.

[7] Oyserman D Elmore K Smith G . Self, self-concept, and identity. In: Leary MR Tangney JP , editors. Handbook of self and identity, 2ed, vol. New York: The Guilford Press (2012). p. 69–104.

[8] Owens TJ Robinson DT Smith-Lovin L . Three faces of identity. Annu Rev Sociol. (2010) 36:477–99. doi: 10.1146/annurev.soc.34.040507.134725

[9] James W . The principles of psychology: H. New York: Holt and Company (1890).

[10] Nelson B Raballo A . Basic self-disturbance in the schizophrenia spectrum: taking stock and moving forward. Psychopathology. (2015) 48:301–9. doi: 10.1159/000437211, PMID: 26368118

[11] McAdams DP . The stories we live by: Personal myths and the making of the self. New York, NY, US: William Morrow & Co (1993) p. 336–.

[12] McLean KC Pasupathi M Pals JL . Selves creating stories creating selves: A process model of self-development. Pers Soc Psychol Review. (2007) 11:262–78. doi: 10.1177/1088868307301034, PMID: 18453464

[13] Hermans HJM . The dialogical self: Toward a theory of personal and cultural positioning. Cult Psychol. (2001) 7:243–81. doi: 10.1177/1354067X0173001

[14] Ownsworth T . Self-identity after brain injury. London, New York: Psychology Press (2014).

[15] Meyerson DE Zuckerman S . Identity theft: rediscovering ourselves after stroke. 2 ed. Kansas City, Missouri: Andrews McMeel Publishing (2019).

[16] Haslam C Jetten J Cruwys T Dingle GA Haslam SA . The new psychology of health. New York, NY: Routledge, Taylor & Francis Group. (2018).

[17] Jetten J Haslam S Iyer A Haslam C . Turning to others in times of change: social identity and coping with stress. In: Stürmer S Snyder M , editors. The psychology of prosocial behavior. Group Processes, Intergroup Relations, and Helping, Oxford, UK: Blackwell (2009). p. 139–56.

[18] Delespaul PH . Consensus over de definitie van mensen met een ernstige psychische aandoening (EPA) en hun aantal in Nederland. Tijdschr Psychiatr. (2013) 55:427–38. 23864410

[19] Moreno-Küstner B Martín C Pastor L McKenna PJ . Prevalence of psychotic disorders and its association with methodological issues. A systematic review and meta-analyses. PLoS ONE. (2018) 13(4):e0195687. doi: 10.1371/journal.pone.0195687, PMID: 29649252 PMC5896987

[20] Killaspy H . The ongoing need for local services for people with complex mental health problems. Psychiatr Bull. (2018) 38:257–9. doi: 10.1192/pb.bp.114.048470, PMID: 25505623 PMC4248159

[21] Berardelli I Sarubbi S Rogante E Hawkins M Cocco G Erbuto D . The role of demoralization and hopelessness in suicide risk in schizophrenia: A review of the literature. Medicina. (2019) 55(5):200. doi: 10.3390/medicina55050200, PMID: 31126145 PMC6571661

[22] Morgan VA McGrath JJ Jablensky A Badcock JC Waterreus A Bush R . Psychosis prevalence and physical, metabolic and cognitive co-morbidity: data from the second Australian national survey of psychosis. Psychol Med. (2014) 44:2163–76. doi: 10.1017/S0033291713002973, PMID: 24365456 PMC4045165

[23] Morgan VA Waterreus A Carr V Castle D Cohen M Harvey C . Responding to challenges for people with psychotic illness: Updated evidence from the Survey of High Impact Psychosis. Aust New Z J Psychiatry. (2016) 51:124–40. doi: 10.1177/0004867416679738, PMID: 27913580

[24] Berna F Göritz AS Schröder J Martin B Cermolacce M Allé MC . Self-disorders in individuals with attenuated psychotic symptoms: Contribution of a dysfunction of autobiographical memory. Psychiatry Res. (2016) 239:333–41. doi: 10.1016/j.psychres.2016.03.029, PMID: 27058160

[25] Ricarte JJ Ros L Latorre JM Watkins E . Mapping autobiographical memory in schizophrenia: Clinical implications. Clin Psychol Rev. (2017) 51:96–108. doi: 10.1016/j.cpr.2016.11.004, PMID: 27846438

[26] van der Meer L de Vos AE Stiekema APM Pijnenborg GHM van Tol MJ Nolen WA . Insight in schizophrenia: Involvement of self-reflection networks? Schizophr Bull. (2012) 39:1288–95. doi: 10.1093/schbul/sbs122, PMID: 23104865 PMC3796073

[27] Lysaker PH Hamm JA Vohs J Kukla M Pattison ML Leonhardt BL . Understanding the course of self-disorders and alterations in self- experience in schizophrenia: Implications from research on metacognition. Curr Psychiatry Rev. (2018) 14:160–70. doi: 10.2174/1573400514666180816113159

[28] Muthert H . Bridging inner and outer worlds: A psychodynamic approach to meaningful mourning. In: Berger P Buitelaar M Knibbe K , editors. Religion as relation: studying religion in context. Sheffield: Equinox Publishing Ltd. (2021) p. 192–213.

[29] Thornicroft G Brohan E Rose D Sartorius N Leese M . Global pattern of experienced and anticipated discrimination against people with schizophrenia: a cross-sectional survey. Lancet. (2009) 373:408–15. doi: 10.1016/S0140-6736(08)61817-6, PMID: 19162314

[30] Park CL . Making sense of the meaning literature: an integrative review of meaning making and its effects on adjustment to stressful life events. Psychol Bull. (2010) 136:257–301. doi: 10.1037/a0018301, PMID: 20192563

[31] McCay EA Seeman MV . A scale to measure the impact of a schizophrenic illness on an individual’s self-concept. Arch Psychiatr Nurs. (1998) 12:41–9. doi: 10.1016/S0883-9417(98)80007-1, PMID: 9489173

[32] Jordan G Malla A Iyer SN . It’s brought me a lot closer to who i am”: A mixed methods study of posttraumatic growth and positive change following a first episode of psychosis. Front Psychiatry. (2019) 10. doi: 10.3389/fpsyt.2019.00480, PMID: 31379615 PMC6643164

[33] Anthony WA . Recovery from mental illness: The guiding vision of the mental health service system in the 1990s. Psychosoc Rehabil J. (1993) 16:11–23. doi: 10.1037/h0095655

[34] Deegan PE . Recovery: The lived experience of rehabilitation. Psychosocial Rehabil J. (1988) 11:11–9. doi: 10.1037/h0099565

[35] Bird V Leamy M Tew J Le Boutillier C Williams J Slade M . Fit for purpose? Validation of a conceptual framework for personal recovery with current mental health consumers. Aust New Z J Psychiatry. (2014) 48:644–53. doi: 10.1177/0004867413520046, PMID: 24413806

[36] Gwinner K Knox M Brough M . Making sense of mental illness as a full human experience: Perspective of illness and recovery held by people with a mental illness living in the community. Soc Work Ment Health. (2013) 11:99–117. doi: 10.1080/15332985.2012.717063

[37] Winsper C Crawford-Docherty A Weich S Fenton S-J Singh SP . How do recovery-oriented interventions contribute to personal mental health recovery? A systematic review and logic model. Clin Psychol Rev. (2020) 76:101815. doi: 10.1016/j.cpr.2020.101815, PMID: 32062302

[38] Leamy M Bird V Boutillier CL Williams J Slade M . Conceptual framework for personal recovery in mental health: systematic review and narrative synthesis. BJPsych. (2011) 199:445–52. doi: 10.1192/bjp.bp.110.083733, PMID: 22130746

[39] Palmer BW Dawes SE Heaton RK . What do we know about neuropsychological aspects of schizophrenia? Neuropsychol Rev. (2009) 19:365–84. doi: 10.1007/s11065-009-9109-y, PMID: 19639412 PMC2745531

[40] Morgan VA Leonard H Bourke J Jablensky A . Intellectual disability co-occurring with schizophrenia and other psychiatric illness: population-based study. BJPsych. (2018) 193:364–72. doi: 10.1192/bjp.bp.107.044461, PMID: 18978313

[41] van der Meer L Jonker T Wadman H Wunderink C van Weeghel J Pijnenborg GHM . Targeting personal recovery of people with complex mental health needs: The development of a psychosocial intervention through user-centered design. Front Psychiatry. (2021) 12. doi: 10.3389/fpsyt.2021.635514, PMID: 33897494 PMC8060492

[42] Cowan HR Lind M . Narrative identity disturbances in psychopathology: An ecologically valid transdiagnostic framework. J Psychopathol Clin Sci. (2024) 133:503–4. doi: 10.1037/abn0000932, PMID: 39101938

[43] American Psychiatric Association . Diagnostic and statistical manual of mental disorders. (2013) Vol. 5.

[44] Beart S Hardy G Buchan L . How people with intellectual disabilities view their social identity: A review of the literature. J Appl Res Intellect Disabil. (2005) 18:47–56. doi: 10.1111/j.1468-3148.2004.00218.x

[45] Villa D Causer H Riley GA . Experiences that challenge self-identity following traumatic brain injury: a meta-synthesis of qualitative research. Disabil Rehabil. (2020) 43:3298–314. doi: 10.1080/09638288.2020.1743773, PMID: 32223350

[46] Munn Z Peters MDJ Stern C Tufanaru C McArthur A Aromataris E . Systematic review or scoping review? Guidance for authors when choosing between a systematic or scoping review approach. BMC Med Res Methodol. (2018) 18. doi: 10.1186/s12874-018-0611-x, PMID: 30453902 PMC6245623

[47] Tricco AC Lillie E Zarin W O’Brien KK Colquhoun H Levac D . PRISMA extension for scoping reviews (PRISMA-ScR): Checklist and explanation. Ann Intern Med. (2018) 169:467–73. doi: 10.7326/M18-0850, PMID: 30178033

[48] Peters MDJ CG McInerney P Munn Z Tricco AC Khalil H . Chapter 11: scoping reviews. In: JBI manual for evidence synthesis (2020 version) (2020). doi: 10.46658/JBIMES-20-01

[49] Kronemeijer UE Muthert H Pijnenborg M van Setten ERH van der Meer L . Interventions targeting identity: a protocol for a transdiagnostic scoping review with implications for adults with a severe mental illness. (2022). doi: 10.17605/OSF.IO/U5FZT PMC12916650 41726837

[50] van Geestelijk VerZorger V . Professional standard spiritual caregiver (2015). Available online at: https://vgvz.nl/wp-content/uploads/2023/02/VGVZ_Professional_Standard_2015_Main_Text_EN_v03_WITH_APPENDICES.pdf (Accessed December 6, 2024).

[51] Ouzzani M Hammady H Fedorowicz Z Elmagarmid A . Rayyan—a web and mobile app for systematic reviews. Syst Rev. (2016) 5. doi: 10.1186/s13643-016-0384-4, PMID: 27919275 PMC5139140

[52] Arksey H O’Malley L . Scoping studies: towards a methodological framework. Int J Soc Res Methodol. (2005) 8:19–32. doi: 10.1080/1364557032000119616

[53] Hoffmann TC Glasziou PP Boutron I Milne R Perera R Moher D . Better reporting of interventions: Template for intervention description and replication (TIDieR) checklist and guide. BMJ. (2014) 348:g1687–g. doi: 10.1136/bmj.g1687, PMID: 24609605

[54] Hong Q Pluye P Fàbregues S Bartlett G Boardman F Cargo M . Mixed methods appraisal tool (MMAT), version 2018. Registration of copyright (1148552). Industry Canada: Canadian Intellectual Property Office (2018).

[55] Baker FA Rickard N Tamplin J Roddy C . Flow and meaningfulness as mechanisms of change in self-concept and well-being following a songwriting intervention for people in the early phase of neurorehabilitation. Front Hum Neurosci. (2015) 9. doi: 10.3389/fnhum.2015.00299, PMID: 26082702 PMC4443737

[56] McCay E Beanlands H Leszcz M Goering P Seeman MV Ryan K . A group intervention to promote healthy self-concepts and guide recovery in first episode schizophrenia: A pilot study. Psychiatr Rehabil J. (2006) 30:105–11. doi: 10.2975/30.2006.105.111, PMID: 17076053

[57] McCay E Beanlands H Zipursky R Roy P Leszcz M Landeen J . A randomised controlled trial of a group intervention to reduce engulfment and self-stigmatisation in first episode schizophrenia. Adv Ment Health. (2007) 6:212–20. doi: 10.5172/jamh.6.3.212

[58] Roddy C Rickard N Tamplin J Lee Y-EC Baker FA . Exploring self-concept, wellbeing and distress in therapeutic songwriting participants following acquired brain injury: A case series analysis. Neuropsychol Rehabil. (2018) 30:166–86. doi: 10.1080/09602011.2018.1448288, PMID: 29560784

[59] Strong KA Sather TW . It’s not often that people want to hear me talk about my life”: Storytelling experiences of people with aphasia in an interdisciplinary songwriting project. Int J Speech Lang Pathol. (2024) 26:737–49. doi: 10.1080/17549507.2023.2251724, PMID: 37807482

[60] Fitts WH Warren WL . Western Psychological S. Tennessee self-concept scale: TSCS-2. Manual. 2nd ed. Los Angeles, Calif: Western Psychological Services (1996).

[61] Roid GH Fitts WH . Tennessee self-concept scale (TSCS): revised manual. Los Angeles, Calif: Western Psychological Services (1988).

[62] Tyerman A Humphrey M . Changes in self-concept following severe head injury. Int J Rehabil Res. (1984) 7:11–23. doi: 10.1097/00004356-198403000-00002, PMID: 6735545

[63] Mizock L Russinova Z DeCastro S . Recovery narrative photovoice: Feasibility of a writing and photography intervention for serious mental illnesses. Psychiatr Rehabil J. (2015) 38:279–82. doi: 10.1037/prj0000111, PMID: 25603013

[64] Strong KA Lagerwey MD Shadden BB . More than a story: my life came back to life. Am J Speech Lang Pathol. (2018) 27:464–76. doi: 10.1044/2017_AJSLP-16-0167, PMID: 29497756

[65] Hoepner JK Yingst H Harder B Zehm C . I never thought I would be an international speaker … but I am”: An interpretive qualitative analysis of experiences of a project-based advocacy intervention. Neuropsychol Rehabil. (2022) 32:2077–101. doi: 10.1080/09602011.2022.2050410, PMID: 35297728

[66] Trzaska JD . The use of a group mural project to increase self-esteem in high-functioning, cognitively disabled adults. Arts Psychother. (2012) 39:436–42. doi: 10.1016/j.aip.2012.06.003

[67] Spire RH . Photographic self-image confrontation. Am J Nurs. (1973) 73:1207–10. doi: 10.2307/3422783, PMID: 4575620

[68] Muzekari LH Weinman B Kreiger PA . Self-experiential treatment in chronic schizophrenia. J Nerv Ment Dis. (1973) 157:420–7. doi: 10.1097/00005053-197312000-00003, PMID: 4758745

[69] Hoover E Bernstein-Ellis E Meyerson D . Using bibliotherapy to rebuild identity for people with aphasia: A book club experience. J Commun Disord. (2023) 105. doi: 10.1016/j.jcomdis.2023.106363, PMID: 37517172

[70] Andrewes HE Walker V O’Neill B . Exploring the use of positive psychology interventions in brain injury survivors with challenging behaviour. Brain Injury. (2014) 28:965–71. doi: 10.3109/02699052.2014.888764, PMID: 24826958

[71] Cerniglia RP Horenstein D Christensen EW . Group decision-making and self-management in the treatment of psychiatric patients. J Clin Psychol. (1978) 34:489–93. doi: 10.1002/1097-4679(197804)34:2<489::AID-JCLP2270340251>3.0.CO;2-V, PMID: 681529

[72] Randal C Bucci S Morera T Barrett M Pratt D . Mindfulness-based cognitive therapy for psychosis: measuring psychological change using repertory grids. Clin Psychol Psychother. (2015) 23:496–508. doi: 10.1002/cpp.1966, PMID: 26077540

[73] Hadas-Lidor N Katz N Tyano S Weizman A . Effectiveness of dynamic cognitive intervention in rehabilitation of clients with schizophrenia. Clin Rehabil. (2001) 15:349–59. doi: 10.1191/026921501678310153, PMID: 11518436

[74] Brinkmann JR Hoskins TA . Physical conditioning and altered self-concept in rehabilitated hemiplegic patients. Phys Ther. (1979) 59:859–65. doi: 10.1093/ptj/59.7.859, PMID: 450993

[75] Fines L Nichols D . An evaluation of a twelve week recreational kayak program: Effects on self-concept, leisure satisfaction and leisure attitude of adults with traumatic brain injuries. J Cognit Rehabil. (1994) 12:10–5. Available online at: https://psycnet.apa.org/record/1995-26433-001.

[76] Helffenstein DA Wechsler FS . The use of interpersonal process recall (IPR) in the remediation of interpersonal and communication skill deficits in the newly brain-injured. J Clin Neuropsychol. (1982) 4:139–42. Available online at: https://psycnet.apa.org/record/1983-06229-001.

[77] Zahniser JH Coursey RD . The self-concept group: Development and evaluation for use in psychosocial rehabilitation settings. Psychiatr Rehabil J. (1995) 19:59–64. doi: 10.1037/h0095439

[78] Sokol Y Ridley J Goodman M Landa Y Hernandez S Dixon L . Continuous identity cognitive therapy: Feasibility and acceptability of a novel intervention for suicidal symptoms. J Cognit Psychother. (2021) 35:64–80. doi: 10.1891/JCPSY-D-20-00023, PMID: 33397785

[79] Konsztowicz S Gelencser CR Otis C Schmitz N Lepage M . Self-concept and Engagement in LiFe (SELF): A waitlist-controlled pilot study of a novel psychological intervention to target illness engulfment in enduring schizophrenia and related psychoses. Schizophr Res. (2021) 228:567–74. doi: 10.1016/j.schres.2020.11.028, PMID: 33272766

[80] Hogg LI Smith LGE Haslam C Coxhill L Kurz T Hobden G . A randomised feasibility trial comparing group and individual format GROUPS FOR HEALTH interventions for loneliness in people who experience psychosis. Psychol Psychother. (2025) 98:478–500. doi: 10.1111/papt.12574, PMID: 39878384 PMC12065070

[81] Vickery CD Gontkovsky ST Wallace JJ Caroselli JS . Group psychotherapy focusing on self-concept change following acquired brain injury: A pilot investigation. Rehabil Psychol. (2006) 51:30–5. doi: 10.1037/0090-5550.51.1.30

[82] Corsten S Schimpf EJ Konradi J Keilmann A Hardering F . The participants’ perspective: how biographic-narrative intervention influences identity negotiation and quality of life in aphasia. Int J Lang Commun Disord. (2015) 50:788–800. doi: 10.1111/1460-6984.12173, PMID: 26123497

[83] Gelech J Bayly M Desjardins M . Constructing robust selves after brain injury: positive identity work among members of a female self-help group. Neuropsychol Rehabil. (2019) 29:456–76. doi: 10.1080/09602011.2017.1308872, PMID: 28393594

[84] Elderton A Clarke S Jones C Stacey J . Telling our story: A narrative therapy approach to helping lesbian, gay, bisexual and transgender people with a learning disability identify and strengthen positive self-identity stories. Br J Learn Disabil. (2013) 42:301–7. doi: 10.1111/bld.12075

[85] Bradshaw W Roseborough D . Evaluating the effectiveness of cognitive-behavioral treatment of residual symptoms and impairment in schizophrenia. Res Soc Work Pract. (2004) 14:112–20. doi: 10.1177/1049731503257872

[86] Lukoff D Wallace CJ Liberman RP Burke K . A holistic program for chronic schizophrenic patients. Schizophr Bull. (1986) 12:274–82. doi: 10.1093/schbul/12.2.274, PMID: 3520804

[87] McCay E Tibbo P Conrad G Aiello A Crocker C Beanlands H . The impact of a transitional intervention for youth living with early psychosis: A mixed methods study. Int Health Trends Perspect. (2021) 1:96–114. doi: 10.32920/ihtp.v1i1.1423

[88] Cutler M Nelson MLA Nikoloski M Kuluski K . Mindful connections: The role of a peer support group on the psychosocial adjustment for adults recovering from brain injury. J Soc Work Disabil Rehabil. (2016) 15:260–84. doi: 10.1080/1536710X.2016.1220879, PMID: 27494439

[89] von Mensenkampff B Ward M Kelly G Cadogan S Fawsit F Lowe N . The value of normalization: Group therapy for individuals with brain injury. Brain Injury. (2015) 29:1292–9. doi: 10.3109/02699052.2015.1042407, PMID: 26084318

[90] Kelly A Ponsford J Couchman G . Impact of a family-focused intervention on self-concept after acquired brain injury. Neuropsychol Rehabil. (2013) 23:563–79. doi: 10.1080/09602011.2013.795903, PMID: 23656483

[91] Sathananthan N Morris EMJ das Nair R Gillanders D Wright B Wong D . Evaluating the VaLiANT (Valued Living After Neurological Trauma) group intervention for improving adjustment to life with acquired brain injury: A pilot randomized controlled trial. Neuropsychol Rehabil. (2025) 35:1918–46. doi: 10.1080/09602011.2025.2476074, PMID: 40101111

[92] de Wit M Moonen X Douma J . Aanbevelingen voor het aanpassen, uitvoeren en ontwikkelen van gedragsveranderende interventies voor personen met een licht verstandelijke beperking. Utrecht: Landelijk Kenniscentrum LVB (2023).

[93] Fancourt D Finn S . What is the evidence on the role of the arts in improving health and well-being? A scoping review (2019). 32091683

[94] Peters LA Gomersall T Booth A Lucock M . Community arts, identity and recovery: A realist review of how community-based arts activities enables the identity change recovery process from serious mental illness. J Community Appl Soc Psychol. (2023) 34. doi: 10.1002/casp.2751

[95] Stickley T Wright N Slade M . The art of recovery: outcomes from participatory arts activities for people using mental health services. J Ment Health. (2018) 27:367–73. doi: 10.1080/09638237.2018.1437609, PMID: 29447483

[96] Lewis F Groot B Krans KLS van Leeuwen B van de Wal-Huisman H Abma TA . Arts in health in the Netherlands: A national agenda. (2024).

[97] Zbranca R Dâmaso M Blaga O Kiss K Dascl MD Yakobson D . CultureForHealth Report. Culture’s contribution to health and well-being. A report on evidence and policy recommendations for Europe. CultureForHealth Culture Action Europe (2022).

[98] Martela F Ryan RM Steger MF . Meaningfulness as satisfaction of autonomy, competence, relatedness, and beneficence: Comparing the four satisfactions and positive affect as predictors of meaning in life. J Happiness Stud. (2018) 19:1261–82. doi: 10.1007/s10902-017-9869-7

[99] White M . Maps of narrative practice. New York: W.W. Norton (2007).

[100] Muthert H . Daar aansluiten waar mensen niet willen zijn. In: Körver J Walton M den Toom N , editors. Richting, repertoire en resultaat: Uitkomsten van het Nederlandse Case Studies Project Geestelijke Verzorging (2016 - 2021). UCGV (2023). p. 190–200.

[101] Westerhof GJ Beernink J Sools A . Who am i? A life story intervention for persons with intellectual disability and psychiatric problems. Intellect Dev Disabil. (2016) 54:173–86. doi: 10.1352/1934-9556-54.3.173, PMID: 27268473

[102] Yanos PT Roe D Lysaker PH . Narrative enhancement and cognitive therapy: A new group-based treatment for internalized stigma among persons with severe mental illness. Int J Group Psychother. (2011) 61:576–95. doi: 10.1521/ijgp.2011.61.4.576, PMID: 21985260 PMC3191919

[103] Amiot CE de la Sablonniere R Terry DJ Smith JR . Integration of social identities in the self: Toward a cognitive-developmental model. Pers Soc Psychol Rev. (2007) 11:364–88. doi: 10.1177/1088868307304091, PMID: 18453468

[104] Ellis-Hill C Thomas S Gracey F Lamont-Robinson C Cant R Marques EMR . HeART of Stroke: Randomised controlled, parallel-arm, feasibility study of a community-based arts and health intervention plus usual care compared with usual care to increase psychological well-being in people following a stroke. BMJ Open. (2019) 9:e021098. doi: 10.1136/bmjopen-2017-021098, PMID: 30852528 PMC6429750

[105] Dunlop W . Narrative identity’s nomological network: Expanding and organizing assessment of the storied self. Pers Sci. (2021) 2:1–31. doi: 10.5964/ps.6469

[106] Mizock L Russinova Z Shani R . New roads paved on losses. Qual Health Res. (2014) 24:1481–91. doi: 10.1177/1049732314548686, PMID: 25168704

[107] Baker FA Tamplin J MacDonald RAR Ponsford J Roddy C Lee C . Exploring the self through songwriting: An analysis of songs composed by people with acquired neurodisability in an inpatient rehabilitation program. J Music Ther. (2017) 54:35–54. doi: 10.1093/jmt/thw018, PMID: 28391303

[108] Cruwys T Steffens NK Haslam SA Haslam C Jetten J Dingle GA . Social Identity Mapping: A procedure for visual representation and assessment of subjective multiple group memberships. Br J Soc Psychol. (2016) 55:613–42. doi: 10.1111/bjso.12155, PMID: 27578549

[109] van der Gaag M Famiglini M Kunnen S Bosma H . Groningen Identity Development Scale: Landscape version 2 (GIDS-L2)2022. Available online at: https://research.rug.nl/en/publications/b4cd7775-fc73-4be4-b5cc-40bc9d94c1d9 (Accessed December 6, 2024).

[110] Kidd SA Veltman A Gately C Chan KJ Cohen JN . Lesbian, gay, and transgender persons with severe mental illness: negotiating wellness in the context of multiple sources of stigma. Am J Psychiatr Rehabil. (2011) 14:13–39. doi: 10.1080/15487768.2011.546277

[111] Lysaker PH Gagen E Klion R Zalzala A Vohs J Faith LA . Metacognitive reflection and insight therapy: A recovery-oriented treatment approach for psychosis. Psychol Res Behav Manage. (2020) 13:331–41. doi: 10.2147/PRBM.S198628, PMID: 32308511 PMC7135118

