# Supplementary material for: Interventions targeting identity in adults with psychosis, severe mental illness, brain injury, or intellectual disability: a transdiagnostic scoping review
Source: Front Psychiatry. 2026 Feb 5;17:1674898. doi: 10.3389/fpsyt.2026.1674898 (PMC12916650; doi:10.3389/fpsyt.2026.1674898)
Supplement: Supplementary file 1 [file SupplementaryFile1.docx]

Supplementary Material 1 – adjustments to the protocol

# Adjustments to the protocol after publishing the initial protocol to the OSF repository.

**Refinement: ´identity as means towards another end´.** During the selection process we discovered that some studies described activities related to identity exploration, but primarily used these exercises as a means to achieve other goals (e.g. goal setting). Additionally, these studies did not evaluate the effects of the intervention on identity. Our initial protocol specified that for a study to be included, ´Identity should be a prespecified aim of the intervention study, either because the intervention clearly aims to impact identity, or because the impact of the intervention on identity is formally evaluated´. We clarified this further during our search by specifying that studies appearing to focus on identity exploration (e.g., mentioning identity in the introduction or including identity exercises in the intervention) would not be included if the authors indicated that identity exploration was primarily used to achieve another primary goal and was not formally evaluated (thus not being a central aim of the intervention study).

**Refinement: exclude certain activities that some consider interventions while others do not.** After discussing with the research team, we decided during the selection process to exclude activities such as volunteering, occupation, housing, self-advocacy group membership, hobbies, and diagnostic tests. These were excluded for several reasons: they were not consistently regarded as interventions by some screeners, they were not specifically focused on identity (re)building, they varied significantly in activities, duration, and they lacked a fixed end date, making them difficult to replicate. Additionally, these activities did not have a clear psychosocial, therapeutic, or health-related primary aim, as individuals would typically continue them daily even after achieving any therapeutic goals.

**Addition: exclusion of N=1 case studies.** Initially, the protocol did not specify the exclusion of N=1 case studies. However, we decided to exclude them for practical reasons (there were many such case studies, and studies were extensive and heterogeneous and difficult to summarize) and due to their limited generalizability and often ambiguous or vague definitions of identity.

# Summary of exclusion criteria:

| Exclusion criteria |
| --- |
| 50% or more participants not primarily described as fitting with our diagnostic criteria |
| Participants are children (<18) |
| Identity is a post-hoc finding (not pre-specified) |
| Focus limited to unidimensional aspect of identity: e.g. gender identity, occupational identity, etc. |
| Identity not evaluated AND Identity is not a (clear) aim. |
| Identity not evaluated AND only a very limited part or aim of the intervention, for example because identity-exercises are used mainly as a means towards another end. |
| Activities and situations such as volunteering, occupation, housing, self-advocacy group membership, and hobbies. |
| General care or therapy that does not specifically target identity, and specific components are not explicated: e.g. hospitalization, rehabilitation, physiotherapy, psychotherapy |
| Wrong source: not peer-reviewed, no formal intervention evaluation, N=1 case study, opinion paper, editorial, conference abstract, book chapter, theoretical article, review, dissertation. |
