# Supplementary material for: Interventions targeting identity in adults with psychosis, severe mental illness, brain injury, or intellectual disability: a transdiagnostic scoping review
Source: Front Psychiatry. 2026 Feb 5;17:1674898. doi: 10.3389/fpsyt.2026.1674898 (PMC12916650; doi:10.3389/fpsyt.2026.1674898)
Supplement: Supplementary file 2 [file SupplementaryFile2.docx]

Supplementary Material 2 – excluded studies

# List with examples of excluded studies

The following list provides examples of excluded articles, many of which were excluded after thorough discussions with the research team. These discussions often addressed disagreements between reviewers or ambiguity in the articles themselves. The purpose of this list is to illustrate the breadth of the field and to highlight the types of studies that could be considered under different review criteria and to help readers understand the application of the inclusion criteria. Specifically, the list includes:

- A selection of the most relevant examples of studies that did not meet the inclusion criteria but might have been included with slight adjustments to the criteria.
- The studies that posed the greatest challenges or required the most subjective judgment during the inclusion decision process.

| ***Reference*** | ***Target group*** | ***Type of intervention*** | ***Notes*** |
| --- | --- | --- | --- |
| *Wrong publication type* | | | |
| Lombardo, A. (2014). A self-concept group intervention pilot study for individuals with an acquired brain injury: The relationship between self-concept, depression and quality of life. *In Dissertation Abstracts International: Section B: The Sciences and Engineering, 74*(10–B(E)). | ABI | Self-concept group | Dissertation |
| Hasson-Ohayon, I., Cheli, S., & Lysaker, P. H. (2021). Emerging psychotherapeutic approaches to addressing self-experience in schizophrenia spectrum disorders. *Journal of clinical psychology, 77*(8), 1781–1785. https://doi.org/10.1002/jclp.23223 | Schizophrenia | Psychotherapeutic approaches | Editorial and theoretical |
| *Not possible to retrieve the full text article* | | | |
| Peixin, M., Richang, Z., Zhuoji, C., Dahong, C., Liang, M., Jun, L., & Yu, L. (2005). Group intervention for schizophrenia inpatient with art as medium. *Acta Psychologica Sinica, 37*(3), 403–412. | Schizophrenia | Group intervention | Note: article is also not in English |
| Jakubik, A., Watras, J., Jakubik, A. M., Jakimow-Venulet, B., & Waniek, J. (1986). Wstępna ocena skuteczności krótkoterminowej psychoterapii grupowej w skojarzonym leczeniu schizofrenii paranoidalnej = Preliminary assessment of the effectiveness of short-term group psychotherapy in combined treatment of paranoid schizophrenia. *Psychiatria Polska, 20*(3), 208–214. | Schizophrenia | Short term group treatment | Note: article is also not in English |
| Magid, B. (1984). Some contributions of self psychology to the treatment of borderline and schizophrenic patients. *Dynamic Psychotherapy, 2*(2), 101–111. | Borderline and schizophrenia | Unclear if it is about an intervention. | Note: probably would not have been included in the review because it does not seem an intervention, but we could not retrieve the full text to check this. |
| *Intervention included via another paper, but this paper did not meet the criteria.* | | | |
| Corsten, S., Konradi, J., Schimpf, E. J., Hardering, F., & Keilmann, A. (2013). Improving quality of life in aphasia—Evidence for the effectiveness of the biographic-narrative approach. *Aphasiology, 28*(4), 440–452. https://doi.org/10.1080/02687038.2013.843154 | ABI | Biographic–narrative intervention for aphasia | This article reports quantitative outcomes (not related to identity), a study reporting qualitative outcomes was included in our review. The main aim of this article is formulated as adjustment. Identity is not a clearly pre-specified aim/focus of the article. This article is mainly excluded because identity not a clear aim of the intervention and not clearly evaluated. |
| Couchman, G., McMahon, G., Kelly, A., & Ponsford, J. (2014). A new kind of normal: qualitative accounts of Multifamily Group Therapy for acquired brain injury. Neuropsychological rehabilitation, 24(6), 809–832. https://doi.org/10.1080/09602011.2014.912957 | ABI | Multifamily group therapy | The quantitative evaluation of this study was included in the review. The evaluation is with < 50% people with ABI (other participants are family members). This article is mainly excluded because identity is a minor part of the intervention and not evaluated or not a clearly pre-specified aim. |
| Mizock, L., Russinova, Z., & Shani, R. (2014). New roads paved on losses: Photovoice perspectives about recovery from mental illness. *Qualitative Health Research, 24*(11), 1481–1491. https://doi.org/10.1177/1049732314548686 | SMI | Recovery narrative photovoice intervention | Thematic analysis of art works produced during the intervention. The study in which the intervention is discussed is included in the review. This article is excluded because it is an analysis of creative work made during the identity-intervention, not evaluation of intervention or intervention effect itself. |
| Baker, F. A., Tamplin, J., MacDonald, R. A., Ponsford, J., Roddy, C., Lee, C., & Rickard, N. (2017). Exploring the Self through Songwriting: An Analysis of Songs Composed by People with Acquired Neurodisability in an Inpatient Rehabilitation Program. *Journal of music therapy, 54*(1), 35–54. https://doi.org/10.1093/jmt/thw018 | Spinal cord injury or ABI | Therapeutic songwriting | Analysis of the songs written during the intervention. The study in which the intervention is discussed is included in the review. This article is excluded because it is an analysis of creative work made during the identity-intervention, not evaluation of intervention or intervention effect itself. |
| Sathananthan, N., Morris, E., Gillanders, D., das Nair, R., Knox, L., & Wong, D. (2025). Rebuilding the self through valued action and group connections after acquired brain injury: Participant perspectives of the VaLiANT group intervention. *Neuropsychological rehabilitation*, *35*(4), 728–756. https://doi.org/10.1080/09602011.2024.2359992 | ABI | VaLiANT (Valued Living After  Neurological Trauma) | An article reporting quantitative outcomes of this intervention is reported in the review. This article is excluded because the identity findings are post-hoc and not a pre-specified aim. |
| *Identity is a minor part of the intervention and not evaluated or not a clearly pre-specified aim* | | | |
| Marshall, J., Devane, N., Talbot, R., Caute, A., Cruice, M., Hilari, K., MacKenzie, G., Maguire, K., Patel, A., Roper, A., & Wilson, S. (2020). A randomised trial of social support group intervention for people with aphasia: A Novel application of virtual reality. *PloS one, 15*(9), e0239715. https://doi.org/10.1371/journal.pone.0239715 | ABI | Social support intervention |  |
| Bronken, B. A., Kirkevold, M., Martinsen, R., Wyller, T. B., & Kvigne, K. (2012). Psychosocial well-being in persons with aphasia participating in a nursing intervention after stroke. *Nursing research and practice, 2012,* 568242. https://doi.org/10.1155/2012/568242 | ABI | Nursing intervention |  |
| *Wrong target group or potentially wrong target group* | | | |
| Butera-Prinzi, F., Charles, N., & Story, K. (2014). Narrative family therapy and group work for families living with acquired brain injury. *Australian and New Zealand Journal of Family Therapy, 35*(1), 81–99. https://doi.org/10.1002/anzf.1046 | ABI | Family therapy, e.g. tree of life exercise | Wrong target group (family) and topic mildly related: is about how whole families cope with change after TBI of the father. |
| Rodriquez, J., Gupta, A., Ballard, S. C., & Siperstein, G. N. (2023). Positive identity development through community engagement among youth with intellectual and developmental disabilities. *Journal of Applied Research in Intellectual Disabilities, 36*(4), 758–767. https://doi.org/10.1111/jar.13091 | Youth (16 to 20) with intellectual or developmental disability | Community engagement | Participants' description of themselves were used instead of a diagnostic label, however based upon these descriptions it was not clear if 50% of the participants had an intellectual disability. This could not be clarified by the author upon request. |
| *No evaluation of intervention* | | | |
| Chow E. O. (2015). Narrative therapy an evaluated intervention to improve stroke survivors' social and emotional adaptation. *Clinical rehabilitation, 29*(4), 315–326. https://doi.org/10.1177/0269215514544039 | Stroke | Narrative therapy | No evaluation, this is just a description of a framework, with some case examples and a few evaluative notes. |
| *Care in general* | | | |
| Di Vita, A., Procacci, M. A., Bellagamba, M., Jacomini, M., Massicci, R., & Ciurli, M. P. (2022). Psychotherapy and Art Therapy: A pilot study of group treatment for patients with traumatic brain injury. *Journal of health psychology, 27*(4), 836–846. https://doi.org/10.1177/1359105320967099 | TBI | psychotherapy and art therapy | A focus on identity is mentioned, but this remains very vague and is not really reflected in the discussion. It seems identity is defined here very broadly, as diverse psychological aspects. It is measured with the BIGI (a measure of grief, loss & adjustment). |
| Roenn-Smidt, H., Larsen, K., & Pallesen, H. (2020). The practices of body in rehabilitation after stroke: a qualitative study of how physiotherapy affects identity reconstruction. *European Journal of Physiotherapy, 23*(5), 270–278. https://doi.org/10.1080/21679169.2020.1730440 | Stroke | Physiotherapy | Focus on body work and influence of this on (bodily) identity. |
| Allen, J., Burbach, F., & Reibstein, J. (2013). 'A different world' individuals' experience of an integrated family intervention for psychosis and its contribution to recovery. *Psychology and psychotherapy, 86*(2), 212–228. https://doi.org/10.1111/j.2044-8341.2011.02057.x | Psychosis | Integrated family intervention | Focus mainly on sense of self and multiple self-positions in the context of recovery. |
| Corr, S., Phillips, C. J., & Walker, M. (2004). Evaluation of a pilot service designed to provide support following stroke: a randomized cross-over design study. *Clinical rehabilitation, 18*(1), 69–75. https://doi.org/10.1191/0269215504cr703oa | Stroke | Supportive day service |  |
| Tiedemann, L., Dulek, J., Lemoncello, R., & Foidel, S. (2024). Examining Personal Identity and the Influence of an Occupation-Based Prevocational Program Following Traumatic Brain Injury: A Multiple Case Study. Occupational therapy in health care, 1–24. Advance online publication. https://doi.org/10.1080/07380577.2024.2437689 | TBI | Occupation-based program. | Relatively broad occupation-based program with different activities per individual. |
| *Not considered an intervention (e.g. not a structured programme or intervention but naturalistic and everyday context)* | | | |
| Sveen, U., Søberg, H. L., & Østensjø, S. (2016). Biographical disruption, adjustment and reconstruction of everyday occupations and work participation after mild traumatic brain injury. A focus group study. *Disability and rehabilitation, 38*(23), 2296–2304. https://doi.org/10.3109/09638288.2015.1129445 | TBI | Everyday occupation, return to work program. |  |
| Carless, D., & Douglas, K. (2008). Narrative, identity and mental health: How men with serious mental illness re-story their lives through sport and exercise. *Psychology of Sport and Exercise, 9*(5), 576–594. https://doi.org/10.1016/j.psychsport.2007.08.002 | SMI | Sport and exercises |  |
| Pérez-Corrales, J., Pérez-de-Heredia-Torres, M., Martínez-Piedrola, R., Sánchez-Camarero, C., Parás-Bravo, P., & Palacios-Ceña, D. (2019). 'Being normal' and self-identity: the experience of volunteering in individuals with severe mental disorders-a qualitative study. *BMJ open, 9*(3), e025363. https://doi.org/10.1136/bmjopen-2018-025363 | Psychosis | Volunteering |  |
| Piat, M., Seida, K., Sabetti, J., & Padgett, D. (2017). (Em)placing recovery: Sites of health and wellness for individuals with serious mental illness in supported housing. *Health & place, 47*, 71–79. https://doi.org/10.1016/j.healthplace.2017.07.006 | SMI | Move to supportive housing, therapeutic landscapes | Focus partly on identity, as part of recovery |
| Norlander, A., Iwarsson, S., Jönsson, A. C., Lindgren, A., & Månsson Lexell, E. (2022). Participation in social and leisure activities while re-constructing the self: understanding strategies used by stroke survivors from a long-term perspective. Disability and rehabilitation, *44(*16*),* 4284–4292. https://doi.org/10.1080/09638288.2021.1900418 | Stroke | Social activity and leisure participation |  |
| Werner, S., & Hochman, Y. (2019). On self-identity: the process of inclusion of individuals with intellectual disabilities in the military. *Disability and rehabilitation, 41*(14), 1639–1646. https://doi.org/10.1080/09638288.2018.1443158 | ID | Participation in the army | Note: we first included this and started with initial data-extraction, this can be retrieved upon request. |
| Anderson, S., & Bigby, C. (2017). Self-Advocacy as a Means to Positive Identities for People with Intellectual Disability: 'We Just Help Them, Be Them Really'. *Journal of applied research in intellectual disabilities : JARID, 30*(1), 109–120. https://doi.org/10.1111/jar.12223 | ID | Self-advocacy | Note: we first included this and started with initial data-extraction, this can be retrieved upon request. |
| Mineur, T., Tideman, M., & Mallander, O. (2017). Self-advocacy in Sweden—an analysis of impact on daily life and identity of self-advocates with intellectual disability. *Cogent Social Sciences, 3*(1). https://doi.org/10.1080/23311886.2017.1304513 | ID | Self-advocacy | Note: we first included this and started with initial data-extraction, this can be retrieved upon request. |
| *Case study* | | | |
| Shadden, B., Agan, J.P. (2004). Renegotiation of Identity: The Social Context of Aphasia Support Groups. *Topics in Language Disorders 24*(3), 174-186. | ABI | Aphasia support groups |  |
| Gurr, B., & Foxhall, M. (2014). Rebuilding identity after brain injury: Standard cognitive and music-evoked autobiographical memory training...including commentary by Shinoda J and Baird A. *International Journal of Therapy & Rehabilitation, 21*(6), 289–295. | ABI | Cognitive and music evoked autobiographical memory training |  |
| *Post-hoc* | | | |
| Carcello, K., & McLennon, S. (2022). "Personal road map for recovery:" examining the therapeutic use of weblogs by stroke survivors with aphasia. *Disability and rehabilitation, 44*(10), 1933–1938. https://doi.org/10.1080/09638288.2020.1812122 | Stroke | Weblogs |  |
| Northcott, S., Simpson, A., Thomas, S., Barnard, R., Burns, K., Hirani, S. P., & Hilari, K. (2021). "Now I Am Myself": Exploring How People With Poststroke Aphasia Experienced Solution-Focused Brief Therapy Within the SOFIA Trial. *Qualitative health research, 31*(11), 2041–2055. https://doi.org/10.1177/10497323211020290 | Stroke | Focused brief therapy |  |
| *Aim is ‘self-concept’ or identity, but this is not a clear focus of the intervention and the quantitative tool used is a proxy that merely focuses on related topics.* | | | |
| McGaw, S., Ball, K., & Clark, A. (2002). The effect of group intervention on the relationships of parents with intellectual disabilities. *Journal of Applied Research in Intellectual Disabilities, 15*(4), 354–366. https://doi.org/10.1046/j.1468-3148.2002.00143.x | ID | Home based teaching programme for parents | The Judson Self-Rating  Scale (Judson  &  Burden  1980): focuses partly on feelings and judgements about the children and professionals and partly on self-concept. |
| Hartke, R.J., King, R.B., Denby, F. (2007). The use of writing groups to facilitate adaptation after stroke. *Topics in Stroke Rehabilitation.* *14*(1), 26-37. https://doi.org/10.1310/tsr1401-26 | Stroke | Essay writing about recovery | They use the personal opinion questionnaire: “One of the four subscales on positive sense of identity as a person with a disability was utilized in the current study. This subscale measures the degree to which disability can be a source of strength and special insight.” |
| Yingying, P., Zang, L., Wang, X., & Yang, X. (2022). Effect of Continuous Care Combined with Constraint-Induced Movement Therapy Based on a Continuing Care Health Platform on MBI and FMA Scores of Acute Stroke Patients. *Journal of healthcare engineering*, *2022*, 5299969. https://doi.org/10.1155/2022/5299969 | Stroke | Constraint-induced movement therapy | Used measure is the ESCA (Exercise of Self-care Agency), and one of the factors of this survey is about emotional aspects of self-care and labeled as self-concept. |
| *Identity as process, measured on a micro-level:*  *analysis of moments in which identity is constructed or uttered* | | | |
| Kovarsky, D., Shaw, A., & Adingono-Smith, M. (2007). The construction of identity during group therapy among adults with traumatic brain injury. *Communication & medicine, 4(*1), 53–66. https://doi.org/10.1515/CAM.2007.029 | TBI | Speech-language therapy | This is an observational study, the authors observe video tapes of 6 sessions, and describe in the paper the moments were 'identity' was uttered. The authors then mention where the therapists misses opportunities to establish a positive identity or talk about identity issues. |
| Rojo-Pardo, C., & Iñiguez, L. (2023). The impact of inclusion practices on the identity of people diagnosed with severe mental illness: Radio Nikosia. *Ciencia & saude coletiva, 28(*6), 1789-1798. | SMI | Radio interviews | Analysis of utterances of identity in radio interview with people with SMI |
| Claudia Krautkremer, Louise C. Keegan, Rimke Groenewold, & Elizabeth Spencer. (2024). “I’m a Magical Helping Guy”, Using Systemic Functional Linguistics to Examine Identity in a Virtual Table-Top Role-Playing Game after Brain Injury. *Topics in Language Disorders*, *44*(3), 241–260. https://doi.org/10.1097/TLD.0000000000000344 | ABI | Virtual Table-Top Role-Playing Game | Analysis of the utterances of individuals related to identity in before the intervention and during the intervention. The study does not evaluate the effect of the intervention. |
| *Focus on structural aspect of self-concept, instead of content/descriptive self* | | | |
| Oudejans, S., de Winter, L., van Weeghel, J., Sanches, S., & Hasson-Ohayon, I. (2022). Feasibility and outcomes of narrative enhancement and cognitive therapy (NECT) for reducing self-stigma among people with severe mental illness in the Netherlands: A pilot study. *Psychiatric rehabilitation journal, 45*(3), 255–265. https://doi.org/10.1037/prj0000526 | SMI | NECT | Structural aspect of self-concept: degree of certainty about self-concept. It is related to identity but does not fit within our definition. |
| Ellett, L., Kingston, J., Tarant, E., Kouimtsidis, C., Vivarelli, L., & Chadwick, P. (2023). Self-Structure in Persecutory Delusions. *Behavior therapy, 54*(1), 132–140. https://doi.org/10.1016/j.beth.2022.07.011 | Schizophrenia spectrum | MBCT | It´s about compartmentalization (mix of positive/negative characteristics), proportion of negative attributes, and differential importance (are positive or negative self-aspects more important?). This seems to be more about the structure of self-organization. |
| *Identity is underlying the intervention and theoretical background, but not explicitly an aim of the evaluation. Identity is seen as a mechanism, and other aims are more central in the intervention description.* | | | |
| D'Cruz, K., Douglas, J., & Serry, T. (2020). Narrative storytelling as both an advocacy tool and a therapeutic process: Perspectives of adult storytellers with acquired brain injury. *Neuropsychological rehabilitation, 30*(8), 1409–1429. https://doi.org/10.1080/09602011.2019.1586733 | ABI | Narrative storytelling | Identity is not explicitly mentioned as an aim, but based on the introduction and the extensive information about this topic it seems that the authors think that identity-construction is a an important aspect of story-telling. |
| Pijnenborg, G. H. M., de Vos, A. E., Timmerman, M. E., Van der Gaag, M., Sportel, B. E., Arends, J., Koopmans, E. M., Van der Meer, L., & Aleman, A. (2019). Social cognitive group treatment for impaired insight in psychosis: A multicenter randomized controlled trial. *Schizophrenia research, 206*, 362–369. https://doi.org/10.1016/j.schres.2018.10.018 | Psychosis | Reflex – social cognitive group | Focus of the intervention is on narrative, stigma, and perspective taking, but the aims are: insight, stigma reduction and self-reflection (this is confirmed by the study author). |
| Blairy, S., Neumann, A., Nutthals, F., Pierret, L., Collet, D., & Philippot, P. (2008). Improvements in autobiographical memory in schizophrenia patients after a cognitive intervention: a preliminary study. *Psychopathology, 41*(6), 388–396. https://doi.org/10.1159/000155217 | Schizophrenia | Autobiographical memory | Focus of the intervention is on memory, self-definition and goals, but the aim is autobiographical memory specificity. |
| Yanos, P. T., Roe, D., & Lysaker, P. H. (2011). Narrative enhancement and cognitive therapy: a new group-based treatment for internalized stigma among persons with severe mental illness. *International journal of group psychotherapy, 61*(4), 577–595. https://doi.org/10.1521/ijgp.2011.61.4.576 | SMI | NECT | Note: multiple studies have been published about NECT, not all of those were found with our search. |
| de Jong, S., van Donkersgoed, R. J. M., Timmerman, M. E., Aan Het Rot, M., Wunderink, L., Arends, J., van Der Gaag, M., Aleman, A., Lysaker, P. H., & Pijnenborg, G. H. M. (2019). Metacognitive reflection and insight therapy (MERIT) for patients with schizophrenia. *Psychological medicine, 49*(2), 303–313. https://doi.org/10.1017/S0033291718000855 | Schizophrenia | MERIT | Note: multiple studies have been published about MERIT or metacognitive therapy, not all of those were found with our search. |
| Ylvisaker, M., McPherson, K., Kayes, N., & Pellett, E. (2008). Metaphoric identity mapping: facilitating goal setting and engagement in rehabilitation after traumatic brain injury. *Neuropsychological rehabilitation, 18*(5-6), 713–741. https://doi.org/10.1080/09602010802201832 | TBI | Metaphoric identity mapping or identity informed goal setting | Identity used to identify meaningful goals. |
| *Focus on a small part of identity, not on multiple facets* | | | |
| Tarrant, M., Lamont, R. A., Carter, M., Dean, S. G., Spicer, S., Sanders, A., & Calitri, R. (2021). Measurement of Shared Social Identity in Singing Groups for People With Aphasia. *Frontiers in psychology, 12*, 669899. https://doi.org/10.3389/fpsyg.2021.669899 | ABI | Singing groups | Focus on shared social identity as a member of the intervention group |
| Gutman S. A. (1999). Alleviating gender role strain in adult men with traumatic brain injury: an evaluation of a set of guidelines for occupational therapy. *The American journal of occupational therapy, 53*(1), 101–110. https://doi.org/10.5014/ajot.53.1.101 | TBI | Occupational therapy intervention based on guidelines to enhance gender role satisfaction | Rebuilding self-identified gendered social roles and activities |
| *Intervention not evaluated with the clinical target group, but only with professionals* | | | |
| Moya, H. (2009). Identities on paper: Constructing lives for people with intellectual disabilities in life story books. *Narrative Inquiry, 19*(1), 135–153. https://doi.org/10.1075/ni.19.1.08moy | ID | Life story books |  |
| *Intervention described, but not formally evaluated* | | | |
| Kidd, S. A., Hasan, N., & Trapp, J. (2015). Exploring the use of digital picture frames on schizophrenia inpatient wards. *Psychiatric services, 66*(3), 330. https://doi.org/10.1176/appi.ps.660307 | Schizophrenia | Using picture frames |  |
| Chow E. O. (2015). Narrative therapy an evaluated intervention to improve stroke survivors' social and emotional adaptation. *Clinical rehabilitation, 29*(4), 315–326. https://doi.org/10.1177/0269215514544039 | Stroke | Narrative therapy |  |
| Belmonte Almagro, M. L., & Bernárdez-Gómez, A. (2021). Evaluation of Self-Concept in the Project for People with Intellectual Disabilities: “We Are All Campus”. *Journal of Intelligence, 9*(4), 50. https://doi.org/10.3390/jintelligence9040050 | Intellectual disability | Job training and inclusion through a university-based programme |  |
| *Identity not a clear aim of the intervention and not clearly evaluated* | | | |
| Kaimal, G., Jones, J.P., Dieterich-Hartwell, R., Acharya, B., Wang, X. (2019). Evaluation of long- and short-term art therapy interventions in an integrative care setting for military service members with post-traumatic stress and traumatic brain injury. *The Arts in Psychotherapy, 62*, 28-36. https://doi.org/10.1016/j.aip.2018.10.003. | TBI | Art therapy | Relatively general intervention and identity seems just a small part of it. |
| Ellis-Hill, C., Thomas, S., Gracey, F., Lamont-Robinson, C., Cant, R., Marques, E. M. R., Thomas, P. W., Grant, M., Nunn, S., Paling, T., Thomas, C., Werson, A., Galvin, K. T., Reynolds, F., & Jenkinson, D. (2019). HeART of Stroke: randomised controlled, parallel-arm, feasibility study of a community-based arts and health intervention plus usual care compared with usual care to increase psychological well-being in people following a stroke. *BMJ open, 9*(3), e021098. https://doi.org/10.1136/bmjopen-2017-021098 | Stroke | Community-based arts and health group | Researchers administered the HISDS (a scale which is considered as an identity outcome in our review), but participants found it difficult to understand the HISDS and this was reflected in the patterns, thus the outcomes of this scale were not reported. |
| Banerjee, S., & Roy, L. (2024). Hand-building the sense of self: art therapy with Indian women in an assisted-living facility for the ‘homeless.’ International Journal of Art Therapy, 1–11. https://doi.org/10.1080/17454832.2024.2416904 | SMI | Art therapy | The aim of the article is to evaluate ‘sense of self’, but the operationalization of this is not very clear. |
| *Recovery oriented program: identity is not a clear main aim/focus, but part of the intervention and not clearly evaluated.* | | | |
| Thomas, N., Farhall, J., Foley, F., Leitan, N. D., Villagonzalo, K. A., Ladd, E., Nunan, C., Farnan, S., Frankish, R., Smark, T., Rossell, S. L., Sterling, L., Murray, G., Castle, D. J., & Kyrios, M. (2016). Promoting Personal Recovery in People with Persisting Psychotic Disorders: Development and Pilot Study of a Novel Digital Intervention. *Frontiers in psychiatry, 7*, 196. https://doi.org/10.3389/fpsyt.2016.00196 | Persisting psychosis | Recovery website based on CHIME + using this within therapy | One question (yes/no), in one of the outcome measures is about identity.  (Note: we started with initial data-extraction, this can be retrieved upon request) |
| Song, L.-Y., & Hsu, S.-T. (2023). Exploring the experiences of personal recovery among mental health consumers and their caregivers receiving strength-based family interventions. *International Journal of Mental Health Promotion, 25*(8), 915–925. https://doi.org/10.32604/ijmhp.2023.019349 | SMI | Employing a recovery-focused approach to family focused intervention | Relatively general intervention. Findings report changes in `sense of self`. |
| *Post-hoc and focus of the intervention is partly on ‘sense of self’, operationalization of self is broad* | | | |
| Goodliffe, L., Hayward, M., Brown, D., Turton, W., & Dannahy, L. (2010). Group person-based cognitive therapy for distressing voices: views from the hearers. *Journal of the Society for Psychotherapy Research, 20*(4), 447–461. https://doi.org/10.1080/10503301003671305 | Schizophrenia or schizoaffective disorder | Group cognitive therapy for distressing voices | The focus is on the relation between person and voice. The topics are described with terms such as: relation between person/voice; changing and fluid sense of self, beliefs about voices, positive beliefs about others and self-schemata to counterbalance negative voices. |
| May, K., Strauss, C., Coyle, A., & Hayward, M. (2012). Person-based cognitive therapy groups for distressing voices: A thematic analysis of participant experiences of the therapy. *Psychosis:* *Psychological, Social and Integrative Approaches, 6*(1). https://doi.org/10.1080/17522439.2012.708775 | Psychosis | Group cognitive therapy for distressing voices |  |
