# Supplementary material for: Interventions targeting identity in adults with psychosis, severe mental illness, brain injury, or intellectual disability: a transdiagnostic scoping review
Source: Front Psychiatry. 2026 Feb 5;17:1674898. doi: 10.3389/fpsyt.2026.1674898 (PMC12916650; doi:10.3389/fpsyt.2026.1674898)
Supplement: Supplementary file 3 [file SupplementaryFile3.docx]

| **Table 1: Quantitative findings and feasibility** | | |
| --- | --- | --- |
| ***A) Direct SMI interventions: interventions with a strong focus on identity and self-views*** | | |
| **Intervention (reference)** | **Quantitative results** (underlined: significant, *italic: non significant,* normal text: significance not mentioned*)* | **Participant feedback and feasibility** |
| Continuous Identity Cognitive Therapy (Sokol et al., 2021) | **Identity:** Significant increased sense of future self- continuity and positivity towards future self after the intervention. *No significant change in vividness of the self or experienced similarity to the future self. Values remained stable at follow-up.* **Other:** Significant decrease in suicidal ideation, depressive symptoms and hopelessness (associated with improved scores on self-continuity, r= −0.67). No change for suicidal ideation and depression at follow-up, but increased hopelessness. | **Identity or related to identity:** impact on life-story, future thinking, positive experience. **Other:** Participant feedback was mostly positive: Intervention seems feasible and acceptable. 70% of participants completed the intervention. 4 participants suggested having more sessions and 2 suggested that the group should only be available for non-disruptive and stable participants. Generally, participants could understand the intervention well, but some participants had difficulty understanding the content or were disruptive in the groups. |
| Recovery narrative photovoice (Mizock et al., 2015)* | **Identity:** N/A  **Other:** *Non-significant findings for psychological well-being, empowerment and community integration.* | **Identity or related to identity:** impact on identity, empowerment and integration in the community. **Other:** Intervention seems feasible and acceptable: mean attendance of sessions was 6.9 sessions (out of 10). Majority of the patients made three or more photovoice works. |
| Self-concept group (Zahniser and Coursey 1995) | **Identity:** N/A  **Other:** Significant time-effect for functioning and symptoms (rated by case-managers). *Group differences for self-esteem, self-confidence, depression, observed functioning and symptoms were not significant. Self-esteem, self-confidence, and depression did not significantly change from pre- to post-intervention.* Participants' engagement with the sessions correlated only with self-esteem at follow-up. Participant’s response to feedback correlated with depression scores, but not with other outcomes. | **Identity or related to identity:** a majority of participants in both experimental and control groups mention improved self-confidence, self-esteem, social confidence, self-views. **Other:** Participant feedback was mostly positive. Some groups seemed to improve more on the functional outcomes and depression scores than other groups. |
| Self-concept/engulfment group (pilot) (McCay et al., 2006) | **Identity:** Significantly decreased engulfment at 3-month follow-up for the intervention group. *No significant change at follow-up for the control group.* **Other:** *Quality of life did not significantly change for the control group, nor for the intervention group* (except for the intrapsychic foundations subscale, which changed significantly for the intervention group). The intervention group showed a decrease in symptoms *(except negative symptoms), whereas there was no significant change for the control group.* | N/A |
| Self-concept/engulfment group (RCT) (McCay et al., 2007)** | **Identity:** Significant interaction effect for engulfment and significant decrease in engulfment for the intervention group, *compared to no significant effects in the control group. However, no significant interaction for self-concept (TSCS).*  **Other:** Significant interaction for quality of life and hope. Significantly increased quality of life and hope in the intervention group *(but not in the control group).* *No significant outcomes for self-esteem, self-efficacy, or perceived stigma.* | N/A |
| SELF (Self-concept and Engagement in LiFe) intervention (Konsztowicz et al., 2021) | **Identity:** Significant between group difference for engulfment after controlling for baseline (partial η2 = 0.15). Engulfment decreased for the intervention group (effect size: .48, significance not mentioned). Minimal clinically important difference (MCID) scores were partly met.  **Other:** *No significant group differences for maladaptive recovery style, self-esteem and self-stigma, depression or quality of life.* In the participant group, recovery style, self-esteem and internalized stigma improved (significance not explicitly mentioned, standardized effect sizes between 0.25 and 0.37). Depression scores only decreased in participants with a high depression score pre-intervention. | **Identity or related to identity:** The majority of the participants mentioned aspects of changed identity (e.g. roles, self-esteem, being more than the illness). **Other:** Other comments are related to: satisfaction (n=8), the therapist (N = 4), unhelpful aspects (n=1) and suggestions for improvement (N = 12, mainly about the wish to have more sessions). All items of the client satisfaction assessments were rated positive by at least 86 % of the participants. Negative responses to the items were mentioned by max 10.3%. There were 2 drop-outs, but these participants did not start the intervention. |
| Photographic Self-Image Confrontation (Spire, 1973) | **Identity:** N/A  **Other:** Differences on the draw-a-person test were significant. 90% of the participants showed changes in their drawings that were interpreted as positive by the therapist (e.g. body boundaries, drawings reflecting more positive aspects). Some scales of the hospital adjustment list and self adjective check list improved significantly. | **Identity or related to identity:** Recognition of positive characteristics was more difficult than the more negative characteristics. Reactions of the participants about becoming more self-conscious were mixed. **Other:** Generally, participants were interested in the intervention, reacted immediately to the photographs, and were often surprised by the photographs (as they did not often see themselves). One participant said she enjoyed the intervention and personal attention. Multiple participants reacted anxious/uncomfortable. One person did not seem to want to talk about herself and one person mentioned she wanted to stop. |
| Two different SET (self-experiential) treatments (Muzekari et al., 1973) | **Identity:** Significant interaction effect for self-concept. Note!: due to an undesired **decrease** in self-concept in the intervention groups, and unexpected increase in control group.   **Other:** There was a significant time-effect and group effect for symptoms (decrease over time). There was a significant time effect for the nurse observation scale (behavioral change), *but no significant interaction. Participants in the intervention groups declined on this scale (significance not mentioned).* After intervention all groups were able to recognize significantly more staff members and patients. | N/A |
| G4H (Groups for health) in the United Kingdom (Hogg et al., 2025) | **Identity:** Changes in social identity mapping variables were small and therefore no statistical analysis was performed.  **Identity-related:** *No significant group difference for* *in-group identification, and identity integration.*  Significant increase in in-group identification and identity integration from baseline to one-month follow-up.  **Other:** *No significant group difference for loneliness, wellbeing.* Significant decrease in loneliness and increase in wellbeing from baseline to one-month follow-up. Loneliness scores at all time-points were significantly different from each other. *No significant findings for* *perceived empathy.* | **Social identity mapping:** From session 2 – 5 the change in reported social groups was mostly less than 1 or slightly more than 1 (checked for: total number of groups, number of mental health-based groups, single groups, multiple person groups, generic groups, bespoke groups, high contact groups, supportive groups, positive groups). Mostly the change was in a positive direction. The number of supergroups increased and compatibility lines also reflected a positive change (especially a reduction of incompatibility lines in the group intervention participants). The number of positive groups increased slightly for both treatment arms, but the proportion of positive groups only increased for the group intervention.  **Other:** Sufficient retention and acceptability. The majority of the participants indicated they preferred their assigned treatment arm (group or individual). |

| ***B) Indirect SMI interventions: interventions indirectly targeting identity or without strong identity focus*** | | | | |  |
| --- | --- | --- | --- | --- | --- |
| **Intervention (reference)** | **Quantitative results** (underlined: significant, *italic: non significant,* normal text: significance not mentioned*)* | **Participant feedback and feasibility** | | |  |
| Selfgovernment group (Cerniglia et al., 1978) | **Identity:** *No group*time interaction effect for self-concept (*but there was a significant increase over time for all groups)  **Other:** Significant group*time effect for the hospital adjustment scale, with the experimental group scoring highest. *No significant findings for locus of control.* | N/A | | |  |
| Holistic hospital programme (Lukoff et al., 1986) | **Identity:** *No significant increase in self-concept for any of the groups (difference in change between groups was not significant)* **Other:** Significant improvement in physical fitness in the intervention group. *No significant findings for symptoms, nurses assessment, relapse, medication use or blood pressure.* | Few participants continued exercise and meditation after the program.  No harmful effects found of talking about hallucinations and psychotic experiences or of meditation. | | |  |
| Mindfulness-based Cognitive Therapy for Psychosis (Randal et al., 2015) | **Identity:** Significantly decreased difference between ideal self and self-as-recovered (d = 0,73), and unexpected tightening (increased similarity) of constructs (d = 0.79). *No significant increase in similarity between the element self and any of the other elements (ideal self, self as not coping, self as coper, self as recovered, main symptom). Non-significant increased salience of self (indication of prominence of self, d= 0.44).* **Other:** *No significant changes in clinical functioning, psychotic symptoms, beliefs about voices, core schemata of self and others. No significant improvements on the mindfulness scale* (except for the ‘act with awareness’ sub-scale (d = 0,49). Significant improvement in processes of recovery (d = 0.33). | 21 participants started with the intervention, of which 16 attended more than four sessions (completion rate 76%). | | |  |
| Dynamic cognitive intervention (Hadas-Lidor et al., 2001) | **Identity:** *No significant group differences for self-concept at either pre-intervention or post-intervention.*  **Other:** There were significant group-differences for cognitive ability (on several cognitive tests), as well as for work and residence status. *No significant differences for the iADL.* | N/A | | |  |
| Cognitive Behavioural Therapy for residual symptoms (Bradshaw and Roseborough, 2004) | **Identity:** Significant decrease in engulfment from pre-intervention to follow-up.   **Other:** Discontinuity between baseline and intervention scores for psychosocial functioning and symptoms. Improvements were statistically significant for 86% (mean effect size = 2,2) and 82% (mean effect size is - 2.07 of the participants respectively. Participants improved significantly in goal attainment. | Completers attended between 39 and 67 sessions. Satisfaction with the therapy (measured with the consumer satisfaction questionnaire) was high. | | |  |
| Transitional intervention to community based care (McCay et al., 2021) | **Identity:** *No significant pre-post intervention changes in engulfment. No significant changes from post-intervention to follow-up.*  **Other:** For the intervention group there was a significant improvement pre-post intervention in global functioning and in social/occupational functioning, whereas social functioning declined for the control group. Both the intervention and control group used less substances at post-intervention. *There were no significant changes from pre-intervention to mid-intervention. The improvement in self-esteem and QoL was not significant.* | Positive experiences with the intervention and effects on identity were reported in qualitative interviews (reported in detail in Table 2). | | |  |
| ***C. direct ABI interventions: interventions with a strong focus on identity and self-views*** | | | |  |  |
| **Intervention (reference)** | **Quantitative results** (underlined: significant, *italic: non significant,* normal text: significance not mentioned*)* | **Participant feedback and feasibility** | |  |  |
| Self-Concept group (Vickery et al., 2006) | **Identity:** Significant increase in positivity of the present self after the intervention. Specific items that changed significantly: perceived attractiveness, hopefulness, self-confidence, cooperativeness and boredom. | N/A | |  |  |
| Signature strengths intervention (part of positive psychology programme) (Andrewes et al., 2014) | **Identity:** *Non-significant findings from pre-intervention to post-intervention. A non-significant increase in present self-concept, whereas positivity of future self decreased (non-significant). The polarization (difference) between the present and the future self and between present and past self decreased (non-significant).* | All participants could mention some strengths (e.g. curiosity, love, spirituality and hope), and they could come up with something they could do with this strength in the following week. According to the therapists the participants the session about strengths was uplifting, acceptable, feasible, positively evaluated and enjoyable. However, extensive one-on-one support was needed for three participants. | |  |  |
| Therapeutic song writing (Baker et al., 2015)***** | **Identity:** Significant increased self-concept with medium effect size (d=0.557). The largest change was within the first six sessions. Self-concept changes significantly correlated with changes on several well-being and symptom measures. *Self-concept did not significantly correlate with outcomes of measures of flow and meaningfulness of the song writing, nor with suppression emotion regulation.* **Other:** Significant decrease in depression (d= 0.682). *Non-significant improvements in: negative affect, flourishing, satisfaction with life (small effect size, between .376 and .491). Non-significant improvements in anxiety, positive affect, re-appraisal, and suppression (very small effect size, all below d = 0.12). Strength of experienced flow did not correlate with other outcomes.* Experienced meaning of the song writing was significantly correlated with negative emotion outcomes (more meaning was related to more negative affect, anxiety and emotion suppression). | N/A | |  |  |
| Therapeutic song writing (Roddy et al., 2020) | **Identity:** Mixed outcomes on both measures. Head Injury Semantic Differential: increased in two participants (+12 and +86) and decreased in three participants (-16, -2, -17), Tennessee Self-Concept Scale: tendency to increase. Increase for four participants (+8 and +19, +1, +1) and remained the same for one participant.  **Other:** Engagement with the song writing and experienced meaningfulness of the process did not change much for two participants, whereas it increased for three participants. Wellbeing, distress and functional gains: mixed outcomes (within person and between person). In general, there were increases in functional gains. | The two participants who were most severely impaired showed in general more negative shifts in the outcomes (identity, wellbeing distress), but several other participants also showed some worsening of the outcomes, and no firm conclusions can be made. Authors observe a tendency for more positive identity and mood outcomes in participants who had less severe injuries and who improved more on the functional domain. | |  |  |
| Biographic–narrative intervention for aphasia (Corsten et al., 2015)*** | **Other:** Significant increase in health-related quality of life from pre-intervention to post-intervention (decreased complaints and burden, Cohen’s d between .25 and .27) and significant increase from post-intervention to 3-month follow-up. *No significant change in overall life satisfaction.* Some affective mood states changed significantly. After the last individual sessions the mood state ‘happy’ increased a little (Cohen’s d =.25), and ‘confused’ and ‘tense’ decreased moderately (Cohen’s d = .56 and .45). After the group intervention only the state ‘tired’ decreased significantly (Cohen’s d = .30). *The following did not change significantly: afraid, sad, angry, energetic.* | **Identity:** Positive experiences with the intervention and effects on identity were reported in qualitative interviews (reported in detail in Table 2). | |  |  |
| ***D. indirect ABI interventions: interventions indirectly targeting identity or without strong identity focus*** | | | | | |
| **Intervention (reference)** | **Quantitative results** (underlined: significant, *italic: non significant,* normal text: significance not mentioned*)* | **Participant feedback and feasibility** |  | | |
| Interpersonal process recall (communication skill training) (Helffenstein and Wechsler, 1982) | **Identity:** Significant larger increase in TSCS total score for the treatment group (and for the subscales: social and moral self).   **Other:** Sign. greater improvement for the therapy group on interpersonal relation ratings by staff and on ratings of behavior in interaction, as well as a statistically sign larger reduction in trait anxiety in the treatment group. *No significant differences in changes in interpersonal communication, state anxiety, and in a videotape analysis of interaction with unfamiliar other.* | N/A | | | |
| Physical exercise (Brinkman and Hoskins, 1979) | **Identity:** Mixed findings (on a 0.10 sign. level there was a sign. increase in identity, physical self, social self, and SVI self-concept, but there was also a significant "decrease in view or value of the personalities of others" and 'increased uncertainty in self-perception".   **Other:** Most participants showed changes in physical fitness (maximum oxygen uptake and lower heart rate response). Functional outcomes were mixed. Some individuals improved, e.g. walking speed, tie shoe laces, play golf, mowing lawn. But not all participants improved. | N/A | | | |
| MFG (Multifamily Group intervention) (Kelly et al., 2013)**** | **Identity:** Significant group-effect for self-concept: with healthy controls having higher self-concept scores than the participants. *Non-significant interaction effect for self-concept (effect size = 0.092), no significant time-effect.* **Other:** Significant group effect for mood, self-esteem and family functioning (higher in control group). *No significant interaction effects or time-effects for mood, self-esteem or family functioning* | N/A | | | |
| Recreational kayaking (Fines and Nichols, 1994) | **Identity:** Significant improvement from pre-intervention to post-intervention on all subscales of the self-concept measure.   **Other:** Significant changes in leisure satisfaction (all scales) and leisure attitude *(except for the cognitive scale).* | N/A | | | |
| Client-driven adjustment after ABI group (Von Mensenkampff et al., 2015) | **Other:** Significant correlation between the number of sessions and post-traumatic growth. | **Identity:** Positive experiences with the intervention and effects on identity were reported in qualitative interviews (reported in detail in Table 2). | | | |
| VaLiANT (Valued Living After  Neurological Trauma) (Sathananthan et al., 2025) ****** | **Identity:** Significant time-effect, *no significant interaction.* Minimal clinically important difference (MCID) scores were met at post-intervention and follow-up. From pre-intervention to follow-up the Chi-square analyses identified a greater proportions of reliable  change in the treatment group (significant at 0.1 level).  **Other:** Significant interaction effect for experiential avoidance, anxiety and negative post-traumatic growth. *No significant reliable changes or interaction effects for wellbeing, cognitive symptoms, cognitive strategy use, self-efficacy, participation, QoL, positive post-traumatic growth.* Mixed findings for mood scales and valued living scales, with some significant findings. | Low drop-out. Telehealth was only feasible with support and technical assistance. Treatment adherence and homework completion was high, and the intervention was acceptable. | | | |
| ***E) indirect ID interventions: interventions indirectly targeting identity or without strong identity focus*** | | | | | |
| **Intervention (reference)** | **Quantitative results** (underlined: significant, *italic: non significant,* normal text: significance not mentioned*)* | **Participant feedback and feasibility** | | | |
| Group mural painting project (Trzaska, 2012) | **Identity:** Significant interaction effect and post-intervention group difference for the social subscale (increase in the experimental group and decrease in the control group, no group-difference pre-intervention). There was also a significant group difference in the identity-scale post-intervention, *but no significant interaction for identity. No significant interaction effect for the other domains or total score.* | **Identity:** Scores on most of the validity scales of the TSCS were within a normal range, except for highly elevated scores on the ‘faking good scale’ which can indicate high social desirability of answers. Participants also did not understand all of the TSCS questions. | | | |
| *Further reading: See Mizock et al., 2014 for an analysis of the content of the photovoice works (not included in this review).  **In this study there was a significantly higher quality of life, self-efficacy, and functioning, and lower engulfment for dropouts (N=18), compared to completers (pre-treatment). ***Outcomes on quality of life and mood are reported elsewhere: Corsten, S., Konradi, J., Schimpf, E. J., Hardering, F., & Keilmann, A. (2013). Improving quality of life in aphasia—Evidence for the effectiveness of the biographic-narrative approach. *Aphasiology, 28*(4), 440–452. https://doi.org/10.1080/02687038.2013.843154 ****Note that qualitative analysis of the data is presented elsewhere: Couchman, G., McMahon, G., Kelly, A., & Ponsford, J. (2014). A new kind of normal: qualitative accounts of Multifamily Group Therapy for acquired brain injury. *Neuropsychological rehabilitation, 24*(6), 809–832. https://doi.org/10.1080/09602011.2014.912957.  *****Further reading: The authors wrote multiple articles about this intervention that did not fit with the inclusion criteria of the current review. For the song writing protocol, the reader is referred to: Tamplin, J., Baker, F. A., Macdonald, R. A. R., Roddy, C., & Rickard, N. S. (2015). A theoretical framework and therapeutic songwriting protocol to promote integration of self-concept in people with acquired neurological injuries. *Nordic Journal of Music Therapy, 25*(2), 111–133. <https://doi.org/10.1080/08098131.2015.1011208>  ******Note that qualitative analysis of the data is presented elsewhere: Sathananthan, N., Dimech-Betancourt, B., Morris, E., Vicendese, D., Knox, L., Gillanders, D., Das Nair, R., & Wong, D. (2022). A single-case experimental evaluation of a new group-based intervention to enhance adjustment to life with acquired brain injury: VaLiANT (valued living after neurological trauma). Neuropsychological rehabilitation, 32(8), 2170–2202. <https://doi.org/10.1080/09602011.2021.1971094> | | | | | |
