# Supplementary material for: Interventions targeting identity in adults with psychosis, severe mental illness, brain injury, or intellectual disability: a transdiagnostic scoping review
Source: Front Psychiatry. 2026 Feb 5;17:1674898. doi: 10.3389/fpsyt.2026.1674898 (PMC12916650; doi:10.3389/fpsyt.2026.1674898)
Supplement: Supplementary file 4 [file SupplementaryFile4.docx]

**Table 2: Qualitative findings**

| ***A) SMI: interventions with a strong focus on identity and self-views*** | | |  |
| --- | --- | --- | --- |
| **Intervention (reference)** | **Qualitative findings** |  |  |
| TIM (This Is Me) activity wheel (Van der Meer et al., 2021) | **Themes:** 1. usage, 2. effects, 3. evaluation, 4. implementation  **Key-points related to the themes:**  1. The tool was used in different ways, e.g. in groups, pairs, outside. Professionals often took the lead. Not all professionals wanted to share personal information. Reasons for not using the tool mainly included: health problems or need for more assistance.  2. Findings about identity were mixed. One person described a change in identity, but some did not perceive identity changes. The tool impacted: reflection about the past, changed/stronger relationship with the person they did the activity with/changed roles, getting more active or open for activity.  3. The tool was experienced by most participants as positive, fun, and easy to use. One participant did not find it easy to use. For some participants the first impression of the tool yielded anxiety.  4. Several barriers were mentioned, e.g. health problems, first impression (tool looks complicated), difficult to select an activity, availability of support staff. | |  |
| ***B) Indirect SMI interventions: interventions indirectly targeting identity or without strong identity focus*** | | |  |
| **Intervention (reference)** | **Qualitative findings** | |  |
| Transitional intervention to community based care (McCay et al., 2021) | **Themes:** 1. Welcoming support in the midst of experiencing fear and loss 2. Experiencing the transitional intervention 3. Impact of the transitional intervention   **Key-points related to the themes:** 1. Uncertainty about the future/ future care, they preferred to keep working with the supportive staff and felt positive about being able to keep working on goals together.  2. Recognizing they are not the only ones with challenges, value of being able to share experiences peers in the group. They trusted the coach, who was validating and action-oriented. Some participants gave examples of positive concrete actions and the support they got to work towards goals.  3. Valued sense of self and impact on agency and decreases in engulfment, goal attainment influenced self-confidence, increased hope and belief in recovery | |  |
| ***C) ABI: interventions with a strong focus on identity and self-views*** | | |  |
| **Intervention (reference)** | **Qualitative findings** | |  |
| My story project (Strong et al., 2018) | **Themes:** 1. More than a story: it changed my life; 2. Positive experience; 3. Hope; 4. Communication confidence   **Key-points related to the themes:** 1. The intervention had impact, was meaningful (e.g. thinking about the future, ´life came back to life´, feeling able to talk), and gave a new perspective (´ongoing growth´). E.g. through discussing negative experiences.  2. Fun and enjoyment, it was good to talk about the past, ´I can still make you laugh´ (they all told funny stories, to which the others reacted positively).  3. There is life after stroke, gaining confidence in themselves and their skills, not giving up, listening to others was impactful. One participant also mentions that sharing his story may have helped others.  4. After being shown their (increased) scores on a communication confidence scale patients were positively surprised, but unable to explain this increase. | |  |
| Woman’s self-help group with a focus on identity and feminity (Gelech et al., 2019) | **Identity work processes:** 1. I am able and valuable: constructing competent and gifted selves; 2. Things are not so bad: tempering the threat of loss and impairment; 3. I am not a child: resisting interpersonal infantilization and attributions of incompetence; 4. I am a woman: asserting a collective gender identity  **Key-points related to the themes:**  1. Positive characteristics, helping others, sharing knowledge, relabeling, downward comparisons, positive atmosphere, feeling recognized. Participants showed competence through statements and behavior.  2. Normalizing, sharing collective identity, humor (laughing about shared incapacities), focus on stable characteristics, post-injury gains. 3. Collective burden of infantilization, labelling this as ‘unjust’ was empowering.  4.Encouraging femininity, unique to this women-only setting: participants started talking about topics such as motherhood, sexuality and womanhood, attention for these gendered topics increased during the program. | |  |
| Online aphasia bibliotherapy group with a focus on discussing a book about adjustment and identity after stroke (Hoover et al., 2023) | **Themes:** 1. the book as mechanism for reflection; 2. learning in an adult and engaging environment 3. community and shared experience; 4. therapeutic environment  **Key points related to the themes:**  1.Similarities and differences with the narratives in the book impacted reflection and sense of self, empowerment, envisioning (future) activities/perspective. Participants should be ready for this as it can be scary/emotional or too early in recovery.  2. The topic was considered by most individuals as relevant, the book gave new insights, the psychological frameworks and content as empowering. Value of practicing reading skill, even though this can be difficult. One person did not find that the book gave new information.  3. Connecting with peers: sharing similar experiences, group helped to normalize, learning from each other’s perspective about the book, friendship and feeling accepted in a safe/supportive space.  4. Mostly positive reactions to the aphasia friendly and adult materials and to the facilitators, and some suggestions for improvement. | |  |
| Biographic–narrative intervention for aphasia (Corsten et al., 2015) | **Themes:** 1. Evaluation of individual narrative interviews; 2. Evaluation of the group sessions 3. Identity: agency, sense of control, doing things, disease concept  **Key-points related to the themes:** 1. Duration: time flew by; one-on-one interaction was positive (trust, being heard); Situation: well-prepared, sufficient time/patience, more depth than group session; Content: valuable. Mixed reactions about reflection on the past (some positive, but also confrontational).  2. Unfavorable time frame; researcher was supportive/patient; value of meeting like-minded individuals; successful communication; compared to the individual sessions, the value of being together and relaxed atmosphere were valued; positive effect of upward and downward social comparison.  3. Both group and individual sessions contributed to identity change. Participants felt a sense of competence, control, rediscovered meaningful activities (e.g. autonomous activities such as driving and social activities) and became less deficit-oriented. Examples of helpful processes include e.g. focus on abilities (past, communication), contributing, social comparison, community feeling. | |  |
| Therapeutic song writing in the United States (Strong and Sather, 2024) | **Themes:** 1. The process is a catalyst: 1.1 Relationship-centered experience, 1.2. Engagement in meaningful activities, 1.3. Identity exploration  **Key-points related to the themes:**  1. The whole intervention, not just the songwriting elements influenced outcomes.  1.1 Relationship-based care had a positive impact on the participants (e.g. someone who listens to you) and was valuable for the intervention process.  1.2. The intervention activities were meaningful and inspired reflection and engagement.  1.3. Songwriting enabled participants to reflect on their life and identity (past, present and future). | |  |
| ***D) Indirect ABI interventions: interventions indirectly targeting identity or without strong identity focus*** | | |  |
| **Intervention (reference)** | **Qualitative findings** | |  |
| Peer support adjustment group (Cutler et al., 2016) | **Themes:** Pre-group: 1. disrupted sense of self, Post-group: 2. Psychosocial adjustment; 3. Adapted sense of self  **Key-points related to the themes:** 1. Loss of identity (loss of roles; dependency and feeling inadequate); uncertainty about the future; isolation (not wanting to be a burden/changed roles/social places unavailable/feeling disconnected from others).  2. Motivation, self-confidence, validation, process of normalization (feeling ´not alone´ and ´understood´, collective identity, group members became friends), structure (new routine, feeling of purpose), safe place to share, verbalize and solve problems together, information sharing and new skills.  3. Reality check (comparison with group members with more impairments/decreasing self-pity/more belief in own capacity/self-reflection about strengths); feelings of purpose (helping others/contributing to the group); self-acceptance (hope/motivation/acceptance combined with self-determination/accepting illness, but not feeling defined by illness). | |  |
| Project-based intervention: developing and presenting an advocacy presentation (Hoepner et al, 2022) | **Themes:** 1. renewed sense of self; 2. positive impact on providers to care of future individuals with brain injuries; 3. rewarding and humbling; 4. being heard; 5. alternate personal narratives and identities; 6. improved clarity and conciseness of expression  **Key points related to the themes:**  1. From loss to new self: teaching and contributing to society; pride and self-worth; proud of collective accomplishment. One person: moved away from a focus on struggle and not feeling understood.  2. helping current and future providers understand the lived experience of ABI, foster empathy, raising awareness of dismissive communication while balancing positive and critical feedback, teach the speech-language student facilitators. 3. positive experience, appreciation of positive feedback  4. Being heard after being dismissed a long time.  5. Narrative: e.g. as teachers, advocates, experts6. speakers experienced communication improvement with each new presentation  *Fieldnotes: 5 participants first wanted to present, but then felt they could not speak for the whole group, e.g. because they had another duration of illness than the others. Self-efficacy fluctuated but overall presentations improved, participants felt the presentation was a success and narratives became less problem saturated. *Feedback: providers valued the presentation and learned from the lived experiences | |  |
| Client-driven adjustment after ABI group (Von Mensenkampff et al., 2015) | **Themes:** Pre-group: 1.Identity confusion; 2.What helped to cope; Post-group: 3.Normalizing emotion and behavior (N=17); 4.Acceptance and knowledge (N=14); 5.New identity (N=11); 6.Positive mental health (N=10).   **Key-points related to the themes:**  1. Confusion: struggle, insecurity, inferiority, feeling of disconnection because others don´t seem to understand the situation.  2. Factors that helped before the start of the group were: psycho-education and accepting (e.g. learning to do less), which is connected to motivation to change.  3. Connection/making friends: it was good to meet other people and group contact was normalizing. 4. Acknowledging the situation and adjusting to injury, self-acceptance, self-awareness and knowledge were helpful. 5. Anxiety diminished as participants gained self-confidence. Dealing with change: e.g. participants mention to feel ´the same but different´. 6.Positive feelings and expectations of life | |  |
| ***E) ID: interventions with a strong focus on identity and self-views*** | | |  |
| **Intervention (reference)** | **Qualitative findings** | |  |
| Narrative workshops for Mingle support group (Elderton et al., 2013) | **Observations:** participants shared stories about things they do not often talk about, life events and subjugated stories. They supported each other and experienced the group as a ´safe space´ to tell their stories, and "a gay place". Positive changes (e.g. confidence) were also noted by support workers. **Comparison of the before/after stories: after the sessions:** the participants mentioned other aspects of themselves than before, many of these aspects were positive. Interview feedback form: The intervention enabled hearing each other’s life stories, which impacted participants: e.g. they felt understood and included, recognized similarities, the stories touched them and they felt that life can get better. | |  |
