# Supplementary material for: Interventions targeting identity in adults with psychosis, severe mental illness, brain injury, or intellectual disability: a transdiagnostic scoping review
Source: Front Psychiatry. 2026 Feb 5;17:1674898. doi: 10.3389/fpsyt.2026.1674898 (PMC12916650; doi:10.3389/fpsyt.2026.1674898)
Supplement: Supplementary file 5 [file SupplementaryFile5.docx]

Supplementary Material 5 – Table 3 (General study characteristics), Table 4 (Intervention details, theoretical background), Table 5 (Barriers, facilitators, recommendations)

**Table 3**

*General study characteristics*

| **Intervention and country (reference)** | **Purpose of the study** | **Participant characteristics*** | **Study design    Time of assessment  Study notes**** | **Used identity outcome questions***** |
| --- | --- | --- | --- | --- |
| ***SMI: interventions with a strong focus on identity and self-views*** | | | | |
| Continuous Identity Cognitive Therapy in the United States (Sokol et al., 2021) | Evaluate the feasibility, acceptance, and effectiveness (on suicidal variables, mood, hopelessness and future self-continuity) of an intervention focused on continuous identity. | N = 17 veterans with SMI (schizophrenia, schizoaffective disorder, bipolar disorder or major depression), current or past partial hospitalization (mean age: 59.7, age range: 34-74, 17.6% women) | Quantitative single arm study (pilot/feasibility).   Pre- and post-intervention, follow-up after one month. | Future-Self Continuity Questionnaire (positive affect to future self, vividness of future self, similarity to future self, total score) to measure: “sense of persistence of personal identity from the present to the future”. |
| Recovery narrative photovoice in the United States (Mizock et al., 2015) | Describe narrative photovoice and evaluate the feasibility. | Per study N = 8 individuals with SMI, recruited from a psychosocial rehabilitation center (adults). | Two quantitative single arm studies, with different outcome measures (pilot).  Pre- and post-intervention | Only indirectly (questionnaire about a related topic (wellbeing), used as a proxy for identity and empowerment). |
| Self-concept group in the United States (Zahniser and Coursey 1995) | Evaluate the effectiveness (on self-concept, symptomatology and psychosocial functioning) of a self-concept intervention, and analyze relations with intervention response and engagement. | N = 81 individuals in the whole study (not mentioned how many in intervention group, but we expect half), in psychosocial rehabilitation 80% schizophrenia, schizoaffective disorder, bipolar disorder or major depression (mean age: 59.7, 57% women) | Quantitative study with active control group (psycho-education, with the majority of the participants, but not all, selected from a stratified random sample). Note: some statistical data (e.g. significance level) not reported.  Pre- and post-intervention, follow-up after one month. | Only indirectly (questionnaires about related topics (self-esteem and self-confidence), interpreted as measure of self-concept). |
| Self-concept/engulfment group (pilot) in Canada (McCay et al., 2006) | Evaluate the effectiveness (on self-concept - operationalized as engulfment-, and quality of life) of an intervention focused on self-concept and engulfment. | N = 26 individuals, < 35 years with first episode psychosis (mainly schizophrenia), who are within one year after start treatment in FEP clinic and had max. 8 weeks of antipsychotic medications before the study (mean age: 27, age SD: 6, 42,3% women) | Quantitative study with non-equivalent TAU comparison group (no direct comparison) (pilot).   Pre- and post-intervention, three-month follow-up. However, only follow-up outcomes reported in the article. | Modified Engulfment Scale. To measure: "the degree to which an individual’s self-concept becomes defined solely by illness". |
| Self-concept/engulfment group (RCT) in Canada (McCay et al., 2007) | Evaluate the effectiveness (on self-concept, self- esteem, self-efficacy, engulfment, self-stigmatization, quality of life and hope) of an intervention focused on self-concept and engulfment. | N = 29 individuals, < 35 years, with first episode psychosis (mainly schizophrenia), without significant medical illness, who are within two years after start treatment in FEP clinic and had max. 8 weeks of antipsychotic medications before the study (mean age: 25.07, age SD: 4.86, 31% women). | Quantitative study with random assignment to the intervention or to TAU (RCT).   Pre- and post-intervention. (Note: study is part of a longitudinal project, data on phase 2 of the RCT is (as far as we know) not yet published). | 1) Modified Engulfment Scale to measure "the degree to which illness defines an individual’s self-concept", and 2) Tennessee Self-Concept Scale (total score) to measure: "six external and three internal domains of self- concept.” |
| SELF (Self-concept and Engagement in LiFe) intervention in Canada (Konsztowicz et al., 2021) | Evaluate the feasibility, acceptability and effectiveness (on illness engulfment, recovery style, self-esteem, self-stigma, depressive symptomatology, and quality of life), of an intervention focused on illness engulfment. | N = 18 individuals with enduring schizophrenia and high engulfment scores on the MES (mainly schizophrenia). In care for psychosis, more than two years of antipsychotic medication and clinically stable/physically healthy (mean age: 39,6, age range 20-61; 55,6% women; mean IQ: 96.2, IQ range: 69-131. Note: 29 participants for within-group analysis). | Quantitative study with sequential assignment to the intervention or to TAU+ waitlist control (pseudo-randomized pilot).   Pre- and post-intervention (within one week post-intervention). | Modified Engulfment Scale, to measure: "the impact of schizophrenia on self-concept". |
| Photographic Self-Image Confrontation in the United States (Spire, 1973) | Describe the outcomes on behavioral measures, after an intervention focused on bodily self-perceptions and self-photography that aimed to change 'faulty self-concepts'. | N = 12; Age median (52,5); 0% male  N = 12 women with chronic schizophrenia, living in state mental institution. | Quantitative single arm study (participants purposefully selected).   Pre- and post-intervention | Only indirectly (Author uses the Draw a Person Test -participants draw themselves- and Adjective Checklist with traits, to check for behavioral changes related to self-views). Note that: the draw-a-person test was used in the 20th century, but its usefulness and validity has long been contested. The self-adjective checklist is also outdated (e.g. authors interpret changes in heterosexuality favorably). |
| TIM (This Is Me) activity wheel in the Netherlands (Van der Meer et al., 2021) | Describe the development of an activity tool that targets identity and describe outcomes of the pilot evaluation (e.g. effect on multidimensional identity). | N = 10 individuals with SMI, living in sheltered living (median age: 52.5, age range: 29-61, 60 women) | Qualitative study without control group (pilot, semi-structured interviews analyzed with thematic analysis).  Post-intervention | Semi-structured interviews about usage, effect, evaluation of intervention components, implementation, and effect on identity, e.g. questions about the effect of the tool on identity. |
| Two different SET (self-experiential) treatments in the United States (Muzekari et al., 1973) | Evaluate the effectiveness (e.g. on self-concept) of two experiential videotape feedback interventions focused on bodily self or emotion, and compare this to social interaction treatment. | In each condition 12 participants with chronic schizophrenia, who are hospitalized continuously for > 5 years (mean age: 54.1, age range: 33-66, 50% women) | Quantitative study with random assignment to one of the two experimental conditions or to a control condition (social interaction treatment).   Pre- and post-intervention (but picture naming test and nurse observation were mid-intervention). | Tennessee Self-Concept Scale (total score): to measure "overall level of self-esteem". |
| G4H (Groups for health) in the United Kingdom (Hogg et al., 2025) | Evaluate the feasibility and acceptability of a social-identity informed intervention targeting loneliness. Comparing the effectiveness of a group and individual format (loneliness, wellbeing, social identification and experienced empathy). | N = 16 individuals completed the follow-up after the individual therapy, and N = 14 the group therapy, with psychosis who feel lonely, are between 18-65 years old, and do not have a primary drug or alcohol diagnosis (mean age: 38.8/43.7, age range: 19-61, 58% women). | Quantitative pilot feasibility trial with random assignment to a group or individual format.  Social identity maps are made in session 2 and five, other measures within 2 weeks of the start of the trial, post-intervention, at 1 month follow-up and 6-month follow-up. | Social identity mapping (SIM): to assess social networks. Other outcomes did not assess multidimensional identity or self-concept but a specific identity aspect: in-group identification and identity integration. |
| ***SMI: interventions that less strongly target identity and self-views*** | | | | |
| Self-government group in the United States (Cerniglia et al., 1978) | Evaluate the effect of enabling participants to make their own choices (on self-concept, hospital adjustment and locus of control). | N = 18 individuals with schizophrenia who are living in custodial care homes for 0.5-12 years (mean age: 58.6, age range: 31-72, 50% women). | Quantitative study with two control groups (no intervention and comparable intervention during which staff made the decisions). Seven houses were selected and then allocated randomly to a condition.   Pre- and post-intervention. Note: duration of intervention is not mentioned. | Tennessee Self-Concept Scale (total positive score), to measure: "patient levels of self-concept". |
| Holistic hospital program in the United States (Lukoff et al., 1986) | Evaluate the effectiveness of the holistic intervention and test for differences with the comparison program. | N =14 individuals with schizophrenia, in hospital (0% women) | Quantitative study with random assignment to the intervention or to a program with a focus on social skills and family therapy.   Pre- and post-intervention | Tennessee Self-Concept Scale, to measure: "self-concept" |
| Mindfulness-based Cognitive Therapy for Psychosis in England (Randal et al., 2015) | Evaluate the effectiveness and feasibility of mindfulness-based cognitive therapy, and describe changes in personal construal (on self-esteem, salience of self, loosening of construing, coping and recovery). | N = 13 individuals with a psychotic disorder who are in care (mainly schizophrenia) (mean age: 37.6, age SD: 10.5, 15.4% women, note: 11 participants completed all repertory grids). | Quantitative single arm study.   Within three weeks pre- and post-intervention | A repertory grid is used to analyze the difference between constructs elicited by the participants based upon researcher-given elements (here: self, ideal self, self before psychosis, self as recovered from psychosis, self as coper, self as not coping, main symptom, person I care about, person I do not like) within a particular domain (self and others). This is used to: "explore changes in participants' opinions of the self, others and their experience of psychosis". |
| Dynamic cognitive intervention in Israel (Hadas-Lidor et al., 2001) | Evaluate the of instrumental enrichment therapy focused on cognitive functioning (on memory and thought processes, functional outcomes and self-concept). | N = 29 individuals with schizophrenia in a community day rehabilitation center, stable medication (mean age: 36, age range: 18-64, 44.8% women) | Quantitative study with TAU control group (matched pairs were randomly assigned).  Pre- and post-intervention, six-month follow-up | Tennessee Self-Concept Scale (total score), to measure "self-concept". |
| Cognitive Behavioral Therapy for residual symptoms in the United States (Bradshaw and Roseborough, 2004) | Evaluate the effectiveness of cognitive behavioral therapy adapted for participants with residual symptoms, (on symptoms, psychosocial functioning, goal attainment and self-concept, operationalized as engulfment). | N = 22 individuals with schizophrenia who have significant residual psychotic symptoms/functional impairment, and adhere to chlorpromazine treatment and don´t have a mental retardation (at least 6 months) (mean age: 30, age range: 26-46, 43% women) | Quantitative single arm study (multiple baseline). Note that baseline trendlines are provided only for measures of symptoms and psychosocial functioning  Pre- and post-intervention, with baseline at 6, 9 or 12 months (symptoms and psychosocial functioning were measured at more timepoints). | Modified Engulfment Scale, to measure: "the extent to which the self-concept is transformed by the experience of mental illness." |
| Transitional intervention to community-based care in Canada (McCay et al., 2021) | Evaluate the effectiveness of a multi-component recovery intervention for young adults who transition from early intervention to community-based care. | N = 15 individuals between 18-35 years of age in Early Psychosis Intervention for schizophrenia spectrum or psychotic disorder, ready for discharge within 2 months (mean age: 26, age SD: 4.5, 25.9% women) | Mixed methods study with TAU control group (prospective cohort design, semi-structured interviews analyzed with thematic analysis). Pre- mid-, and post-intervention and follow up after four weeks. | Modified Engulfment Scale to measure: "the degree to which illness defines an individual’s self-concept". + interviews about experiences and impact of the intervention. |
| ***ABI: interventions with a strong focus on identity and self-views*** | | | | |
| Self-Concept group in the United States (Vickery et al., 2006) | Evaluate the effectiveness (on self-concept) of a self-concept group. | N = 18 individuals with ABI who have adjustment difficulties (mainly TBI), and are inpatient in a subacute rehabilitation center (3–125 months post-injury) (mean age: 31,8, age range: 18-57, age SD: 12,1, 39% women) | Quantitative single arm study (pilot).   Pre- and post-intervention (beginning of first session and beginning of last session). | Head Injury Semantic Differential Scale (present self):   "Measure of self-concept that is based on the semantic differential paradigm", with "higher scores indicating a more positive view of self". |
| Biographic–narrative intervention for aphasia in Germany (Corsten et al., 2015) | Evaluate the effectiveness (on health-related quality of life and subjective well-being), and describe the impact on identity (as a possible working mechanism) of a combined narrative individual intervention and group discussion. | N = 27 individuals with more than 6 months aphasia (mostly due to CVA). Recruited from ambulatory rehabilitation or aphasia support groups and don´t have severe aphasia or depression (mean age: 60,85, age range: 44-73, age SD: 7,75, 45% women). | Mixed-methods study without control group (qualitative analysis of interviews with grounded theory).   Quantitative: within one week pre- and post- intervention, three-month follow-up. Qualitative: post-intervention. | Semi-structured interviews about e.g. the intervention and effect on emotions, daily life and social contact, self-views and expectations for the future, e.g. "Did the intervention affect your perspective on your past, present or future life?" and "What is now important to you? How do you see yourself now? What expectations do you have for the future?" |
| My story project in the United States (Strong et al., 2018) | Describe participant's experiences with and responses to a narrative storytelling intervention. | N = 3 individuals from aphasia support groups, who do not have severe aphasia (mean age: 61.3, age range: 55-66, age SD: 4.64, 0% women) | Qualitative study without control group (pilot, interview analyzed with interpretative phenomenological analysis).   Throughout (after each session) and one day post-intervention. | Interview in which participants reflect on statements about the intervention, communication confidence, and narrative (process), e.g. "Some individuals think that sharing stories is a way to help individuals think about who we are, where we are coming from, where we are going, and how the past, present, and future are meaningfully linked." and "Sharing my story gave me time to think about how my life has both changed and stayed the same with stroke and aphasia." |
| Signature strengths intervention (part of positive psychology program) in Scotland (Andrewes et al., 2014) | Evaluate the effectiveness (on self-concept) of a strengths-based exercise as a part of a positive psychology program. | Age M (38,3), SD (5,9); 100% male; IQ M (74,8), SD 10.55  N = 4 individuals with an ABI who showed challenging behavior (80% history of substance abuse) and severe or extremely severe injuries, in inpatient rehabilitation (mean age of all participants including non-completers: 38.3, age SD: 5.9, mean IQ: 74.8, IQ SD: 10.55, 0% women) | Quantitative single arm study (pilot).   Pre- and post-intervention   Note: Author describes that in the first week the adherence to the take home activity was poor, and that the intervention was therefore extended. It is not clear if adherence improved afterwards.  The intervention was in week 10-12 of a longer positive psychology program, results of which are not mentioned in this table. | Head Injury Semantic Differential Scale (past, present and future self): "Adjectives on the left hand-side of the scale denote a more negative self-concept, while adjectives on the right are more positive. Participants rated their sense of ‘self’ on each pair of adjectives, at three time points: in the ‘past’ (before brain injury), in the ‘present’ (during rehabilitation) and in the future (after rehabilitation)." |
| Therapeutic song writing in Australia (Baker et al., 2015) | Evaluate effectiveness (on wellbeing, self-concept and mechanisms: flow and meaning) and explore relationships between outcomes of the therapeutic songwriting program in which participants wrote songs about self. | N = 10 individuals with a spinal cord injury (N=5) or ABI (N=5), in inpatient rehabilitation, less than a year since onset (mean age: 38.9, age SD: 13.2, age range: 20-64, 10% women) | Quantitative single arm study (quasi-experimental)   Pre-, mid- and post-intervention (except for flow and meaning which were measured after each written song) | Head Injury Semantic Differential Scale (present self): "higher scores indicate a healthier, more positive, self-concept" |
| Therapeutic song writing in Australia (Roddy et al., 2020) | Describe changes and trends (e.g. changes in self-concept and wellbeing) during and after the therapeutic songwriting program, in which participants wrote songs about self. | N = 5 men with acquired brain injury (including: stroke, traumatic brain injury, and hypoxic brain injury, 31–322 days after injury), who were currently in subacute inpatient rehabilitation. No severe disturbances (emotional, cognitive, sensory, memory, language) (mean age: 40.8, age range: 29-51, age SD: 8.73, 0% women) | Descriptive case series analysis without control group (no statistical analysis of data).    Pre- and post-intervention (and mid-intervention for three participants) | 1. Head Injury Semantic Differential Scale, which: "uses self-rated responses on a continuum of contrasting adjective pairs (i.e., friendly–unfriendly) to measure a person’s perceived self-concept after head injury."  2. Tennessee Self-Concept Scale (total score), which: "conceptualises self-concept within multiple subdomains, namely physical, personal, moral, social, family, and academic self-concept". |
| Woman’s self-help group with a focus on identity and feminity in Canada (Gelech et al., 2019) | Describe the woman's self-help group, and describe identity processes related to the intervention activities, with special attention for the contribution of the gender perspective. | N = 5 women with moderate/severe brain injuries, more than 5 years after illness onset (mean age: 52,4, age range: 36-64; 100% women). | Qualitative study without control group (pilot, based upon observation, field notes and transcription of sessions, which are later connected to the activities through Sociolinguistic interactional analysis).   Throughout | Field notes and recordings, analyzed with a focus on participants presentation of self and identity work during the group meetings. |
| Online aphasia bibliotherapy group in the United States (Hoover et al., 2023) | Describe the experiences and impact of the aphasia book club about a book focusing on rebuilding identity after stroke. | N = 27 individuals with aphasia, recruited from university-based aphasia centers, several months to several years post-stroke (mean age of all participants in the intervention: 63, age range: 25-89, 50% women) | Qualitative study without control group (semi-structured interview, analyzed with reflexive thematic analysis, and social constructivist approach).   Post-intervention | Interview questions did not explicitly focus on identity, but on the experience of reading the book and participating in the therapy, e.g. "What was the impact of reading/discussing this book as a group?" |
| Therapeutic song writing in the United States (Strong and Sather, 2024) | Describe the experiences of participating in the songwriting project in which participants wrote songs about self. | N = 3 individuals above 30 years of age, 2-7 years after stroke, recruited from an aphasia support group. No severe auditory comprehension  deficits, deteriorating neurogenic disorders or behavior/psychiatric  problems (mean age: 64, age range: 50-77, 33% women). | Qualitative study without control group (semi-structured interview, analyzed with interpretative  phenomenological analysis)  Post-intervention. | Some interview questions focused on identity and others about the process or different life domains (e.g. from the A-FROM model). E.g. “Did participating in the ‘My Story’ project change how you think about yourself?”. And: “Was sharing your story helpful for you in thinking about your life?”. |
| ***ABI: interventions that less strongly target identity and self-views*** | | | | |
| Interpersonal process recall in the United States (Helffenstein and Wechsler, 1982) | Evaluate the effect of communication skill training through interpersonal process recall (on interpersonal and communication skills and anxiety and self-concept). | N = 8 individuals with non-progressive brain injury, who currently participate in a rehabilitation program, many within 2 years after injury (age range: 17-35, 19% women) | Quantitative study with control group (nontherapeutic attention).   Pre- and post-intervention, follow up for individuals who were still in rehabilitation after one month. | Tennessee Self-Concept Scale: to measure: "self-concept". |
| Physical exercise in the United States (Brinkman and Hoskins, 1979) | Evaluate the effect of a physical conditioning program on physical fitness and physical function and self-concept, and if these outcomes are related. | N = 7 individuals with CVA and hemiplegia, 8-54 months after discharge from the hospital (mean age: 43,71, age range: 22-59, age SD: 15.04, 71% women) | Quantitative single-arm study.   Pre-, mid- and post-intervention  Note that the authors used a 0.10 significance level. | Tennessee Self-Concept Scale and Social vocabulary index: "to assess self-concept (…). The TSCS was used because of its division of self-concept into separate components, such as physical self, personal self and identity. The SVI was used because of its vocabulary subscore, which was used to ensure subjects comprehension of the scale" |
| Client-driven adjustment after ABI group in Ireland (Von Mensenkampff et al., 2015) | Evaluate the effectiveness of a group that was client-driven and focused on adjustment to life and emotional wellbeing after brain injury. | N = 45 individuals with ABI who needed an intervention for adjustment to ABI and were selected after a psychological assessment. 25 participants with TBI, 20 other ABI (mean age: 40.45, age SD: 11.87, 28,89 % women) | Exploratory mixed methods study without control group (qualitative interviews analyzed with thematic analysis).   Quantitative and qualitative assessments: Pre- and post- intervention (during review session after six weeks).   Note: The authors mention that they also used the impact of event scale to evaluate subjective distress after trauma, but only for some participants. The outcomes of this scale have not been reported in the current article. Note also that the post-traumatic growth questionnaire was only used for 47% of the participants. | Interview questions about how participants perceive themselves and their strengths, and how this has been impacted by the injury and the intervention.   E.g. "How has your brain injury changed how you see yourself? How have your strengths helped you to cope with your brain injury? (...) Has participating in the group informed this process?" |
| Peer support adjustment group in Canada (Cutler et al., 2016) | Describe and understand the psychosocial adjustment process of people who attended the peer support group, and analyze this from the perspective of biographical disruption and repair. | N = 16 individuals with brain injury (mainly stroke, brain tumor or TBI) or other primary neurological diagnosis, recruited for the interview after they had participated in the past in the outpatient peer support group. Relatively high-functioning, living in the community (mean age: 41.4, age range: 23-54; 56,25% women). | Qualitative study without control group (semi-structured interviews analyzed with a directed content analysis with a descriptive-interpretive approach and from the perspective of Bury's framework of biographic repair).   One to six months post-intervention | Interview questions focused on psychosocial adjustment, experience of the intervention and social support. Adjustment in this study is explored through the lens of biographical repair (Bury, 1982). Therefore, the qualitative analysis indirectly touches upon identity as a topic. |
| MFG (Multifamily Group intervention) in Australia (Kelly et al., 2013) | Evaluate the effectiveness (on self-concept, and secondary: self-esteem, family functioning and mood) of a multifamily group intervention. | N = 41 individuals with an ABI, not currently in rehabilitation and on average 5 years after injury. Majority of the participants had severe traumatic brain injury after an accident, and did not live in a care facility (mean age: 39,36, age range: 18-73, age SD: 14.53, 29.3 % women). | Quantitative study with non-clinical control group matched on age and gender (pre-post-test trial).   Pre- and post-intervention | Tennessee Self-Concept Scale (total score) "to assess self-concept both globally and across a number of domains" |
| Recreational kayaking in Australia (Fines and Nichols, 1994) | Evaluate the effectiveness (on self-concept, leisure satisfaction and leisure attitude) of kayaking. | N = 8 individuals between 19-55 years who had a TBI after age 19, are discharged from rehabilitation for at least a year and have sufficiently high scores on a leisure motivation scale. | Quantitative single arm study (for which some participants were purposefully selected).   Pre- and post-intervention (one week after intervention). | Tennessee Self-Concept Scale (seven subscales: Total Positive Self, Physical Self, Social Self, Self Satisfaction Personal Self, Behavior and Identity), to measure an "individuals' evaluation of him/herself". |
| Project-based intervention: developing and presenting an advocacy presentation in the United States (Hoepner et al., 2022) | Describe how participants evaluated the successfulness of the project and describe indicators of shifts in personal narratives and identity. | N = 10 individuals with chronic mild to severe ABI (8 TBI, 1 stroke, 1 tumor) who had participated several months to multiple years in a community-based group (mean age: 47.2, age range: 24-68, age SD: 14.22; 40% women). | Qualitative study without control group (written feedback analyzed with interpretive description analysis).   Post-intervention | Questions did not explicitly focus on identity, but on the impact or value of the project to the participants, whether they experienced growth, the impact on listeners and evaluation of the presentation, e.g. "describe the impact or value of this advocacy project to you individually" |
| VaLiANT: Valued Living After  Neurological Trauma in Australia (Sathananthan et al., 2025) | Evaluate the feasibility and acceptability and the suitability of a larger trial; explore the effect of the intervention on different measures (on wellbeing, mood, cognitive symptoms, cognitive strategy use, experiential avoidance, valued living, self-efficacy, post-traumatic growth, participation, QoL, identity). | N = 35 individuals with ABI (57% CVA, 17% TBI, 14% tumor, 9% MS), at least > 3 months post-injury and on average 5 years post-injury, who experience cognitive or emotional difficulties impacting meaningful participation. No moderate-severe language deficits or severe cognitive disabilities, deteriorating neurogenic disorders or severe psychiatric illness (mean age: 49.6, age SD: 15.3; 43% women). | Quantitative pilot RCT with random assignment to the intervention or TAU waitlist control.  Pre- and post-intervention, and two-month follow-up. | Head Injury Semantic Differential Scale (present self) to measure self-identity. Note that this measure was included halfway through the trial. |
| ***ID: interventions with a strong focus on identity and self-views*** | | | | |
| Narrative workshops for Mingle support group in England (Elderton et al., 2013) | Describe and evaluate narrative workshops for the LGBT support group and the experiences of the participants with these workshops. | N = 11 individuals who participate in a LGBT support group for individuals with learning disabilities. Most participants had a mild or borderline learning disability (age range: 28-71, 9,09% women) | Qualitative study without control group (descriptive focus, evaluation based upon a comparison of session notes about narratives at the beginning and of the workshops and participant feedback).   Beginning and end of the workshop  Note: no specific qualitative or quantitative methodology is mentioned or used in this paper to analyze that data. | Comparisons of the stories told by the participants before and after the meetings. |
| ***ID: interventions that less strongly target identity and self-views*** | | | | |
| Group mural painting project in the United States (Trzaska, 2012) | Evaluate the impact of painting a mural together with the group on self-esteem (measured with a self-concept scale). | N = 10 individuals recruited from a private school, who have a high functioning developmental disability and cognitive difficulty caused by an intellectual disability, pervasive developmental disorder or traumatic brain injury (age range: 22-48; IQ range: 50-70, 60% mild intellectual disability, 60% women). | Quantitative study with a control group that got no treatment (non-randomized controlled trial).   Pre- and post-intervention (one day after intervention).   Note that some participants did not understand all questions from the self-concept scale. | Tennessee Self-Concept Scale (scales: physical, moral, personal, family, social, academic, identity and total scale), with the total score being used to measure self-esteem and the identity scale to reflect: "the basic belief as to who they see themselves as being". |
| **General characteristics of the subsample of participants from the intervention group who were analyzed in the paper (note: in some cases only information about the whole sample, including comparison group and drop-outs, was available and then this information is reported).  **Some general notes: for many of the quantitative studies the reliability of the scales has not been established. Reliability analyzes of the TSCS scale for the SMI population and ABI population have not been conducted. The FSCQ is not validated in target groups with a (severe) mental illness. Repertory grid techniques have been used more often with schizophrenia target groups, but this grid has been constructed by the authors, thus there is no information about reliability/validity. For interpretation of the studies, it is also relevant to mention that some studies took place more than 50 years ago and that the research context is substantially different (e.g. therapeutic approaches, use of language (e.g. 'schizophrenics'), photography was not widely available for each individual, some participants never saw themselves in a large mirror or photograph, not all participants may have gotten treatment).  *** FSCQ: Future-Self Continuity Questionnaire (Sokol & Serper, 2019); TSCS: Tennessee Self Concept Scale (Roid and Fitts, 1991 or Fitts and Warren, 1996), MES: Modified Engulfment Scale (McCay & Seeman, 1998); HISDS; Head Injury Semantic Differential (Tyerman & Humphrey, 1984); SIM: Social Identity Mapping (Cruwys et al., 2016).* | | | | |

**Table 4**

*Intervention details, theoretical background*

| **Intervention and country (reference)** | **Recipient, delivery** | **Duration** | **Aim of the intervention** | **Identity focus (how the authors frame identity in the paper)** | **Intervention procedures and topics discussed in the intervention** | **Suitable for participants with cognitive difficulties/impairments?** |
| --- | --- | --- | --- | --- | --- | --- |
| ***SMI: interventions with a strong focus on identity and self-views*** | | | | | | |
| Continuous Identity Cognitive Therapy in the United States (Sokol et al., 2021) | Group (6-12 participants)  In person led by the researcher and a therapist (master level). | 4 weeks: 4 meetings of 3 hours | The primary aim is of this intervention is to treat suicidal symptoms. A secondary aim is cross-temporal identity (continuity from past to present to future). | Focus on continuity of identity (sense of persistence, stability and connection from past, to present, to the future), positive and vivid future self, and self-narratives. | Topics: life story, values, possible futures, who you want to be in the future, present self, mindfulness, changing the life story  Materials: Worksheets (e.g. values identification worksheet, group values worksheet, life story traits worksheet)   Procedures: discussion, worksheets, visualization, meditation, metaphors. With a focus on the continuous identity, life values, connection to (positive, possible) future selves.   Main therapeutic approach fits best with: CBT, ACT, narrative therapy. | *Highly verbal: includes some abstract reflective exercises. Includes learning through doing: mindfulness/visualization.  *Feasibility: Most participants were able to understand the intervention and exercises independently. However, those with psychosis struggled with abstract discussions about identity, and individuals with severe depression had some initial difficulty with understanding. |
| Recovery narrative photovoice in the United States (Mizock et al., 2015) | Group (8 participants), family and friends could be invited to the presentation at the end of the intervention.  In person led by the researcher and a co-facilitator. | 10 weeks: 10 meetings of 2 hours | The aim is to make photographs and narratives, to guide recovery, positive sense of identity, empowerment and community integration. | Focus on recovery, empowerment and narrative.   Other key terms: positive /empowering self-narratives, reframing health problems, re-storying, liberating stories. | Topics: Recovery (e.g. stories, facilitators, and barriers), narratives (e.g. liberating stories, using different language), photovoice (e.g. sharing, giving feedback, community action, photovoice exhibit: three missions: “Who I Am”, “My Story,” and “My Recovery”)  Materials: Handouts, homework, camera and display of photo’s.   Procedures: Psychoeducation and exercises with a focus on identity, recovery and stigma. Participants also made photographs, which were presented during and a community exhibit where participants could give a speech.   Main therapeutic approach fits best with: photovoice, narrative therapy, recovery. | *Several verbal elements and reflective exercises. But mainly learning through doing: making photographs for the photovoice exhibition.  *According to the authors, photovoice may be useful for participants with cognitive difficulties or lower literacy.  *Feasibility: 82% of the participants were able to make at least one photovoice work. |
| Self-concept group in the United States (Zahniser and Coursey 1995) | Group   In person led by a staff-member of the rehabilitation center and a co-facilitator (psychology student) | 10 meetings of 1 hour | Focus on the 'personal domain': intervention mainly focuses on broadening self-concept and self-esteem. | Two main aspects:  1) self-esteem (positive self-evaluation) and 2) self-complexity (broadening self-concept) instead of role-constriction.   Other key terms e.g.: stigma, hopelessness, attitudes of close others, self-talk, strengths. | Topics: Identity, hope, strengths, praise, criticism, feedback, negative self-talk, handling mistakes. Mainly focused on the present. Main focus in the first sessions was identity, later strengths, and in the end a focus on self-esteem   Materials: -  Procedures: Introduction, psycho-education, group discussion or activity (e.g. role play), overview of the next session and closing activity. Self-esteem and positive feedback were encouraged. Participants learned to attribute negative aspects not to internal traits, make negative self-aspects less central and that incorporate (positive) self-aspects.  Group is described as structured and supportive. | *The intervention is largely discussion based and requires participants to be able to practice behavior (e.g. during role play).  *Feasibility or adaptability of the intervention for people with cognitive impairments is not explicitly mentioned. |
| Self-concept/engulfment group in Canada (McCay et al., 2006; 2007) | Group (+- 5 participants)  In person led by two clinicians | 12 weeks, 12 meetings of 1.5 hours | The aim is to develop a healthy self-concept (focus on self-stigma and engulfment). | Focus on counteracting engulfment (centrality of illness, stigma and role restriction).  Other key terms e.g.: healthy sense of self, meaningful life-goals, future hopes and acceptable illness-interpretation. | Topics: Engulfment, self-aspects, self-stigma, redefinition of self, interpretation of illness experience, future and life goals, and coping.  Materials: handouts  Procedures: Opening, discussion of last session, discussion of the group topics (with handouts) and open discussion, summary. Facilitators focus on: group processes (e.g. sharing, altruism) and reinforcing individual characteristics.  Main therapeutic approach fits best with: group psychotherapy | *Largely discussion-based (structured with handouts). *Feasibility or adaptability of the intervention for people with cognitive impairments is not explicitly mentioned. |
| SELF (Self-concept and Engagement in LiFe) intervention in Canada (Konsztowicz et al., 2021) | Individual  In person led by a therapist | On average 4.6 weeks: 4 meetings of 50 minutes | The aim is to counteract illness engulfment, and develop an identity beyond illness. | Focus on illness engulfment (illness centrality), and self beyond the patient role.   Other key terms e.g.: personhood (vs being a patient), self-awareness, roles beyond illness, narrative, internalized stigma. | Topics: Engulfment as a common coping strategy after psychosis, self-awareness and identity, narrative, how psychosis influences identity (constructive thinking about illness), self-esteem, and healthy self-concept.   Materials: e.g. homework exercises (e.g. bring a photo, bring a memory, describe yourself), handouts with session summary  Procedures: Opening with a check-in exercise (e.g. rating mood, character strengths), discussion of last session, discussion of the homework, group discussion, explanation of homework.   Main therapeutic approach fits best with: Mainly based on CBT principles, but also inspired by narrative therapy and positive psychology. | *Discussion-based (structured with session summary, participants made notes on paper). *Learning through non-verbal modalities: some of the activities were practical/non-verbal (e.g. finding a photograph).  *Note that the intervention was developed for clinically stable participants. People with a low IQ score or history of a medical/neurological condition that could affect cognition were excluded. |
| Photographic Self-Image Confrontation in the United States (Spire, 1973) | Individual   In person led by the author (psychiatric nurse clinician and treatment team leader) | 6 weeks:  12 meetings of 15 minutes | The content of the intervention is related to self-perception through self-image confrontation, with the aim to improve self-concepts. | Focus of the intervention is on self-perception.   The authors see self-concept as 'disturbed' (e.g. too high or low, highly influenced by others' views).  Other key terms e.g.: body image, self-image, self-awareness. | Topics: Self-perception and self-photographs.   Materials: Polaroid camera and pictures of the patients  Procedures: A photograph of the participant is made, followed by a short discussion about self-aspects and self-perception: do participants recognize the picture and how, what do they like or dislike, does the picture look similar to someone else, do they expect the pictures will change in the future?   Main therapeutic approach fits best with: not explicitly mentioned, facilitator is a psychiatric nurse. | *The intervention is rather passive, but the discussion is guided by relatively simple questions and non-verbal elements (photographs) are central in the intervention.  *Short sessions (15-minute) *Note that this intervention targets participants with a chronic illness who live in an institution. One participant was not able to recognize herself in the photographs. |
| TIM (This Is Me) activity wheel in the Netherlands (Van der Meer et al., 2021) | In duo´s (e.g. with another patient, or family, or caregiver)  In person activity | Aim was once every 2 weeks, but frequency varied from once to five times. | The aim is to develop or rediscover multidimensional self-identity. | Focus on multidimensional identity (broad range of self-characteristics).   The authors see identity as a combination of multiple interpersonal and intergroup identities.   Other key terms e.g.: unidimensional self-identity, illness-identity and stigmatized identity. | Topics: Leisure time, learning, music, significant other, characteristics, health, family, smell or taste, profession, house of the past, prejudice, happy place, happy things, talent, memories.  Materials: The ‘this is me’ picker wheel, which provides participants with suggestions for identity-related activities or exercises. There is also a ‘travelogue’ to log the activity (e.g. with whom they did the activity, how they experienced this, and space to put a photo or drawing of the activity). Other materials depend on the chosen activity (e.g. video, pictures, drawing, paper to write on, music, television show, sports material, music materials).   Procedures: Choose an activity (spin the wheel), do this in duo’s and log it in the travelogue (e.g. make a drawing, show or teach each other something, visit a place together).  Main therapeutic approach fits best with: Recovery movement. | *Designed to be easy to understand, with simple instructions. Participants can choose a relatively easy or more difficult activity. *Learning through non-verbal modalities and activities is central: e.g. listening to music, doing an activity together, smelling or eating, making a collage, and going somewhere. Participants can log their memories visually after the activity. *Developed through user-centered design in to fit with the needs of people with cognitive and communicative impairments.  *Note that If participants do the activity without a clinician, they may need to have some planning skills or be able to ask for help. |
| Two different SET (self-experiential) treatments in the United States (Muzekari et al., 1973) | Group (4 participants)  In person led by a group leader with college degree (control: mental health worker) | 6 weeks:  24 meetings of 1.5 hours | Self-concept changes through increased self-awareness of specific self-aspects (bodily self or emotions). | Focus of the intervention is on bodily awareness and emotion.  The authors see self-concept as ‘disturbed’ (e.g. self-awareness, bodily self-views) and focus the their writing on self-awareness, bodily disturbances, self-other distinction, integration of self-aspects (thoughts, emotion, physique, mannerisms, voice). | Topics: Physical condition: bodily awareness and distinction between self and other people. Emotional condition: emotion recognition and expression  Materials: videotape of the participants  Procedures: Focus on becoming aware of their emotions or bodily characteristics through watching videos of themselves. The role of the therapist was minimal and mostly instructive.   Main therapeutic approach fits best with: based upon theory about integrating self-aspects, but no (mainstream) therapeutic approach mentioned. | *Learning through non-verbal modalities: visual elements (videos) are central. *Discussion of these videos is mainly focused on awareness of self-aspects.  *Note that this intervention targets people who are hospitalized for several years. |
| G4H (Groups for health) in the United Kingdom (Hogg et al., 2025) | Group with 4-6 participants or individual.  In person by the researcher who was also a clinical psychologist; group-sessions were co-led with a lived experience researcher. | 2 months: 5 sessions + one informal check-in. Individual sessions had a duration of 60 minutes and groups of 120 minutes. | To diminish loneliness through supporting social identity. | Focus on social identity and social group membership vs loneliness.  Other key terms: positive social identity as someone with psychosis, integration of this identity with other identities, in-group identification, multiple social identities. | Topics: Social groups/social identity and importance on wellbeing, self-aspects, relation of the person with his/her social groups, compatibility of groups, benefits of social groups (social support, positive social identity, self-esteem), social goal setting, (self)stigma, discrimination, disclosure-decisions, conversational skills, psychosis-related barriers to social connection.  Materials: social identity mapping, social goal setting plan, session notes, homework.  Procedures: Psycho-education, group support, reflection, mapping their social network (social identity mapping), social goal setting, making a social plan/ and discussing this at follow-up, normalizing (symptoms, diagnosis, medication), social skills training.  Main therapeutic approach fits best with: social identity approach to health (SIAH). | *Learning through reflection and discussion, but also making concrete goals and group support.  *The intervention was adapted to be suitable for individuals with psychosis. Some adaptations are specifically made to help participants understand the material better, e.g. mailing session notes and homework, explanation of concepts (some individuals had difficulty understanding the concept ‘identity integration), giving examples of social identity maps, and more focus on discussion instead of writing in workbooks. Feasibility for individuals specifically with cognitive impairments is not explicitly mentioned. |
| ***SMI: interventions that less strongly target identity and self-views*** | | | | | | |
| Selfgovernment group in the United States (Cerniglia et al., 1978) | Group with +- 6 participants.   In person led by the senior author | Unknown number of weeks/sessions, meetings of 90-120 minutes once every 2 weeks. | To increase self-governance, personal responsibility and choice. | Focus on dependency vs decision making and influence on positive self.  Other key terms: incompetence and institutionalization (negatively evaluated in society) vs independence, positive self through shedding patient roles and taking responsibility. | Topics: Self-government, decision making about daily live in the care home.   Materials: none.   Procedures: All residents come together and talk about decisions that they want to make with regards to the daily life in the home, e.g. leisure, eating, work etc. The facilitator has an enabling and assisting role, but the participants make choices independently.   Main therapeutic approach fits best with: based on reality therapy and idea that patients need to take responsibility. | *The intervention is about decision making in a group, and participants are assisted in this process. Although feasibility or adaptability of the intervention for people with cognitive impairments is not explicitly mentioned, the intervention does seem feasible. |
| Holistic hospital program in the United States (Lukoff et al., 1986) | Group and family  In person led by multiple therapists: psychologists and a recreation therapist | 9 weeks: whole day program. | To reduce stress and increase positive beliefs about illness and expectations about recovery | Focus on growth and positive expectations.   Other key terms: growth, positive view of illness, wellbeing, self-love. | Topics: Physical exercise, meditation/mantra, stress education, positive beliefs (e.g. about brain harmony), growth and positive aspects of schizophrenia (e.g. examples of creative/well-known people with schizophrenia, mystical experiences), self-love and self-esteem, psychodynamic family therapy.  Materials: list with mantra's, soothing music, audio about stress, handouts, diagrams (e.g. to visualize 'brain harmony'), true/false quiz, rock music (examples of famous people who had schizophrenia), materials for art therapy and yoga  Procedures: physical exercise, yoga, meditation, stress psycho-education sessions, mobilizing positive beliefs sessions (e.g. make illustrations of brain harmony), growth and schizophrenia sessions (examples of well know people with psychosis, discussing mystical experiences or rituals, rock music), learning to love yourself session, family therapy  Main therapeutic approach fits best with: intervention inspired by holistic principles (e.g. meditation, stress, growth, exercise). | *Multiple components, of which it is not always clear how much they rely on verbal or reflective ability. Part of the program is activity/relaxation based, including sports, meditation, arts, and listening to music. The authors also mention using 'simplified diagrams and graphs'.  *Feasibility or adaptability of the intervention for people with cognitive impairments is not explicitly mentioned. |
| Mindfulness-based Cognitive Therapy for Psychosis in England (Randal et al., 2015) | Group  In person led by a therapist (trained in CBT and mindfulness) and researcher who was also trainee in clinical psychology. | 8 weeks: meetings of 2 hours. | The focus of the intervention is to practice MBCT: developing mindful awareness and learning to cope and relates to thoughts, feelings and behavior. | Intervention not clearly focused on identity. Identity is operationalized as personal construal of self and others (through repertory grids with a focus on: self, ideal self, self as coper and self as recovered. Self-esteem (distance between self and ideal self), salience of self). | Topics: meditation, coping, positive and negative events, thoughts and feelings, impact of experiences and psychosis on mood/behavior  Materials: handouts and cd with guided meditation exercises  Procedures: two or more meditation exercises (max 15 min) and cognitive therapy elements (e.g. discussion of positive/negative events diary).   Main therapeutic approach fits best with: MBCT is based upon mindfulness and cognitive therapy principles. | ***Learning through verbal elements (CBT) and mindfulness exercises.  *Feasibility or adaptability of the intervention for people with cognitive impairments is not explicitly mentioned. |
| Dynamic cognitive intervention in Israel (Hadas-Lidor et al., 2001) | Individual (with optional group)  In person led by occupational therapists trained in this therapy. | 1 year: 2 or 3 times per week, meetings of 1 hour (total: 100 hours). The optional group treatment was once in several weeks. | Instrumental enrichment focuses on cognitive ability and independence in daily life/occupation.  The extra group meetings aimed at communicative ability, group belonging and seeing problems from multiple viewpoints. | Intervention not clearly focused on identity. The authors use the TSCS to operationalize self-concept. They expect that learning skills can influence self-concept. | Topics: performance on cognition exercises and applying this to daily life.   Materials: 15 different types of pen-and-paper exercises (with modalities: graphic, verbal, paintings or numeric) focused on: organization, comparison/categorization, orientation in space, relations, social skills and integrative thinking.   Procedures: start with pen-and-paper cognitive exercises, followed by a discussion of the performance on the exercises and applying this knowledge or exercise to daily life.   Main therapeutic approach fits best with: cognitive training (cognition). | *Learning through pen/paper cognitive exercises: aim of the intervention is to train cognitive ability of participants with cognitive disabilities.  *Exercises can be adapted to need/ability. |
| Cognitive Behavioral Therapy for residual symptoms in the United States (Bradshaw and Roseborough, 2004) | Individual  In person led by licensed clinical social workers (master degree). | 1.5 year: sessions of one hour. In total completers got 39 to 67 sessions (mean: 50). | This CBT intervention focuses on residual symptoms and difficulties: symptoms, coping with stress/symptoms, negative thoughts, and self-esteem via self-appraisal training. | Focus of the intervention on identity is not clear, mainly focused on self-appraisal. For outcome measures, identity is operationalized as engulfment.   Key terms in the article are: role-loss, stigma, labeling and stress related to the illness impact the self-concept, and illness becomes central in this self-view, self-esteem, self-appraisal. | Topics: understanding of symptoms, thought stopping, distraction, vulnerability, stress, coping, relapse, changing behavior, maladaptive cognition, adaptive thoughts, self-esteem.   Materials: -  Procedures: CBT methods focusing on behavior and cognition. E.g.: treatment goals, behavioral skills, stress reduction (progressive relaxation, meditation, walking, planning activities), thought stopping/distraction, cognitive restructuring. Self-appraisal training was also used: making a daily list of positive events and a list of associated positive personal characteristics.   Main therapeutic approach fits best with: CBT, therapist with social work background. | *Relatively verbal intervention (CBT), but also learning through different exercises, some of which are activity-based and don't require a lot of verbal or cognitive capacity (e.g. relaxation, activity planning).  *Feasibility or adaptability of the intervention for people with cognitive impairments is not explicitly mentioned. Note that people with an intellectual disability or organic brain syndrome were excluded from the study. |
| Transitional intervention to community-based care in Canada (McCay et al., 2021) | Group and individual   In person coached by an occupational therapist | 12 weeks (4 week group + 8 week individual) | The aim is sustaining recovery after the early psychosis intervention program ended/during transition to community based care. | Focus on self-stigma and goals. The authors measure engulfment.   Other key terms: sense of self in youth with psychosis; self-esteem; self-reliance (through goal-setting). | Topics: dreams, hope, goals, barriers, self-stigma, engulfment, self-care, relationship, interpersonal skill, meaningful life goals, self-care, relational DBT skills, discharge readiness, goal-setting, community-based care (goal-based coaching)  Materials: worksheet to make a personal passport (about hope, goals, accomplishments and problem-solving plans).  Procedures: Week 1-4:  *weekly group discussion *individual: motivational interviewing, cognitive behavioral principles Week 5-12: working on concrete goals  Main therapeutic approach fits best with: skills training (DBT), motivational interviewing, CBT, and early psychosis intervention. | *The group intervention seems relatively verbal (discussion/skill-based), in individual sessions the participants were coached to take up activities in the community and work towards goals.  *Feasibility or adaptability of the intervention for people with cognitive impairments is not explicitly mentioned. |
| ***ABI: interventions with a strong focus on identity and self-views*** | | | | | | |
| Self-Concept group in the United States (Vickery et al., 2006) | Group (3-7 participants)  In person led by the author or a neuropsychology technician (master level) | 6 weeks: 6 meetings of 1 hour | Broaden self-concept, recognize positive self, increase self-knowledge and differentiate the importance of specific self-domains. | Self-concept is seen as one’s beliefs about functioning in different domains, which may be more negative after injury.   Other key terms e.g.: diminished self-concept, self-complexity, importance differentiation (importance of different self-aspects), self-esteem. | Topics: Self-concept, adjectives, pre-injury self, changes after life events, post-injury self-views, how changes in self-views (from before to after injury) impact identity and functioning, impact of negative self-concept, positive self, continuity between past self and post-injury self.   Materials: personalized folder with worksheets and a self-adjective questionnaire about themselves pre- and postinjury.  Procedures: Mainly group discussion of various self-aspects and identity changes. Facilitators mainly focus on self-complexity and differentiation of the importance of self-concept domains.   Main therapeutic approach fits best with: supportive and structured approaches. | *Discussion-based intervention, aided by multiple worksheet exercises.  *Intervention targets participants in subacute rehabilitation. Feasibility or adaptability of the intervention for people with cognitive impairments is not explicitly mentioned. |
| Biographic–narrative intervention for aphasia in Germany (Corsten et al., 2015) | Combination of individual and group (5-7 participants)   In person led by an adult education professional and speech-language therapist. Groups were co-led, individual meetings were one-on-one. | 10 weeks:  5 individual meetings and 7 group meetings of maximum 1.5 hours | The main aim is increased quality of life, through narrative identity development and ‘identity renegotiation’. | Identity renegotiation through narrative identity development and sharing one’s life story.   Other key terms e.g.: social interaction, meaningful life-story. Narratives can include impactful events, and can help to make sense of illness, find meaning and strengths and reconstruct identity. | Topics: Biographic narrative interviews: narrating about the life story and relevant life events. Group meetings: health and leisure topics were used to guide discussion about identity themes (roles, social interplay, coping with past, present and future).   Materials: communication aids (e.g. pictograms)   Procedures: During the individual biographic narrative interviews participants first talked about their life story and were later asked episodic questions about details. If needed participants could practice in these sessions how they could talk about this and use pictograms. During the group meetings the participants mostly discussed the topics and participants were supported to talk about themselves (e.g. through questions). The sessions were flexible (no structured agenda).   Main therapeutic approach fits best with: narrative /social construction and speech-language therapy. | *Mainly discussion-based: discussion-based group meetings and writing down the life story.  *Designed to be suitable for people with aphasia, including participants with severe language deficits, although people with severe auditory comprehension deficits were excluded.  *The facilitators supported participants to verbalize and use pictograms or write down what they wanted to say.  *Individual meetings were used to practice and to prepare for the group meetings. |
| My story project in the United States (Strong et al., 2018) | Mainly individual, but one group session. Family and friends could be invited to the presentation at the end of the intervention.  In person led by the author (speech-language therapist) | 5 individual meetings of 1.5 hour + a meeting before and after (mainly for testing) and one group meeting of 2.5 hours. | The aim was to co-construct a personal narrative. | Narrative identity: how telling life stories can shape identity discovery and sense making.   Other key terms e.g.: past, present, future. Making sense of who you are through meaningful story. | Topics: Constructing stories about: identity before ABI, stroke and aphasia, present identity, future and goals.  Materials: Booklet with tips and explanation of the intervention and a list with homework tasks (e.g. bring photo’s or items to help them tell the story). The story was recorded in PowerPoint, to allow for visual and auditive elements.   Procedures: Five individual sessions to construct and practice the story in collaboration with the facilitator, and one meeting in which the PowerPoint with the story was presented to the other participants, family and other guests.   Main therapeutic approach fits best with: narrative therapy (based upon McAdams, 2008), context of speech-language therapy. | *Mainly discussion-based (constructing/presenting a life story). As reminders of the sessions the participants were given visual hand-outs. Homework was written down.  *Designed to be suitable for people with mild or moderate aphasia, people with severe auditory comprehension deficits were excluded from the study.  *Learning through non-verbal abilities: Visual aids, photographs and physical items were brought to the sessions to aid the talking. The narrative story was presented in PowerPoint, to enable use of audiovisual elements. |
| Signature strengths intervention (part of positive psychology program) in Scotland (Andrewes et al., 2014) | Individual and take-home activity.   In person led by a facilitator and take-home (possibly facilitated by rehabilitation workers) | One individual session, which was part of a 12 weeks group program + two weeks of practicing and homework activity | To identify and focus on strengths, which is expected help participants reconnect to their values and goals and impact positive self-concept. | Positive and strengths-based self-concept and reduced difference between current self and pre-injury self.   Other key terms e.g.: strengths, values, valued daily activity. | Topics: strengths and values  Materials: brief strengths test to identify strengths and values, worksheet about using these strengths (in the past and present)  Procedures: Use of a signature strengths intervention, which took part after a group positive psychology intervention. The strengths intervention included filling in the strength test about strengths and values, focus daily on a specific strength, find (community) activities in which they use this strength and reflect upon their daily use of this strengths/activities.   Main therapeutic approach fits best with: positive psychology (as an approach to CBT). | *Mainly based on learning through a take home reflective exercise.  *The intervention was designed for participants with neurocognitive disabilities who had challenging behavior and severe injuries. Mean IQ scores of participants was 74,8.  *To be able to complete the intervention the participants highly relied on help by rehabilitation staff, but with this help the intervention was feasible and acceptable. However, the authors state that the intervention was 'extended (...) due to poor support and adherence." and also hypothesize that cognitive impairments may have hindered intervention uptake. It is not stated how the exercise is specifically adapted to the target group. |
| Therapeutic song writing in Australia (Baker et al., 2015) | Individual   In person led by a music therapist | 6 weeks:  12 meetings of 1 hour | The aim is to explore and integrate multiple self-narratives and domains of past, present and future self-concept. | The authors focus on past/present and future self-concept domains.   Other key terms e.g.: positive self, centrality of disabled self, congruence between past and present/future self, reappraisal after injury, alternative selves, integration of residual and injured self, fragmentation, self-narratives. | Topics: personal, social, family, physical, academic, and moral/spiritual self-aspects, in the present, past, and future.   Materials: audio recording and instrumentation  Procedures: Participants make three songs, one about the past, one about the present and one about the future.   Main therapeutic approach fits best with: narrative therapy, in the context of music therapy. | ***Songwriting is highly verbal, although musical elements are also central in the intervention.  *According to the authors, music interventions may be particularly suitable for participants with memory deficits, as music is supposed to help with memory consolidation and autobiographical memory retrieval.  *Note that participants in this study were required to have sufficient language abilities to participate. Feasibility or adaptability of the intervention for people with cognitive impairments is not explicitly mentioned. |
| Therapeutic song writing in Australia (Roddy et al., 2020) | Individual   In person led by a music therapist | 6 weeks:  12 meetings | The aim was to explore domains of self-concept and develop a balanced self-narrative. | See Baker et al., 2015. Identity viewed as multifaceted and in line with the HISDS and TSCS. | Topics, materials, procedures and main therapeutic approach: see Baker et al., 2015  In this article, the authors specifically mention that attention was paid to both positive and negative differences between past, present and future. The therapist facilitated the musical process, structuring of memories, and emotional processing. | *See Baker et al., 2015* |
| Woman’s self-help group with a focus on identity and femininity in Canada (Gelech et al., 2019) | Group (5 participants + staff + volunteers)  In person, together with female staff, volunteers and the 5 participants with brain injury | 10 meetings of 2.5 hours | The intervention aimed at wellbeing (social, emotional, psychological, sexual, and physical), capabilities and strengths, decentering disability, empowerment, femininity and identity. | The authors aim for "robust identities in the wake of injury".   Other key terms e.g.: social constructionism, validation, delegitimization, positive multifaceted identity that contains illness, femininity/being a woman, gender beliefs, empowerment. | Topics: Focus on the struggles and topics relevant for wellbeing of women with an ABI, e.g.: positive body image, relations, self-esteem, strengths, womanhood and femininity, growth, infantilization, positive self-descriptions.   Materials: needed for creative exercises, food sharing etc.  Procedures: Activities, formal discussion and casual contact: e.g. discussion about health and femininity, self-attribute reflective exercise, art, food sharing.   Main therapeutic approach fits best with: peer support, social constructionism, feminism. | *Learning through discussion (formal and informal) and through being together/doing leisure activities or creative exercises.  *Note that this intervention targeted participants who experienced significant cognitive and communicative impairments. As participants all struggled with disability, they recognized this in each other and started to share and recognize this and refer to these struggles with humor.  *Multiple support persons are present. Support persons tried to avoid being in a power position, and the approach was collaborative with a focus on equality, strengths and well-being activities/topics. |
| Online aphasia bibliotherapy group with a focus on discussing a book about adjustment and identity after stroke in the United States (Hoover et al., 2023) | Online group with 6-10 participants.   Led by students learning to become speech language practitioners. | 10 weeks, with meeting of either 75 or 90 minutes, depending on the setting. | The intervention combines group elements with bibliotherapy and reading a specific book to aim at: interaction, communication and self-reflection about identity reconstruction following stroke. | Adjustment and identity reconstruction after injury.   Other key terms e.g.: narrative construction through lived-experience stories, emotional recovery journey (frustration and grief over loss), coping with feeling as a failure when full clinical recovery is impossible, identity theft. | Topics: follow the topics in the book: stroke recovery narratives from multiple survivors; identity themes, including: frustration, grief, stroke as family illness, advocacy, relations, growth; lessons learned from the stories  Materials: book 'Identity theft: Rediscovering ourselves after stroke' (Meyerson & Zuckerman, 2019); Reading schedule. paragraph summaries, chapter highlights, discussion questions, PowerPoint, video conferencing.   Procedures: Between sessions participants read or listen to 2-3 chapters of the book, and these chapters are then discussed online in the group. Sometimes participants read out parts of the book. There was one 'meet the author' session.   Main therapeutic approach fits best with: Peer support, narrative reconstruction. | *Learning through reading and discussion.  *Note that this intervention targeted participants with aphasia and used aphasia friendly book summaries to help people read the book. Speech language trainees were present to help participants. Some people found it difficult to read the book.  *Feasibility or adaptability of the intervention for people with cognitive impairments is not explicitly mentioned. |
| Therapeutic song writing in the United States (Strong and Sather, 2024) | Mainly individual with support team, but one ‘release party’ with the group and invited guests such as family/friends.   In person led by a support team consisting of a music therapist, a clinician, a student (speech-language pathology) and researcher. | 12 sessions of 60 minutes + one release party with invited guests. | The aim was to integrate the self-concept. | The authors focus on identity through story-telling. They follow the same intervention protocol as Baker et al. (2015) and Roddy et al. (2020) which focuses on past, present and future self.  Other key terms e.g.: identity renegotiation, co-construction of narratives, identity exploration. | Topics, materials, main therapeutic approach and procedures fit best with: see Baker et al., 2015. The authors adapted this intervention for individuals with aphasia, e.g. writing what participants say, using visuals and added homework. They also added a 13^th^ session, which was a ‘release party’ with invited guests. | ***Songwriting is highly verbal, although musical elements are also central in the intervention. But this specific intervention was adapted to be suitable for individuals with aphasia: a support team was present each session, e.g. to help write and use supportive conversation.  *Participants could have severe aphasia, but not severe auditory comprehension deficits. |
| ***ABI: interventions that less strongly target identity and self-views*** | | | | | | |
| Interpersonal process recall (communication skill training) in the United States (Helffenstein and Wechsler, 1982) | Individual, interactions with different therapists (counselors, psychologists, speech pathologists, authors) | 20 days, 20 sessions of 60 minutes | The main aims are related to interpersonal- and communication skills. | Self-concept is measured with the TSCS, but not clearly a focus of the article and not focused on in the intervention. | Topics: communication skill and interpersonal functioning.   Materials: videotape and recorder  Procedures: Unstructured communication, then review of the videotape of interaction in which there was a deficient skill (communication dynamics and communication skill) and then practice of the skill.   Main therapeutic approach fits best with: cognitive retraining through skill-based communication training | *Mostly talk-based and focused on reflection on interpersonal communication. Visual video feedback is used to help participants remember the situation that is under discussion. *Feasibility or adaptability of the intervention for people with cognitive impairments is not explicitly mentioned, but the authors state that the intervention is suitable for people with mild/moderate disability. Participants with a lot of deficits were excluded. |
| Physical exercise in the United States (Brinkman and Hoskins, 1979) | Individual | 12 weeks: 36 sessions of 30 minutes (three times per week) | The main focus is on physical fitness | Perception of self (including body image and self-esteem) and the impact of self-perception on behavior and attitude ("attitudes and behavior are constructed by the individual to preserve or be consistent with his self-concept") and goal attainment. | Topics: no topics, only exercise  Materials: stationary bicycle. Heart rate/blood pressure measurements.   Procedures: Physical exercise (intensity depending on heart rate and blood pressure).   Main therapeutic approach fits best with: physical therapy | *The intervention is physical. Individuals learned through experiencing changes and abilities.  *Feasibility or adaptability of the intervention for people with cognitive impairments is not explicitly mentioned. Individuals with receptive aphasia or alexia were excluded. |
| Client-driven adjustment after ABI group in Ireland (Von Mensenkampff et al., 2015) | Group (8-10 participants)  In person, driven by clients, but facilitated by two clinical psychologists and an assistant psychologist. | 6 weeks: 6  meetings of 2 hours + 1 7th 'review meeting' after 6 weeks. | Aims to assist with adjustment to life with brain injury, emotional wellbeing and learning new psychological skills needed in the rehabilitation process. Identity is one of several topics and not a very explicit aim. | Not specified, but identity is mentioned in the context of psychosocial adjustment.   Other key terms e.g.: adjusting and coping with injury, self-esteem, emotion. | Topics: topics related to adjustment, e.g. coping, self-awareness, communication, acceptance, social skill, identity.   Materials: -  Procedures: Flexible group discussion. Clients could direct the meetings, but some topics were generated and introduced by the psychologists.   Main therapeutic approach fits best with: Psychological therapy in rehabilitation context. Main therapeutic approach fits best with: of the psychologist is not mentioned. | *The intervention is mainly talk-based, and authors do not mention any activity-component. *Note that a psychologist assessed participants’ ability to participate in therapy before inclusion in the study. Feasibility or adaptability of the intervention for people with cognitive impairments is not explicitly mentioned. |
| Peer support adjustment group  in Canada (Cutler et al., 2016) | Group (on average 7 participants per group and maximum 10)  In person, led by an ambulatory care social worker. | 16 weeks: 8 meetings of 1.5 hour. | Psychosocial adjustment in the transition period to home. | How the authors understand self-identity is not specified. The focus of the analysis is on biographical disruption and repair (Bury’s framework): the sudden and disruptive experience of brain injury affects the future, possibilities and outlooks on normative life circumstances, and sense of self. | Topics: e.g. adjustment, emotion, self-esteem, self-image, relations, sexuality, coping with stress, planning, getting active again, vocation.   Materials: session schedule, handouts with summary, worksheets, visual aids (e.g. whiteboard).   Procedures: In the introductory session participants talked about the topic-list. Each group started with a check-in and discussion about the last session, followed by a discussion about a topic (need-based/flexible), group exercises, brainstorm, psycho-education or a talk by a guest-speaker. The social worker was attentive to cognitive and behavioral difficulties (e.g. fatigue, reminders for the meetings, need for repetition).   Main therapeutic approach fits best with: Led by a social worker. Intervention was analyzed from a sociological perspective, unclear whether the intervention is also based upon this theoretical background. | *Exercises were used to encourage learning, but the intervention was mainly discussion-based. The facilitator structured these discussions, for example by summarizing and repeating what participants said. *The facilitators were attuned to participants’ difficulties with communication and memory, as well as their levels of tiredness. Visual aids (flip-over) and memory aids (take-home handouts and session schedules written in a large font) were used. Facilitators reminded participants about the planned sessions beforehand.  ***Note that the intervention targets relatively high-functioning participants. |
| MFG (Multifamily Group intervention) in Australia (Kelly et al., 2013) | Group with 6-8 families (including: person with brain injury and caregivers, e.g.: parents, spouses, siblings, children or friends)  In person, led by a clinical psychologist (trained in MFG) and clinical neuropsychologist. | 12 weeks: the 2 education sessions were 2 hours, the 2 group sessions in which they described themselves were 1.5 hour. Duration of the other meetings is not mentioned. | Intervention seems to focus mainly on supporting families with problem solving and adjusting to psychosocial problems. Identity is a small subgoal, however the authors did expect an impact on self-concept, as well as family functioning, self-esteem and mood. | Who are you, with the TBI and beyond the TBI?  Self-concept is defined in line with the TSCS as the total of self-evaluations on different domains (family, personal, social, physical, work and moral self-concept). | Topics: Description of who you are (with and beyond TBI); daily problems (e.g. sleep; gambling; isolation; anger; grief/loss; sadness; memory) and psycho-education (impact of ABI, coping)   Materials: Whiteboard, memory aids (photo's, workbooks/take-home sheets)  Procedures: 2 sessions of group psycho-education, 2 sessions to describe themselves to the group, other sessions focused on problem solving and were mainly solution focused. Participants were asked to talk mostly about their own experience.   Main therapeutic approach fits best with: multifamily group therapy | *The intervention is described as 'inclusive' as it uses memory aids/whiteboard and asks each person to talk about their own experience. Handouts were also provided. The intervention seems mainly discussion-based.  *The authors mention that they think that the participants may have forgotten what they learned in the sessions or could not apply it in daily life due to the cognitive impairments. |
| Recreational kayaking in Australia (Fines and Nichols, 1994) | Group  Not mentioned who led the group. | 12 weeks: 12 times kayaking for 1-1.5 hour. | Physical interventions are used to target physical and psychological wellbeing.  Based-upon the literature the authors also expect that this type of physical activity may indirectly impact self-concept, leisure satisfaction and leisure attitude. | Identity as "his/her perceptions of self”.   The TSCS is used to explain self-concept as a summary of self-evaluations on multiple domains (e.g. physical, social, personal, behavior, self-satisfaction and a total score reflecting self-esteem). | Topics: no discussion/topics, only kayak-activity  Materials: kayak  Procedures: physical activity (kayaking)  Main therapeutic approach fits best with: Recreational professionals/active recreation. | *Activity-based: kayaking. The intervention does not require active reflection or discussion. |
| Project-based intervention: developing and presenting an advocacy presentation in the United States (Hoepner et al, 2022) | Group project with 12 people involved. Participants presented at conferences and for peers/family.  Project initiated by the participants and co-facilitated by three speech-language pathology students | 6 months. Everyone: sessions to prepare the content/story (total 15 hour). presenters: additionally 8*1 hour sessions to practice presentation + 5 hours practice presentations online. For the people doing the video: one hour for the video recording. All participants watched the video´s. | To aim was to develop and deliver an advocacy presentation to educate clinicians about lived experience of (chronic) ABI. An indirect aim of the project was to redevelop new narratives. | Sense of self can change through narrative reconstruction and activities that are meaningful and impact others.   Other key terms e.g.: Problem-saturated narratives focused on illness versus renewed sense of self, overcoming difficulties, narrative reconstruction, expert role, contributing to society, self-efficacy. | Topics: lived experiences of (chronic) ABI, coping with impairment and overcoming challenge + experiences with services and community based groups, presenting.   Materials: Video conferencing materials, materials to make a video and presentation.   Procedures: Project-based intervention focused on developing an educational advocacy presentation for care providers. Facilitators used motivational interviewing and narrative principles aimed and alternative thick narratives, and peers gave feedback and discussed the content of the presentation. There were practice sessions, a session to make video´s, and a final academic grand rounds presentation.   Main therapeutic approach fits best with: narrative therapy, project-based interventions, motivational interviewing. | *Learning through discussion and practicing a presentation *Note that this intervention targeted participants with mild to severe chronic ABI.  *Feasibility or adaptability of the intervention for people with cognitive impairments is not explicitly mentioned. |
| VaLiANT: Valued Living After  Neurological Trauma in Australia (Sathananthan et al., 2025) | Group (3-8 participants), family and friends could be invited to the last session.  In person, telehealth or blended depending on COVID-19 restrictions, facilitated by two trainee psychologists and a clinical neuropsychologist | 8 weeks: 8 meetings of 2 hours. | To improve wellbeing and adjustment after ABI, focusing on meaning making, valued and meaningful engagement in life, managing cognitive and emotional barriers to valued activities. | Self-identity is seen as one aspect of adjustment to ABI, and identity loss is seen as a possible barrier to meaningful participation. Identity is not a main focus, but is measured with the HISD. | Topics: life domains, work/study, leisure, relationships, health, values, goal-setting, barriers to valued living (cognitive,  emotional, health, social), dealing with barriers (ACT, compensatory strategies).  Materials: x  Procedures: Explore values through exercises, develop a ´valued living´ plan including one SMART-goal, identifying barriers to the valued activities, psycho-education about specific strategies and practicing strategies (e.g. cognitive and psychological such as self-compassion or strategies from ACT), group discussion, homework to practice the strategies and do the valued actions from the plan. In one session about relationships close others were invited.  Main therapeutic approach fits best with: cognitive rehabilitation and ACT**.** | *Learning through discussion, exercises and concrete actions/homework practice.  *Participants had an average IQ, most had some cognitive impairments.  * Technical support was needed for the online delivery, especially for those individuals with more impairments. |
| ***ID: interventions with a strong focus on identity and self-views*** | | | | | | |
| Narrative workshops for Mingle support group in England (Elderton et al., 2013) | Group (9-11 participants who were all part of the same support group for people with ID who identify as LGBT + presence of staff members), participants could optionally present at a conference.   In person, led by two facilitators, and supported by a clinical psychologist and staff. | 4 workshops of 1.5 hours (within 5 months) | Workshops aim to help participants strengthen and develop self-identity: through telling their narratives, develop positive stories that are not problem focused, discover subjugated and alternative stories and share these in a group. | Identity can develop through construction of stories.   Other key terms e.g.: positive identity, alternative stories, problem-focused/negative stories, subjugated stories about hope, sexual identity, capacities. | Topics: Group storyline, personal life story  Materials: E.g. photographs, drawings and templates. For example, a timeline of the group was made with photographs (of members and locations). Templates, e.g. a storybook template (with elements such as: title, begin, middle, end) and a certificate template (qualities, things other people value about them, what they do and can do with these capacities).   Procedures: Opening, ice breaker, good news round, narrative workshop (open group). 1. discussion about the story of the group, 2. work individually to make a personal lifeline (with positive and negative events), 3. storybook template (individual exercise) and possibility to share this with the group. Facilitators used questions to ´externalize conversations´. 4. Sharing stories, template with positive characteristics. Additionally: preparing together for a sexuality conference where some participants went to share stories about gender, homophobia and sexuality.   Main therapeutic approach fits best with: narrative therapy. | *This intervention was tailored to people with intellectual disabilities. Participants learned through discussion, creative exercises and worksheets. For example, participants used photographs and drawings, and worksheets allowed for visual overviews (e.g. making a ‘certificate’ with a summary of their qualities).  *Several support persons were present and helped participants with the individual exercises. Facilitators tried to help participants feel secure, for example by inviting support staff that the participants knew already and by joining a group meeting before the start of the workshops. |
| ***ID: interventions that less strongly target identity and self-views*** | | | | | | |
| Group mural painting project in the United States (Trzaska, 2012) | Group (10 participants), family and friends could be invited to the presentation at the end of the intervention.   Led by a facilitator (not specified). | 4 weeks: 2 meetings per week, 1 hour per meeting.  One extra 9th) meeting was planned because participants needed some extra time to paint the mural. | The aim of the intervention is to give participants a positive experience, which could counteract learned helplessness and impact self-esteem/self-worth (measured with a self-concept scale) | Reinforcing creativity: may impact self-esteem and learned helplessness.  Self-concept and identity are only explicitly mentioned in the context of the TSCS outcome measure. | Topics: discussion related to the feelings/interpretation of the painting and the group processes.   Materials: acrylics, brushes, mural paper, canvas, paper, markers, pencils/eraser, T-square.  Procedures: participants design and create a mural painting, they brainstorm and decide about a painting together, try materials out and are stimulated to reflect about their feelings, role in the group and the process. After a check-in, they work for 40 minutes and then talk about the process and clean. The therapist reinforced social interaction, discussion related to the paintings (e.g. feelings, interpretation) and appropriate use of the materials.  Main therapeutic approach fits best with: art therapy. | *This intervention was tailored to people with intellectual disabilities. The intervention was largely activity-based (painting a mural). But participants were also stimulated to reflect upon this activity.  *The facilitator helped participants, for example by giving them time to practice with the materials. |

**Table 5**

*Barriers, Facilitators, Recommendations*

| **Intervention and country (reference)** | **Barriers** **authors' hypothesis;  **based upon observations/feedback* | **Facilitators** **authors' hypothesis;  **based upon observations/feedback* | **Recommendations** **authors' suggestions for adaptations of the intervention **participants feedback* |
| --- | --- | --- | --- |
| ***SMI: interventions with a strong focus on identity and self-views*** | | | |
| Continuous Identity Cognitive Therapy in the United States (Sokol et al., 2021) | *Participant characteristics***: difficulty understanding abstract topics (psychosis) or initial sessions (severe depression); some participants were disruptive or not treatment ready. | *Intervention characteristics***: participants (after pilot) indicated they wanted three-hour sessions; attention for meaningful life-story and positive self: impacted views on illness(-identity)  *Possible mechanisms**:* positivity towards the future self (correlated with diminished suicidality, hopelessness and depression).   *Group format*:* may have helped with attaining goals, but individual version may be more tailored. | *Duration***: more sessions, reunions (monthly)  *Participant-characteristics***: select only participants who are treatment-ready and stable  *Format*:* group seems helpful, but individual intervention may be more personalized.   *Content*:* more attention for homework (concrete, discussing at session start), discussing other suicide-related topics (e.g. interpersonal difficulties). Unclear if there was enough focus on vividness of future self and similarity to future self, however, it is also possible that positivity toward the future is a more important factor.   *Extending target group**: may be relevant for other target groups with high suicidal ideation. |
| Recovery narrative photovoice in the United States (Mizock et al., 2015) | *Not mentioned* | *Intervention characteristics**:* participants were engaged in the process and photovoice works included identity themes, thus it may have helped participants to reflect about their identity. | *No explicit recommendations* |
| Self-concept group in the United States (Zahniser and Coursey 1995) | *Emotional impact***: after discussion of negative topics (remaining positive self-views in difficult times/dealing with criticism)  *Duration**: more time/repetition may be needed to practice. | *Positive emotion***: after strengths-based exercise and positive feedback  *Group characteristics**:* group chemistry/cohesion effect (may have had more effect than intervention condition) | *Duration**:* more sessions, more time needed to practice behavior multiple times  *Participant-characteristics**: attention for group composition (due to the impact of group cohesiveness)  *Format*:* include some individual sessions, helping each other outside of the sessions could also be facilitated or encouraged.   *Content*:* discuss with participants before the start what they expect and what their goals are. Begin and end sessions with a positive note, use humor and reinforcement, especially when the topics are negative. |
| Self-concept/engulfment group in Canada (McCay et al., 2006) | *See McCay et al., 2007* | *Group format*:* may impact adherence and is promising for identity-interventions | *No explicit recommendations* |
| Self-concept/engulfment group in Canada (McCay et al., 2007) | *Participant characteristics***: drop-outs had lower engulfment, participants with low engulfment may be more difficult to engage in therapy. | *Intervention characteristics*:* focus on engulfment/self-stigma (may impact hope and quality of life), hope, individual characteristics and group processes.  *Possible mechanisms*:* initial changes in engulfment may cause other changes in the long run (e.g. self-concept). *Timing*:* attention for engulfment may be especially important and relevant early in the course of illness. | *No explicit recommendations* |
| SELF (Self-concept and Engagement in LiFe) intervention in Canada (Konsztowicz et al., 2021) | *Duration*:* longer intervention may be needed if aim is also to change depressive symptoms and quality of life, but could impact recruitment/drop-out. Participants asked for more sessions. | *Intervention characteristics**: focus on non-patient roles may protect against self-stigma. Different elements (e.g. constructive views on identity, healthy self-concept, self beyond illness) may together have a ‘synergistic effect’. | *Duration**:* more sessions (n=10 participants, but authors note that this may negatively influence participation rates) *Format*:* delivered online or by peers  *Content**:* e.g. discussing stereotypes, more attention for homework tasks, focus less on illness, focus on labels and discuss how family, friends and therapist approach a person, more attention for deepening aspects and feelings.   *Extending target group*:* intervention may also be relevant for other target groups |
| Photographic Self-Image Confrontation in the United States (Spire, 1973) | *Participant characteristics***: one participant was not able to recognize herself.  *Disliking the intervention**:* some participants did not want to talk about themselves or did not like becoming more self-aware.   *Emotional impact***: intervention may provoke anxiety for some participants. | *Intervention characteristics*:* photographs of the participants can help with therapeutic relationship and as a start for therapeutic exploration. | *No explicit recommendations* |
| TIM (This Is Me) activity wheel in the Netherlands (Van der Meer et al., 2021) | *Intervention characteristics**:*  verbal elements may be confusing or unnecessary. One person found the intervention not easy to use. Some feedback on the usefulness of the travelogue that was used to log experiences.   *Participant characteristics**:* health issues, difficulties with planning, choosing an activity, asking for help, reflecting, understanding intervention, getting active. Intervention may not be for everyone.   *Emotional impact**:* leaving the institution/first impression of the intervention may provoke anxiety for some participants  *Role of the professional**:* e.g. related to staff availability/attitude, professionals inclined to take the lead, instead of shared-decision making. | *Intervention characteristics**:* the intervention is simple, strength-based, uses life-domains, facilitates learning through language, objects and experience, stimulates equality, uniqueness and social processes. These aspects were all brought forward as being important during the design phase.   *Activation**:* participants were getting active through the activity wheel, possibly also because the wheel was action oriented, used active phrasing and helped to break the routine. Discussion of topics was facilitated by the activities. Activities that were physical (e.g. going somewhere) were most memorable.   *Other key terms in the qualitative results related to possible facilitators**:* allowed for flexible use, changed relationships with supportive others, allowed for reflection of the past, fun and easy. | *Format***: delivered by peer support workers. Additional suggestion by the authors: nurses often do not have enough time, identity-based interventions could also be delivered by occupational therapists or peer support workers, some changes in the content may be easier when the intervention is digitalized.   *Content*: increasing the* number of activities, design (more attention for nonverbal aspects), attention for more personalization.  *Extending target group*:* Other people who could benefit for an identity-based approach (e.g. after life changing physical injury or traumatic brain injury) |
| Two different SET (self-experiential) treatments in the United States (Muzekari et al., 1973) | *Emotional impact**: it is possible that the intervention had a negative impact on self-concept because it was too confrontational (e.g. confrontation with appearance and deficits was experienced negatively).   *Heightened awareness**: awareness of self-attributes may have increased attention to negative self-aspects and negative social comparisons, which can explain negative self-concept outcomes.   *Role of the therapist*:* therapeutic relationship may be hampered in this study because the role of the therapist was limited due to the experimental nature of the study and the focus on correcting participants. | *Group format*:* the self-experiential group treatment had a negative effect, but the control group -which did allow for interpersonal behavior, socialization and social interaction- did have favorable outcomes. | *Content*:* the intervention may have been confrontational, the impact of self-confrontation could get more attention, the therapists in the current intervention focused too much on instructions, more attention is needed for the therapeutic relationship and to help participants cope with their new insights and their feelings after seeing the videos. |
| G4H (Groups for health) in the United Kingdom (Hogg et al., 2025) | *Emotional impact**: for some participants, discussing past challenges and support in the past was stressful. Discussing the loss of important relationships also induced negative emotions but is not framed in the article as a barrier as it is considered an opening to discuss topics such as stigma.  *Group format*:* some individuals declined participation because they did not want to participate in a group. | *Content**: discussion of reasons why social groups are lost, social skills and toxic groups were often considered helpful.  *Hypothesized mechanisms*:* therapeutic alliance; group compatibility; identification with peers; integration of identity as a person with mental health challenges in the self-concept; empathy. | *Delivery*:* not all approached individuals wanted to participate due to COVID, for those individuals telehealth could be developed.  *Format*: i*n future trials it could be valuable to let participants choose if they want in individual or group format, as there was no clear difference in outcomes between these formats. If necessary, an individual session could be offered before the group intervention.  *Duration**:* most participants found the length of treatment sufficient, but eight wanted a longer duration and one a shorter duration. |
| ***SMI: interventions that less strongly target identity and self-views*** | | | |
| Self-government group in the United States (Cerniglia et al., 1978) | *Not mentioned* | *Hypothesized mechanisms**: self-determination through decision-making --> feeling of control and competence --> behavioral changes (e.g. more non-patient behavior) --> self-concept enhancement, feeling ‘normal’, decrease of disability-identity | *Implementation*:* Implement patient decision-making in treatment institutions from admission with increasing responsibilities for the participants. |
| Holistic hospital program in the United States (Lukoff et al., 1986) | *Intervention context*:* the focus of the intervention was on holistic health (including discussion of hallucinations, education about healthy food), but patients realized that hospital policy and staff attitudes conflicted with these principles (e.g. unhealthy meals, staff policy to disregard positive symptoms, token economy with candy/cigarettes as incentives to participate in interventions). Most of the staff members were not educated or did not practice/belief in holistic principles.   *Hypothesized barrier*:* the intervention was not followed up by a program in the community, which may account for the high relapse. | *Hypothesized mechanisms*:* motivation for participation increased through the token economy; physical exercise --> fitness. | *Content*: c*ombine the holistic treatment with other treatments such as social skills training and medication.   *Duration*: c*ontinued care from holistic principles after discharge to prevent relapse. |
| Mindfulness-based Cognitive Therapy for Psychosis in England (Randal et al., 2015) | *Intervention characteristics*:* the intervention was possibly not sufficient in helping participants to loosen their construal, as they showed tighter construal after the intervention (which may indicate more fixed construal/less therapeutic benefit, and may relate to anxiety). | *Hypothesized mechanisms**: MBCT may influence awareness of (positive) experiences and thereby influence recovery. MBCT may influence uptake of a new activity, which could help counteract negative self-views. Through mindfulness exercises, MBCT may impact self-understanding. MBCT may have helped participants to learn that they would like to recover. | *Therapist behavior*:* MBCT therapist could also discuss recovery, and personal meaning of recovery and goals with participants.   *Context/approach*:* loosening of construal may be aided by: diminishing anxiety, open-ended questions or brainstorming, using metaphors, discussing feelings, a relaxed atmosphere in the room (e.g. lights, seats, quiet talking) and asking about feelings. However, more evidence is needed and clinicians should be aware that too much loosening of construal may not be beneficial for people with a psychosis. |
| Dynamic cognitive intervention in Israel (Hadas-Lidor et al., 2001) | *Not mentioned* | *Hypothesized mechanism**: through the exercises the participants learned to cognitive strategies and his could influence daily living.   Nb. note that the expected relationship with self-concept could not be established in this study. | *Extending target group*:* the program should be offered according to need to people with schizophrenia who are in remission. |
| Cognitive Behavioural Therapy for residual symptoms in the United States (Bradshaw and Roseborough, 2004) | *Not mentioned* | *Duration of treatment*:* long duration may have positively impacted outcomes, especially functional outcomes and goal attainment. | *No explicit recommendations* |
| Transitional intervention to community based care in Canada (McCay et al., 2021) | *Not mentioned* | *Group format**:* sharing common experiences   *Intervention characteristics**:* structured, but enough flexibility to establish group relationships. Specific activities helped to reach goals (concrete advice, concrete/structured goal-setting, making a resume)   *Role of the professional***: someone who takes the time to listen, action-oriented, involvement (e.g. actively going with participant to places)   *Possible mechanisms**:* self-confidence through concrete goal-setting; agency and reduced engulfment through rediscovering a valued sense of self and challenging negative future perspectives   *Hypothesized mechanism *:* group intervention influenced hope/positive beliefs. Uncertainty about the future + losing supportive ties with practitioners seems to have increased motivation for to participate in the intervention. Positive was that the coach was: person-centered, goal-oriented, supportive, and flexible. Goal-oriented steps, being in a group and challenging negative expectations influenced sense of self (e.g. self-esteem / self-reliance / motivation /sense of self). | *No explicit recommendations* |
| ***ABI: interventions with a strong focus on identity and self-views*** | | | |
| Self-Concept group in the United States (Vickery et al., 2006) | *Participant characteristics**: fear of failure may negatively impact engagement with activities, especially if self-concept is not directly addressed in interventions. | *Hypothesized mechanism**: addressing self-concept (directly) may facilitate recovery and functional outcomes, through increased motivation and engagement in rehabilitation. | *Recommendations for general practice:* see facilitators. |
| Biographic–narrative intervention for aphasia in Germany (Corsten et al., 2015) | *Emotional impact**:* narrating the life story can be confrontational if participants remember negative events.  *Intervention characteristics***: group interventions cannot be planned flexibly around participants’ schedules.   *Group format**: for one participant, upward comparison may have caused diminished self-concept. | *Role of the professional**:* patience, taking time, trust, supporting communication.   *Group format**:* communicative abilities, peer contact, positive atmosphere. Upward and downward social comparisons and peer contact had a positive influence (e.g. on communication motivation, activation, relativation, normalization, inclusion, agency, control, identity renegotiation).   *Individual format**:* feeling in control, time to talk, individual communication support.  *Possible mechanism***: feeling competent (e.g. communication competence, resilient stories); meaningfulness (e.g. contributing to research); control (e.g. through activation).   *Hypothesized mechanism*:* Improvements in mood and quality of life mediated by identity processes: agency, control and social interaction. Keywords of mentioned processes are: biographical repair, restoring normalcy, activation, reframing health/illness, agency, integrating illness in self-concept, social comparison, competence/agency, focus on positive and negative events apart from the aphasia, combination of group and individual treatment, valued roles (e.g. participation in research). | *Duration**: duration should be over a ‘sufficiently long period’, as it may take some time before it has an effect on daily life.   *Format**: if it would be possible for participants to lead the groups themselves this may also have positive impact (on the peer leaders). This type of intervention may also be included in language therapy.   *Content**: more attention for self-defining/meaningful activities. E.g. include/develop tools to help people discover and act upon self-defining/meaningful activities. Input from caregivers is important in this regard. |
| My story project in the United States (Strong et al., 2018) | *Emotional impact**:* stories can have emotional impact. Clinicians should be prepared to undertake appropriate action if participants would become too emotional.   *Participant characteristics**: participants should be interested and want to share the story, as their stories are highly personal. | *Intervention characteristics***: humor, narrative co-construction (reflection about the past and about self-continuity). This allowed for communication opportunities (also with family) and hope.   *Hypothesized facilitators**: discussion about communication confidence, humor, co-construction, engaging participants in a process of hoping, time, response from the listeners.   *Other key terms in the qualitative results related to possible facilitators**:* thinking about the future and talking were meaningful. Talking about concerns gave new perspectives. It was a possible experience, which gave hope and communication confidence. | *Extending target group**: e.g. severe aphasia and women |
| Signature strengths intervention (part of positive psychology program) in Scotland (Andrewes et al., 2014) | *Participant characteristics**: cognitive difficulties may hinder learning/memorizing. Apathy and motivational issues make it difficult to plan sessions or individually support participants. Participant’s behavior (drug use and aggression) made it impossible to do the intervention in a group.   *Intervention characteristics**: signature strengths intervention was not in a group, although a group may have other positive effects. | *Intervention characteristics**:* Strengths intervention helped with activity planning and reflection about strengths, values and positive self-aspects, according to the occupation therapists.   *Positive emotion**:* strengths intervention was 'uplifting' and engaging according to the occupational therapists. | *Duration*:* more time may be needed to carry out the (take home) strengths exercise and implement strengths-based activities.   *Implementation*:* train staff and include the intervention in staff routine, use reminders or prompts via a phone or portable paging system.   *Format*:* the strengths-exercise was not carried out in a group, but a group could have beneficial effects.   *Content*:* more regular group discussions for patients about positive events. |
| Therapeutic song writing in Australia (Baker et al., 2015) | *Not mentioned (note: authors do mention barriers in protocol published elsewhere)* | *Intervention characteristics*:* narrative elements, music, exploration of multiple self-domains, focus on 'residual self'.   *Hypothesized mechanisms*:* music may activate memory consolidation, stimulation of autobiographical memory and activation of the mesolimbic system.  *Possible mechanisms**:* self-concept changed most in the first sessions about present and past self (hypothesis: it may be that self-concept changes most in the beginning of therapy, or that sessions about present and past have more impact than sessions about the future). Self-concept positively correlated with well-being outcomes, possibly because of the positive effects of song writing and attention to residual self.  *Note:* Note that the authors expected that flow and meaningfulness would be mechanisms of change. However, the outcomes of the current study did not support this (correlations were either nonsignificant or not in the expected direction). Meaningfulness was related to negative affect/anxiety and decrease of emotion suppression*,* which may be caused by emotional processing. | *No explicit recommendations* |
| Therapeutic song writing in Australia (Roddy et al., 2020) | *Participant characteristics**: more negative outcomes for participants with more severe disability. Maybe this has a relation with self-awareness (hopelessness, grief, identity-threat, perspectives on recovery). Note: musicality did not seem to be a barrier in this study. | *Possible mechanisms**:* there seems to be a relation between functional improvement and self-concept. | *Duration*:* more time may be needed to see effects on self and mood, especially for people with more severe disability. |
| Woman’s self-help group with a focus on identity and feminity in Canada (Gelech et al., 2019) | *Contextual**: identity change may be limited to the specific intervention context. | *Intervention elements*:* topics of strengths, wellness and gender, women-only group with two shared identities (gender and illness), focus on identity continuity, decentralizing illness-identity, growth, normalization, egalitarian principles. Participants discussed gender-related topics more and more towards the end of the intervention.   *Important intervention elements and mechanisms observed throughout the intervention***: reflective exercises (made participants focus on positive selves); participants shared things they made (possibly enabled by the strength-based focus and positive atmosphere); exercises and formal/informal discussions enabled participants to share their knowledge.  *Possible facilitators**: assumptions of competence, focus on dialogue and equality. Gender-segregation may have affected decentralizing illness-identity.   *Group format***: peer contact enabled: normalization, reinforcement of positive self-aspects, helping each other, ability to show competence, group comparisons, reinforcement, recognition of shared experiences, humor, validation of old selves, self-continuity, discussion of growth, discussion about resisting infantilization (was empowering). | *Extending target group*:* similar topics (gender, sexuality and collective identity) may also be relevant topics in (mixed gender) settings. |
| Online aphasia bibliotherapy group with a focus on discussing a book about adjustment and identity after stroke in the United States (Hoover et al., 2023) | *Participant characteristics***: Participants should be ready for the intervention, possibly this is not the case in early stages.   *Intervention characteristics***: differences between the participant and the author of the book made it difficult to relate to the story for some: gender, racial, socioeconomic, expressing negative emotion or not feeling the same hopefulness for the future. The focus of the book on professional roles was also not relatable for everyone. one person found that the book did not give new information. Reading can be challenging. One person also found that there were too many reflection questions.   *Emotional impact**:* some participants were anxious to relive emotion and reading about a chronic illness may have a negative impact on future perspective for those who are in an early stage of recovery.   *Format**:* online environment felt less safe for one person.   *Therapist characteristics**:* one participant felt the facilitators did not have enough life experience to help them reflect. | *Group format**: connection, community and normalization impacts wellbeing and identity.   *Group format**:* feeling of validation and being in it together, learning, self-efficacy and goal setting in a challenging situation, and feeling supported/understood.   *Comparing oneself with the author of the book**:* similarities and differences were food for thought. When people recognized the story, this validated their own experiences.  *Content**:* Gave hope, new perspective. Theories (e.g. Maslow) and other adult aphasia topics provided opportunity to learn, make sense/find meaning and feel empowered. Meeting the author was positive.   *Format***: reading a book helped improve reading skill, the adult tone was positive.   *Format**: Self-management is empowering and may influence self-efficacy and identity. Expected is that involvement in the book depends on interest in the topic and identification, and can affect the feeling of not being alone.   *Role of the professional**:* made the book accessible. | *Duration*:* unclear how the frequency or length of sessions could influence outcomes  *Participant characteristics***: in the first months after stroke participants might not be ready for the intervention/reading this book.   *Format*:* unclear if the effects would be similar in an in-person setting and with other group sizes. One participant thought an in-person setting could provide more privacy. Options for collaboration with other facilitators could be explored: e.g. social worker, librarian, volunteer or peers.   *Content***: not too many reflection questions.   *Content*:* unclear if the 'meeting the author' was a necessary component. The intervention could be adapted to the individual.   *Therapist education*:* unclear how much training is needed for facilitators. |
| Therapeutic song writing in the United States (Strong and Sather, 2024) | *Emotional impact**:* in the interviews the participants talked about diverse emotions, including sadness. The authors mention that diverse emotions, not only positive, were observed. Though from the results it is not clear if the negative emotions had a negative impact or were helpful. | *Hypothesized mechanisms*:* not only the songwriting, but the whole process was a catalyst of changes in emotions, feelings and behaviors. Specifically, the narrative approach can help to explore and integrate past/present/future self.  *Intervention elements*:* all active ingredients (songwriting, release party, participants) worked together to influence meaning, identity and engagement.  *Role of the professional**:* the focus of the intervention and professional team was on relationship-centered care impacted the person (e.g. feeling heard, validated) and the songwriting process.  *Intervention characteristics and activation**:* songwriting was a meaningful activity, it was meaningful to reflect on the past, with attention for both positive and negative emotions. The intervention also changed which activities participants did between sessions.  *Activation**: the authors highlight that the intervention provided tangible and actionable activities, which may support meaning making and independence.  *Positive emotion**:* the songwriting was enjoyable and gave rise to positive emotions and hope.  *Intervention content***: the songs about past, present and future (and links between these different selves) made participants reflect, e.g. on who they are, on their future. | x |
| ***ABI: interventions that less strongly target identity and self-views*** | | | |
| Interpersonal process recall (communication skill training) in the United States (Helffenstein and Wechsler, 1982) | *Not mentioned* | *Hypothesized mechanisms*:* Understanding of communication and behavior was aided by the immediate (intense an d auditory) video feedback and explanation and experience of how effective communication works. Improved interaction and improved social self and general self-concept --> might make participants less anxious | *No explicit recommendations* |
| Physical exercise in the United States (Brinkman and Hoskins, 1979) | *Not mentioned* | *Hypothesized mechanisms**: physical conditioning --> muscle / cardiovascular adaptation --> experienced as positive because participants experience (physical/functional) improvements through active participation --> self-concept enhancement | *No explicit recommendations* |
| Client-driven adjustment after ABI group in Ireland (Von Mensenkampff et al., 2015) | *Not mentioned* | *Hypothesized mechanism**: normalization and emotional processing of ABI facilitated by group attendance and validation of experiences. The group was client-driven and this made it possible to discuss topics that were relevant for the clients, such as the topic of acceptance of self.   *Possible mechanisms**:* the description ‘same but different’ helped to accept identity. Knowledge (e.g. understanding symptoms) influenced self-awareness and coping.   *Positive emotion**:* telling stories about feelings/thoughts and participation in the group, positively influenced emotion. Positive emotion influenced future thinking.   *Group format**:* meeting others was beneficial/helped with normalization, feeling not alone, accepting illness, accepting abilities. | *No explicit recommendations* |
| Peer support adjustment group in Canada (Cutler et al., 2016) | *Participant characteristics*:* intervention is developed only for high functioning participants, may not be suitable for other target groups. | *Intervention characteristics**: the group allowed for biographic repair and repair of sense of self, through validation and normalization (of feelings), sharing with peers and changing negative self-perceptions. The group was an unique place where they could discuss negative feelings.   *Hypothesized mechanisms*:* sharing accomplishments was related to self-confidence. Being in a group with similar others enabled normalization.  *Group format**:* group members were helpful in a way that was different from the help/understanding that family and friends could offer. Helping others and contributing to the group gave a sense of purpose.  *Other keywords from the qualitative analysis related to possible mechanisms**:* Adjustment through motivation, validation, normalization, connection, structure, information sharing and skills. Adapted sense of self through: social comparison, belief in capacity, reflection about strengths, purpose, self-acceptance, hope and accepting illness but not being defined by it. | *Extending the target group*:* researchers could test if a similar peer group could be relevant for inpatient rehabilitation.   *Context/atmosphere***: organize the group in a less clinical setting, e.g. a ´more organic´ place in the community.   *Format**: t*ake more time at the beginning of each meeting to get comfortable. Include homework. Include a peer who participated in an earlier group. |
| MFG (Multifamily Group intervention) in Australia (Kelly et al., 2013) | *Heightened awareness*:* possibly participants awareness of disability was heightened as a result of the group. Which may have an even larger effect as the disability did not change during the intervention.   *Duration*:* more time, repetition and opportunity to practice may be needed for changes. *Participant characteristics*:* memory and learning difficulties may have been a barrier, for example to recall the sessions and apply this in daily life. | *Not mentioned* | *Duration*:* more intensity and longer duration of the intervention, with more time for repetition and practice. Authors mention an example of an intervention that took 6 months of daily therapy, which may have more impact on self-concept than the current intervention.   *Format and content*:* include more active rehabilitation, activity in the community. For some participants (with long-term emotional adjustment difficulty) individual sessions may be more appropriate. Alternative approaches that may be used for self-concept include: using the Y-shaped rehabilitation model, focus mainly on skills or focus mainly on emotional adjustment |
| Recreational kayaking in Australia (Fines and Nichols, 1994) | *Not mentioned* | *Hypothesized mechanism*:* experience of success after being able to do something actively may positively influence identity and self-acceptance. | *No explicit recommendations* |
| Project-based intervention: developing and presenting an advocacy presentation in the United States (Hoepner et al, 2022) | *Participant characteristics*** not everyone wanted to be a presenter or felt comfortable representing the group, e.g. if their experiences or duration of illness differed.  *Intervention characteristics**: p*resentations that went less well could make participants insecure. | *Important intervention elements and mechanisms observed throughout the intervention**:* some of the speakers who did not feel comfortable with presenting did feel comfortable with a video contribution. Contributing to a meaningful project and being an international speaker and advocate made people proud. The presentation enabled different care providers to listen to the participants and their comments made participants feel heard. Practice sessions and constructive feedback helped to improve presentation skills. Positive experiences with presentations increased self-efficacy.   *Possible facilitators*:* focusing on non-problem saturated stories may have impacted identity | *No explicit recommendations* |
| VaLiANT: Valued Living After  Neurological Trauma in Australia (Sathananthan et al., 2025) | *Delivery**: online delivery of the intervention was difficult, especially for larger and more impaired groups and there was also less social interaction online than face-to-face.  *Group format***: less personalization | *Group format**:* connection to group members was an important mechanism.  *In person delivery*:* enabled connection and social interaction more than online delivery. | *Delivery**: in person instead of telehealth**,** time the intervention in such a way that it does not clash with other valued activities.  *Duration*:* more sessions might be helpful to sustain improvements.  *Duration**:* increase treatment length and provide a reunion/follow-up session.  *Format**:* more personalization.  *Format*:* another delivery model might be needed for sustained behavior change. E.g. booster sessions or adding individual sessions.  *Intervention development**: co-design together with individuals with ABI and practitioners. |
| ***ID: interventions with a strong focus on identity and self-views*** | | | |
| Narrative workshops for Mingle support group in England (Elderton et al., 2013) | *Participant characteristics**:* some members dominate the group and some remain mostly silent. | *Therapists’ behavior*:* share personal experiences, help participants focus on stories they do not often tell and alternative stories, externalize difficulties, listen to and support other members.   *Intervention content*:* the authors argue, based upon the literature, that it was helpful to: use photos and pictures (to connect to old and new stories, and to support storytelling and thick narratives), draw (to help participants develop new stories), and extensively prepare the presentation for a conference (to prevent a problem-saturated story).   *Group format**:* outsider witnessing, supporting the others, impact of hearing stories of other members, feeling connected. | *Format**: other people could be invited to listen to the stories as ´outsider witnesses´, e.g. people without intellectual disability who identify as LGBT. In case a group has a lot of differences in cognitive or verbal functioning (risk of some people dominating the group), the group could be split in smaller groups with similar levels of verbal and cognitive functioning. |
| ***ID: interventions that less strongly target identity and self-views*** | | | |
| Group mural painting project in the United States (Trzaska, 2012) | *Not mentioned.* | *Hypothesized mechanism*:* empowerment and self-esteem through gaining competence and skill, social reinforcement, feeling independent, having the possibility to learn through making mistakes and being supported in a non-judgmental social setting with not too many expectations. *Group format*:* dynamic group setting and emphasis on social interaction may have impacted self-esteem and social self-concept, identifying with peers may have helped with self-understanding.   *Intervention content*:* having to represent their thoughts and feelings in a concrete way and recognition of this by others may have impacted their personal identity. | *Duration*:* more sessions were needed to complete the mural than planned (minimum: 2x1 hour).  *Format*:* use of other materials or art projects could possibly have similar benefits. |
| Note that we only summarize the barriers, facilitators and recommendations that were mentioned by the authors (in the results or discussion section) or that were mentioned by the study participants. Only recommendations for intervention effectiveness or feasibility are summarized in this table (not about study methodology). | | | |
