# Supplementary material for: Interventions targeting identity in adults with psychosis, severe mental illness, brain injury, or intellectual disability: a transdiagnostic scoping review
Source: Front Psychiatry. 2026 Feb 5;17:1674898. doi: 10.3389/fpsyt.2026.1674898 (PMC12916650; doi:10.3389/fpsyt.2026.1674898)
Supplement: Supplementary file 6 [file SupplementaryFile6.docx]

**Figure 3**

*Strength of the evidence and most prominent limitations*


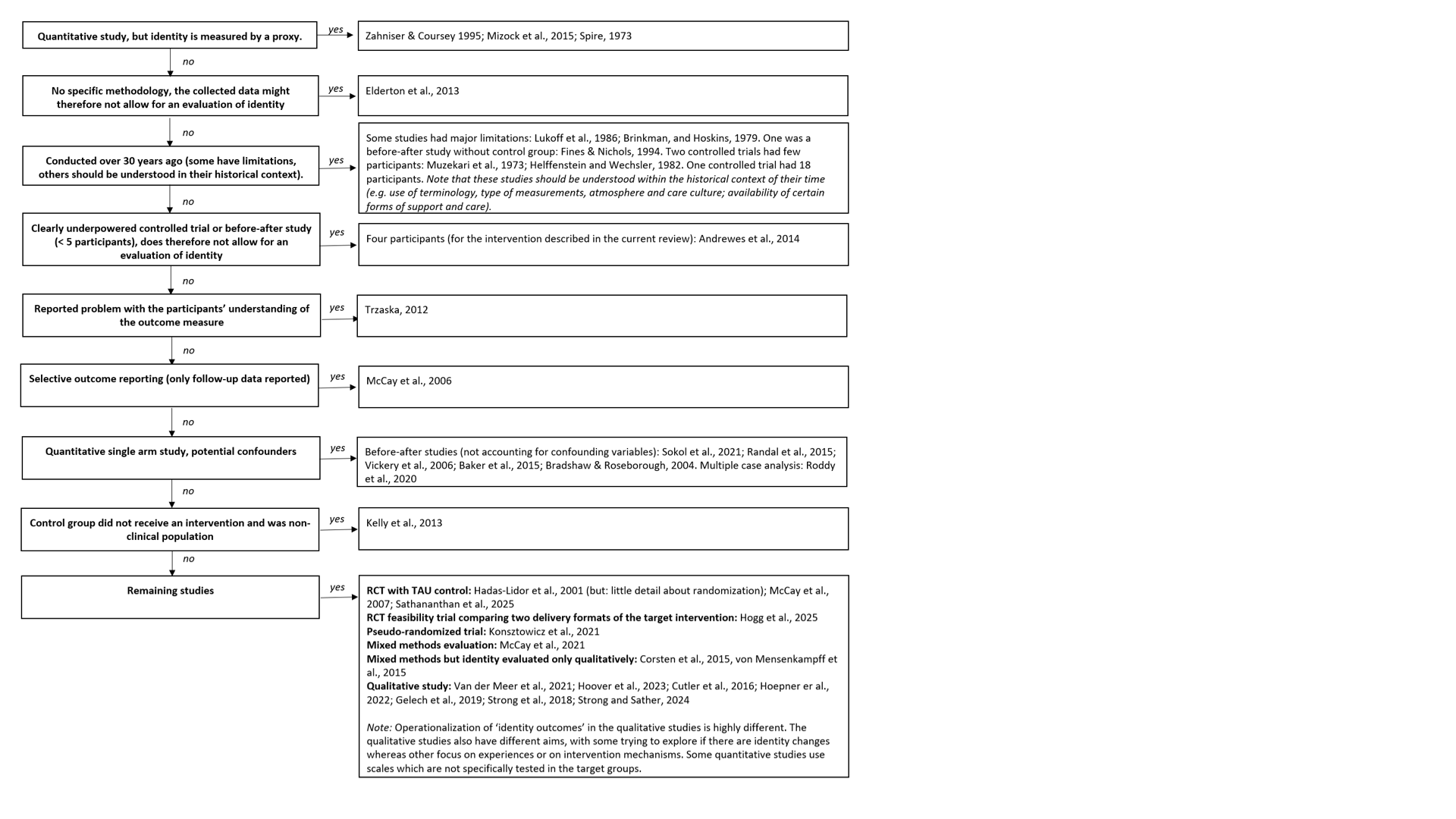


*Note.* The quality appraisal with the MMAT showed that most qualitative studies were of sufficient quality, but for many quantitative studies important limitations were reported. Three studies evaluated identity quantitatively through a proxy, one reported potential issues of participants who did not fully understand the identity outcome scale and signaled validity problems with the identity scale, and one qualitative study had weak methodological credibility. Some studies were conducted 20-50 years ago, using different terminology, ethical standards, and scientific methodologies; many of these studies had major limitations and must be understood within the historical context of their time. From the remaining studies, one was a quantitative pre-post intervention study with less than five participants, one was a quantitative pre-post intervention study which did not report all data, and six were quantitative studies which did not sufficiently accounted for confounding variables. Although seven studies reported random assignment to a control intervention or to treatment as usual, the randomization process was not described in three of them, and one RCT study used a control condition involving an alternative format of the target intervention.

**Table 6**

*Quality Assessment of Included Studies Based on the MMAT**

| **Target group** | **Study** | **MMAT Screening questions** | | **MMAT Methodological Quality Criteria** | | | | | **Other Reviewer Notes** |
| --- | --- | --- | --- | --- | --- | --- | --- | --- | --- |
| **Studies with a qualitative identity evaluation^[[1]](#footnote-1)^:** | **Reference; type of method** | **Are there clear research questions?** | **Do the collected data allow to address the research questions?** | **1.1. Is the qualitative approach appropriate to answer the research question?** | **1.2. Are the qualitative data collection methods adequate to address the research question?^[[2]](#footnote-2)^** | **1.3. Are the findings adequately derived from the data?** | **1.4. Is the interpretation of results sufficiently substantiated by data?** | **1.5. Is there coherence between qualitative data sources, collection, analysis and interpretation?** | **Notes** |
| SMI, direct interventions | Van der Meer et al., 2021; thematic analysis of interviews | Yes | Yes | Yes | Yes, but limited generalizability (possible selection bias, small sample size: possibly no data saturation/little diversity). | Yes | Yes | Yes | x |
| ABI, direct interventions | Corsten et al., 2015*; qualitative analysis of interviews with grounded theory | Yes | Yes | Yes, especially to understand mechanisms. | Yes | Yes, though it remains unclear which themes are derived deductively and which inductively. | Yes | Yes | x |
|  | Strong et al., 2018**; interpretative phenomenological analysis of interviews | Yes | Yes | Yes, but IPA is highly interpretative and is not suitable to evaluate identity outcomes, only to explore experiences (which are related to identity). | Yes, but limited generalizability (small sample size: possibly no data saturation/little diversity). Note: no female participants. | Yes | Yes | Yes | x |
|  | Gelech et al., 2019; Sociolinguistic interactional analysis of sessions | Yes | Yes | Yes, to understand the connection between intervention elements and identity processes. Not to evaluate participants personal evaluations. | Yes, but only one setting and few participantsit only describes one specific intervention with few participants. Note: only female participants. | Yes | Yes | Yes | x |
|  | Hoover et al., 2023; thematic analysis of interviews | Yes | Yes | Yes for the study. For the current review: no explicit aim to evaluate identity, though they do report about identity topics. | Yes | Yes | Yes | Yes | x |
|  | Strong and Sather, 2024; interpretative phenomenological analysis of interviews | Yes | Yes | Yes, but IPA is highly interpretative and is not suitable to evaluate identity outcomes, only to explore experiences (which are related to identity). | Yes, but limited generalizability (small sample size: possibly no data saturation/little diversity). The interview may have been somewhat leading, as suggested by the quotes. | Yes | Many quotes used, but these also show the limited verbal abilities of the participants, showing that the interpretation is highly interpretative. | Yes | Potential social desirability bias? |
| ABI indirect interventions | Cutler et al., 2016; directed content analysis (with descriptive-interpretive focus) of interviews. | Yes | Yes | Yes, to understand the role of peer support - but explicitly not to evaluate the effectiveness of the intervention. | Method seems adequate, but it is unclear why some participants are interviewed after one month and others after six months and which participants these are. | Yes | Yes | Yes | Is six months is not too long to explore the influence of the group on adjustmen? |
|  | Hoepner et al, 2022; interpretative description analysis of written feedback | Yes | Yes | Yes | Only to understand the participants for this current project, data saturation was not achieved. | Yes | Yes | Yes | x |
|  | Von Mensenkampff et al., 2015**; thematic analysis of interviews before and after intervention | Yes | Yes | Yes | Yes | Yes | Yes | Yes | There were specified themes in the intervention, but it was client-directed, thus the concrete topics in the different intervention groups were different. |
| ID direct interventions | Elderton et al., 2013 | Unclear if the aim is to describe or evaluate the study. The authors do refer to evaluating effectiveness when referring to measures and in the discussion. | Unclear: data allow for an exploration of changes in narratives and participants’ experiences, which is relevant for the current review, but data might not allow for an evaluation of the effectiveness. | No: the authors mention interviews and and a feedback form, but do not explicate how data were collected or analysed. | Can’t tell: no information about who participated in the feedback forms and which questions were asked. The facilitators made notes during the sessions to gather narratives, but there are no audio recordings. It remains unclear if stories are compared at before and after the series of workshops or at the start/end of each individual workshop. | Can’t tell: there is no information about the data-analysis. | No, reporting of the feedback interview with support workers and participants was very limited. The given examples do not always support the authors interpretation. The authors say that the stories after the session were showing 'newly discovered identities', but based on the given examples this is not clear (limited grounding). | Can’t tell, there is too little information about the methodology. |  |
| **Studies with quantitative identity evaluation (randomized)^[[3]](#footnote-3)^** | **Reference; type of method** | **Are there clear research questions?** | **Do the collected data allow to address the research questions?** | **2.1. Is randomization appropriately performed?** | **2.2. Are the groups comparable at baseline?** | **2.3. Are there complete outcome data?** | **2.4. Are outcome assessors blinded to the intervention provided?** | **2.5 Did the participants adhere to the assigned intervention?** | **Notes** |
| SMI direct interventions | McCay et al. 2007; RCT | Yes | Yes | Can’t tell: The text mentions random allocation by pulling from an envelope, but gives no details on how envelopes were prepared or concealed. | Yes, with note that: the authors planned a 2:1 ratio, but in the end at baseline they end up with 41 versus 26 participants in the groups. The control group had lower negative symptoms at baseline, which was used as a coveratiate for some analyses but not for the identity analysis. | No: 30% drop-out | No: Therapists, participants and data analysist are not blinded. | Can’t tell. The participants who dropped out are not analyzed, not reported if those who remained in the study went to all sessions. | The authors note that the study is part of a longitudinal project, but data on phase 2 of the RCT is (as far as we know) not yet published. |
|  | Muzekari et al., 1973; possibly pseudo-randomized? | Yes | Can’t tell, participant group might be too small for sufficient power (n = 11) for efficacy analysis | Can’t tell: no information – therefore this study might be better considered pseudo-randomized. Participants were purposefully selected. | Can’t tell, not mentioned. Note that not only the content of the interventions was different, but also the group leaders. | Can´t tell: not mentioned. | Can´t tell: not mentioned. | Can´t tell: not mentioned. | The study takes place in the 1970's and the research context is substantially different: e.g. photography was new, some participants never saw themselves in a large mirror or photograph, not all participants may have gotten treatment, use of the word ‘schizophrenics’ was normal. |
| SMI indirect interventions | Lukoff et al., 1986; possibly pseudo-randomized? | Yes | Can’t tell, participant group might be too small for sufficient power (n = 14) for efficacy analysis | Can’t tell: no information – therefore this study might be better considered pseudo-randomized. | Can’t tell, not mentioned. | Can´t tell: not mentioned. | Can´t tell: not mentioned. | Can’t tell: not mentioned if the participants adhered or only came for hte token rewards. It should be noted that hte intervention was not given in ideal cirmcumstances: Most of the staff members did not practice/belief in holistic principles that they had to share with patients. | The study takes place in the 1980's and therefore the research context is substantially different. Participant details are not reported. Note that The staff did not believe in the intervention mechanisms and the staff attitudes and hospital policy was also in conflict with the intervention. There was a token economy, which meant that patients were rewarded with candy or cigarrettes for attending sessions about healthy eating. |
|  | Hadas-Lidor et al., 2001; possibly pseudo-randomized? | Yes | Yes | Can’t tell: no information – therefore this study might be better considered pseudo-randomized. Randomization is stratified. | Yes: equal groups matched for gender, age, family status, education and subcategory of schizophrenia diagnosis. | Drop-out is acceptable (19%) | Can´t tell: not mentioned. | Can’t tell. The participants who dropped out are not analyzed, not reported if those who remained in the study went to all sessions. | N = 29: might be underpowered. Duration of the intervention is over one year, so both groups might improve naturally. |
|  | Hogg et al., 2025; feasibility RCT | Yes | Only to evaluate feasibility: two groups are relatively small (n = 14 and n = 16 at follow-up). This study compared individual vs group formats, so there was no other control-group to evaluate efficacy of G4H. The identity outcome of interest for the current review was not analyzed statistically. | Yes | Yes | For the analysis (T1-T3) yes, but not for follow-up (which was therefore not anlayzed). | No: Therapists, participants and data analysist are not blinded. | Yes | The identity integration scale was difficult for some participants. |
| ABI indirect interventions | Helffenstein and Wechsler, 1982; possibly pseudo-randomized? | Yes | Can’t tell, participant group too small for sufficient power (n = 8) for efficacy analysis | Can’t tell: The text mentions use of a random number table, but gives no no information about concealment. | Yes, (except maybe for anxiety) | Not for follow up (64% drop-out) | Can´t tell: not mentioned. | Can´t tell: not mentioned. | x |
|  | Sathananthan et al., 2025; pilot RCT | Yes | Yes | Yes, with note that randomization used permuted blocks but block sizes were changed mid-trial from fixed (6) to variable (3, 6, 9) to improve allocation concealment. In 7% of the cases unblinding was reported. | Yes | Yes | Yes | Yes | x |
| **Studies with quantitative identity evaluation (non-randomized)** | **Reference; type of method** | **Are there clear research questions?** | **Do the collected data allow to address the research questions?** | **3.1. Are the participants representative of the target population?** | **3.2. Are measurements appropriate regarding both the outcome and intervention (or exposure)?** | **3.3. Are there complete outcome data?** | **3.4. Are the confounders accounted for in the design and analysis?** | **3.5. During the study period, is the intervention administered (or exposure occurred) as intended?** | **Notes** |
| SMI: direct interventions | Sokol et al., 2021; single arm before–after study. | Yes | Yes, with note that the participant group is relatively small (n = 17) | Not representative for current review: Participants were staff-referred with no details on selection criteria. Although diagnoses were relevant, the sample was a very specific veteran group, and the authors did not report efforts to achieve representativeness. Also relatively small sample (n = 17). | Validity and reliability of the outcome measure is adequate in a general population, but the FSCQ is not validated in target groups with a (severe) mental illness. | Drop-out is acceptable (19%) | No—this was a simple pre-post design. Identity changes correlated with suicidality, but many participants were moderately suicidal at baseline, and without a control group the observed changes could reflect time effects or other concurrent interventions. | Can’t tell: there was no adherence measure. 17 out of the 21 participants completed the intervention, but some participants were disruptive or did not engage as much with the materials as others. The intervention was part of a partial hospitalization program, so it is unclear if there were unplanned other interventions. |  |
|  | Mizock et al., 2015; single arm before-after study | Yes | Can’t tell, participant group too small for sufficient power (n = 12) for efficacy analysis. | No: Sample too small for power (n = 8 who filled in the same measure.) Can’t tell if these participans are representative: no details about the participants except diagnosis and ‘significant impairments’. | No. Identity is measured by a proxy and also not in all participants (In eight participants identity is measured by proxy with the The Ryff Scale of Psychological Well-Being, in another eight participants who participated in the second wave this measure was not used). | No, only half of the participants completed the outcome measure. | No—this was a simple pre-post design. | No: 31% of the participants did attend less than half of the sessions and three participants (19%) did not make any photovoice works. | The study reports participant feedback on identity effects, but no qualitative methodology is described to support or systematically analyze these observations. |
|  | Zahniser and Coursey 1995; non-randomized controlled trial. | Yes | Yes | No: It is unclear whether the target group is representative. The study dates from 1995 and care may have changed. Stratified randomization was not used in all participants, allocation bias is possible, and no efforts to ensure representativeness are reported. | No: Identity is measured by a proxy (The Rosenberg Self-Esteem Scale and The Self Confidence subscale of the Self-  Rating Scale). The statistical significance level is also not mentioned. | Can’t tell (not reported) | Can’t tell: there was a control group, but unclear if they did a direct comparison. They say differences within the groups were significicant, but do not analyze confounders. | Can’t tell: There was a study protocol, but there were different therapists and therapists adherence is not mentioned. It is not mentioned if any participant dropped out and if participants attended sessions. | The study reports participant feedback on identity effects, but no qualitative methodology is described to support or systematically analyze these observations. |
|  | McCay et al., 2006; non-randomized controlled trial | Yes | Yes | No: Only young individuals between 18-35 could participate, however the authors note later that the mean age ranged from 17-42? There was sequential assignment. The authors do not report how they tried to achieve a representative sample. Participants were recruited before 2000, the organization of care for individuals with SMI may have changed substantially since then. | No: there is selective outcome reporting, only outcomes at follow-up are reported, although outcomes directly after intervention are also measured. | Drop-out is acceptable. | Can’t tell: there was a control group (sequential assignment), but no statistical group comparison. There were significant differences between the intervention group and control group for gender and employment, but this did not correlate with outcome measures. | Can’t tell. Group leaders were trained and there was a standardized group manual and the intervention was recorded and leaders received feedback. But it is not mentioned if participants adhered to the intervention or if they had unplanned co-interventions. |  |
|  | Konsztowicz et al., 2021; non-randomized controlled trial | Yes | Yes, with note that the participant group is relatively small (n = 18) | The population is clearly described, and reasons for non-participation are mentioned. Note that only individuals with high engulfment were recruited and that only one participant was in-patient. The participant group is small. | Yes, with note that the MES does measure a specific identity-related outcome (illness engulfment) and that the measure translated and the translated version was not validated. | Drop-out is acceptable. | Can’t tell: there was a control group (sequential assignment) to TAU, but the intervention group had shorter illness duration, lower engulfment and lower depression at baseline. Engulfment outcomes were controlled for baseline score, but not for illness duration or depression. | Can’t tell, the authors report that no fidelity measure was used. Adherence by participants not mentioned. | The study reports participant feedback on identity effects, but no qualitative methodology is described to support or systematically analyze these observations. |
|  | Spire, 1973; single arm before-after study | Yes | Can’t tell, participant group might be too small for sufficient power (n = 12) for efficacy analysis. | No: participants are purposefully selected, but unclear based on which criteria. There is little information about the participant characteristics. Only women were included. Note that the study takes place in the 1970's and the research context is substantially different (e.g. therapeutic approaches, use of language, availability of mirrors/camera's). The participant group is small). | No: measures are outdated and not validated. the draw-a-person test was used in the 20th century, but its usefulness and validity has long been contested. The author used self-established criteria, which are not described. The authors also interpret changes in heterosexuality favorably. | Can’t tell: not reported | No, confounders were not addressed. Notably, one participant reported liking the intervention mainly because it provided personal attention otherwise lacking from staff, suggesting effects may reflect attention rather than the intervention itself. Participants lived in an institution, but it is not mentioned if they received other interventions concurrently. | Can’t tell, not mentioned. |  |
| SMI indirect interventions | Cerniglia et al., 1978; group homes were randomly allocated | Yes | Yes, with note that the participant group is relatively small (n = 18) | No: The study takes place in the 1970's and therefore the research context is substantially different. The researchers purposefully selected specific group homes to participate because of the type of residents, but do not further explain this | Can’t tell: Reliability analyses of the TSCS scale for the SMI population have not been conducted. | Can’t tell: not mentioned. | No: Group homes were randomly allocated to one of the conditions. It is possible that the specific group home had an influence on the outcomes. The group homes were purposefully selected. The experimental group had higher self-concept before intervention than the controls. | Can’t tell, not mentioned. It is likely that co-interventions were offered in certain homes. | Duration of the intervention is not reported. |
|  | Randal et al., 2015; single arm before-after study | Yes | Can’t tell, participant group might be too small for sufficient power (n = 11) for efficacy analysis. | Can’t tell participants were referred by their care co-ordinator. It is not mentioned which participants declined participation. The participant group is small. | Can’t tell, the study used a custom repertory grid to explore participants’ views of self, others, and experiences of psychosis. While related approaches have been used in the target population the reliability and validity are unknown, and the method may be difficult or less feasible for participants with cognitive difficulties. There are also questions about the interpretatation: e.g. does the construal of the grids mean that self vs ideal self really changed or just that participants used different terms after the intervention? It also remains unclear what the changed ‘salience of self’ means. | No: 48% of the intervention participants did not complete measures. | No, confounders were not addressed. | Can’t tell, not mentioned. | x |
|  | Bradshaw and Roseborough, 2004; single arm before-after study | Yes | Yes, with note that the participant group is relatively small (n = 22) | Can’t tell: Inclusion criteria are clearly described, but not mentioned which participants declined participation. Relatively young population: 26-46 years. Relatively small target group (n = 22). | The MES is appropriate, with note that it does measure a specific identity-related outcome (illness engulfment) | Yes: 27% drop-out is acceptable after 1.5 year. | No: Baseline trendlines are provided only for measures of symptoms and psychosocial functioning, not for the identity outcomes. The authors note possible confounders—low-intensity psychiatric and case-management contact, medication effects, and increased client attention—but consider the first two unlikely to affect outcomes, while the impact of increased attention cannot be ruled out due to the lack of a comparison group | Can’t tell: there were concurrent interventions (e.g. with a case manager). Some participants also changed medication. The authors report that therapist quality, assessed with the CTRS, was consistently high. | x |
| ABI: direct interventions | Vickery et al., 2006; single arm before-after study | Yes | Yes, with note that the participant group is relatively small (n = 18) | Can’t tell: Inclusion criteria are clearly described, but not mentioned which participants declined participation. Relatively small participant group. | Yes, with notes (e.g. language ability influence? See: [Website HISDS-III.pdf](https://movingahead.psy.unsw.edu.au/documents/research/outcome%20measures/adult/Measures%20of%20Self/Website%20HISDS-III.pdf)). Note that in the current study only a measure of the present self was used. | Can’t tell: not mentioned. | No: Participants received concurrent interventions and there was no control group. | Can’t tell: There was a study protocol, but there were different therapists and therapists adherence is not mentioned. It is not mentioned if any participant dropped out and if participants attended sessions. | x |
|  | Andrewes et al., 2014; single arm before-after study | Yes | Not for questions of efficacy in changing identity (only 4 participants). | No: only males, all with challenging behaviour. 80% had a history of substance abuse. Only 4 participants for the identity evaluation. | Yes | No: only few participants did the identity intervention and identity outcomes were not measured in the control group. | No: there is no data from the control group for the identity measure. Participants also received concurrent interventions. | No, in the first week adherence to the take home activity was poor, and that the intervention was therefore extended. It is not clear if adherence improved afterwards | x |
|  | Baker et al., 2015; single arm before-after study | Yes | Can’t tell, participant group might be too small for sufficient power (n = 10) for efficacy analysis. | Inclusion criteria and reasons for non-participation are clearly described, but for the current review the Limited for the current review: only 5 participants had an ABI. | Yes, with notes (e.g. language ability influence? See: [Website HISDS-III.pdf](https://movingahead.psy.unsw.edu.au/documents/research/outcome%20measures/adult/Measures%20of%20Self/Website%20HISDS-III.pdf)). Note that in the current study only a measure of the present self was used. | Yes, dropout rates are acceptable. Three participnants were exluded from the anlaysis due to missing data. | No: there was no control group. Some mechanisms of change were tested, but confounders were not accounted for. | Can’t tell: not mentioned. | x |
| ABI indirect interventions | Brinkman and Hoskins, 1979; single arm before-after study | Yes | Can’t tell, participant group might be too small for sufficient power (n = 7) for efficacy analysis. | Can’t tell. Note also that it is a relatively old study though and the research context should therefore be taken into account. | No: the authors use a 0.10 significance level. Reliability analyses of the TSCS scale for the ABI population have not been conducted , convergent validity is tested in this target group (see: [Website TSCS-2.pdf](https://movingahead.psy.unsw.edu.au/documents/research/outcome%20measures/adult/Measures%20of%20Self/Website%20TSCS-2.pdf)) | Yes, dropout rates are acceptable. | No: there was no control group and confounders not accounted for. | Good adherence reported for 5/7 participants. |  |
|  | Kelly et al., 2013; study with non-clinical control group | Yes | Yes | Can’t tell: Inclusion criteria are clearly described, but not mentioned which participants declined participation. | Can’t tell: Reliability analyses of the TSCS scale for the ABI population have not been conducted , convergent validity is tested in this target group (see: [Website TSCS-2.pdf](https://movingahead.psy.unsw.edu.au/documents/research/outcome%20measures/adult/Measures%20of%20Self/Website%20TSCS-2.pdf)) | Can’t tell, not mentioned. | No: there was a control group but this was a non-clinical comparison group which also had higher self-concept scores. | Can’t tell, not mentioned. |  |
|  | Fines and Nichols, 1994; single arm before-after study | Yes | Can’t tell, participant group might be too small for sufficient power (n = 7) for efficacy analysis. | No: 27 participants were eligible but only eight were chosen to participate, partly based on convenience. | Can’t tell: Reliability analyses of the TSCS scale for the ABI population have not been conducted , convergent validity is tested in this target group (see: [Website TSCS-2.pdf](https://movingahead.psy.unsw.edu.au/documents/research/outcome%20measures/adult/Measures%20of%20Self/Website%20TSCS-2.pdf)) | Yes | No: there was no control group and confounders not accounted for. | Yes | x |
| ID indirect interventions | Trzaska, 2012; single arm before-after study | Yes | Can’t tell, participant group might be too small for sufficient power (n = 10) for efficacy analysis. | Can’t tell: It is stated that only 10 individuals from the 98 contacted individuals met inclusion criteria, but it is not stated why some did not want to participate. | No: There were highly elevated scores on the ‘faking good scale’ which can indicate high social desirability of answers. Participants also did not understand all of the TSCS questions. Reliability analyses of the TSCS scale for adolescents with ID have indicated low to moderate reliability (Lund et al., 1981^[[4]](#footnote-4)^), we haven’t been able to find more recent studies. | Yes, but high drop-out in the comparison group | Can’t tell: There is a control group, but the participants in the control group are those participants who ‘did not meet all  inclusion criteria but agreed to participate’. | Yes, with note that the intervention was prolongued for all participants. | x |
| **Studies with quantitative descriptive identity evaluation** | **Reference; type of method** | **Are there clear research questions?** | **Do the collected data allow to address the research questions?** | **4.1. Is the sampling strategy relevant to address the research question?** | **4.2. Is the sample representative of the target population?** | **4.3. Are the measurements appropriate?** | **4.4. Is the risk of nonresponse bias low?** | **4.5. Is the statistical analysis appropriate to answer the research question?** | **General note** |
| ABI direct interventions | Roddy et al., 2020; descriptive case series analysis | Yes | Yes, but only to explore emerging trends, the participant group is too small for efficacy evaluation | No: Although the study recruited participants meeting the inclusion criteria through convenience sampling, it is unlikely that only five eligible individuals were available over a three-year period, and the basis for choosing these specific participants is not clarified. | No: Only male participants. It is not mentioned if/why some eligible individuals are not participating. | Partly yes: TSCS: Reliability analyses of the TSCS scale for the ABI population have not been conducted , convergent validity is tested in this target group (see: [Website TSCS-2.pdf](https://movingahead.psy.unsw.edu.au/documents/research/outcome%20measures/adult/Measures%20of%20Self/Website%20TSCS-2.pdf)) HISDS: yes, with notes (e.g. language ability influence? See: [Website HISDS-III.pdf](https://movingahead.psy.unsw.edu.au/documents/research/outcome%20measures/adult/Measures%20of%20Self/Website%20HISDS-III.pdf)). Note that in the current study only a measure of the present self was used. | Can’t tell. Not mentioned who declined participation. Mid-intervention evaluations are not present for two participants, but pre/post intervention measurements are present for all participants. | Sufficient for a descriptive study, but there is no statistical analysis. Authors clearly show the findings and changes per participant. It only reports on emerging trends. Some qualitative data are also reported, but only about the song-texts and this is not formally evaluated. | Note that participants received concurrent interventions, which are not taken into account in the analysis |
| **Studies with quantitative and qualitative identity evaluation** | **Reference; type of method** | **Are there clear research questions?** | **Do the collected data allow to address the research questions?** | **5.1. Is there an adequate rationale for using a mixed methods design to address the research question?** | **5.2. Are the different components of the study effectively integrated to answer the research question?** | **5.3. Are the outputs of the integration of qualitative and quantitative components adequately interpreted?** | **5.4. Are divergences and inconsistencies between quantitative and qualitative results adequately addressed?** | **5.5. Do the different components of the study adhere to the quality criteria of each tradition of the methods involved** | **General note** |
| SMI indirect interventions | McCay et al., 2021; mixed methods evaluation | Yes | Yes | Yes | Yes | Yes | Yes, with note: The authors acknowledge a divergence, suggesting that identity scores may not have changed significantly even though participants achieved positive outcomes. They compare end-of-treatment scores with those reported elsewhere, but do not reflect on potential differences between how identity was conceptualized in the qualitative evaluation and how it was operationalized in the quantitative scale. | Yes, overall sufficient, with note that there is relatively high drop-out (17 participants from the 27 completed the intervention and 15 filled in the follow-up measures, but of those 5 already dropped out before the intervention started). A prospective cohort is used, so there is a comparison group. The qualitative methods are adequately described and findings sufficiently grounded. | Note that the authors expect that participants already had low engulfment at the start of the intervention. |

* Hong, Q. N., Pluye, P., Fàbregues, S., Bartlett, G., Boardman, F., Cargo, M., Dagenais, P., Gagnon, M.-P., Griffiths, F., Nicolau, B., O’Cathain, A., Rousseau, M.-C., & Vedel, I. (2018). Mixed Methods Appraisal Tool (MMAT), version 2018. Canadian Intellectual Property Office, Industry Canada. Registration of Copyright #1148552.

**Also used a quantitative evaluation, but not related to identity.

1. Overall: few studies with a comparable method, target group and intervention**.**. [↑](#footnote-ref-1)
2. General note for all qualitative studies: these studies included participants who had disabilities which may have made it made it difficult to put their experiences into words, but the qualitative evaluations relied heavily on verbal report. [↑](#footnote-ref-2)
3. Five studies used a randomized control condition. These might not all be considered typical RCT’s. For three studies it is stated that individuals are randomized, but little information is provided. In these papers the term ‘RCT’ is also not used. In one study there is no typical control group, but two versions of the same intervention are compared. [↑](#footnote-ref-3)
4. Lund NL, Carman SM, Kranz PL. Reliability in the use of the Tennessee Self-Concept Scale for educable mentally retarded adolescents. J Psychol. 1981;109(2):205–11. [↑](#footnote-ref-4)
